# A cluster of long non-coding RNAs exhibit diagnostic and prognostic values in renal cell carcinoma

**DOI:** 10.18632/aging.102407

**Published:** 2019-11-14

**Authors:** Gong Cheng, Di Liu, Huageng Liang, Hongmei Yang, Ke Chen, Xiaoping Zhang

**Affiliations:** 1 Department of Urology, Union Hospital, Tongji Medical College, Huazhong University of Science and Technology, Wuhan 430022, China; 2 Institute of Urology, Union Hospital, Tongji Medical College, Huazhong University of Science and Technology, Wuhan 430022, China; 3 Department of Pathogenic Biology, School of Basic Medicine, Tongji Medical College, Huazhong University of Science and Technology, Wuhan 430022, China

**Keywords:** renal cell carcinoma, long non-coding RNA, biomarkers, metabolism

## Abstract

Kidney cancer ranked in the top 10 for both men and women in the estimated numbers of new cancer cases in the United States in 2018. Targeted therapies have recently been administered to patients with clear cell renal cell carcinoma (ccRCC), but the overall survival of patients at the terminal stage of the disease has not been as good as expected. It is therefore necessary to uncover efficient biomarkers for early diagnosis, and to clarify the molecular mechanisms underlying ccRCC progression and metastasis. Increased evidence has shown that long non-coding RNAs (lncRNAs) play important roles during tumor progression. In this study, 10 candidate lncRNAs with diagnostic and prognostic values in ccRCC were identified: IGFL2-AS1, AC023043.1, AP000439.2, AC124854.1, AL355102.4, TMEM246-AS1, AL133467.3, ZNF582-AS1, LINC01510 and PSMG3-AS1. Enrichment analysis revealed metabolic and functional pathways, which may be closely associated with kidney cancer tumorigenesis. Six representative processes were summarized, namely glycolysis, amino acid metabolism, lipid synthesis, reductive carboxylation, nucleotide metabolism, transmembrane transport and signal transduction. In combination, the present results provided prognostic and diagnostic biomarkers for ccRCC and might pave the way for targeted intervention and molecular therapies in the future.

## INTRODUCTION

Each year, the number of new cancer cases and cancer-related mortality occurring in the United States is estimated by the American Cancer Society. In 2018, kidney cancer ranked in the top 10 for both men and women in the estimated new cases, and accounted for 3% of the estimated male mortality [1]. Clear cell renal cell carcinoma (ccRCC) is the most common type of RCC. It is characterized by inactivating mutations in the VHL gene [2] and is associated with a high risk of metastasis and poor response to chemotherapy [3]. Approximately 46% of patients with ccRCC in modern surgical series are asymptomatic, with the tumor diagnosed incidentally during abdominal radiological imaging for unrelated symptoms [4]. Despite significant advances in its diagnosis and treatment, patients with ccRCC tend to have a poorer prognosis (5-year disease specific survival rates of 50-69%) than patients with other histological subtypes of RCC [5, 6]. Postoperative metastasis or local recurrence occurred in 20–40% of patients [7, 8]. Targeted therapies have recently been administered to patients with ccRCC, but the overall survival (OS) of patients at the terminal stage of the disease has not been as good as expected [9]. It is therefore necessary to uncover efficient biomarkers for early diagnosis and clarify the molecular mechanisms underlying ccRCC progression and metastasis.

Non-coding RNAs (ncRNAs) are transcripts without a protein coding potential, but play a crucial role in various cellular and physiologic processes [10]. Long ncRNAs (lncRNAs), which are >200 nucleotides long, have recently been shown to function as oncogenes or suppressors during tumorigenesis [11]. Apart from functional effects on tumor progression, lncRNAs also exhibit various modulatory roles through different mechanisms, including translation of protein-coding genes, epigenetic transcriptional modulation, remodeling of and interactions with chromatin, genome defense or RNA turnover [12, 13]. Based on these findings, dysregulated expression patterns of lncRNAs in cancer development indicate that these lncRNAs may act as potential molecular biomarkers [14]. In addition, recent studies have discovered connections between drug resistance and non-mutational modulation of gene expression, during which lncRNAs could function as major modulators and affect drug sensitivity to cancer cells [15]. Considering the highly specific phenotype of cancer types, lncRNAs have been considered not only as biomarkers, but also as remarkable therapeutic targets for cancer treatment.

To date, ~200 studies have reported the role of lncRNAs in RCC, most of which concentrated on the functional investigation of a single gene. Certain studies have revealed that several lncRNAs act as biomarkers in papillary RCC, chromophobe RCC and ccRCC [16–25]. However, these studies used unpaired normal and tumor samples, and the 5-10 times smaller unmatched normal sample size possibly created biasness. It is therefore preferable to use paired normal and tumor samples when screening for a large number of lncRNAs. Few reports have focused on the relationship between lncRNAs and the clinicopathological characteristics of ccRCC, which ignored the potentially prognostic and diagnostic value of lncRNAs in the clinic. Therefore, in this study, we tried to distinguish differentially expressed lncRNA patterns and determine their link to clinicopathological characteristics of ccRCC. We also further explored the functional roles of candidate lncRNAs and predicted potential pathways associated with ccRCC progression, which could pave the way for targeted intervention and molecular therapies in the future.

## RESULTS

### Identification of differentially expressed lncRNAs in ccRCC cells

**Figure 1:**
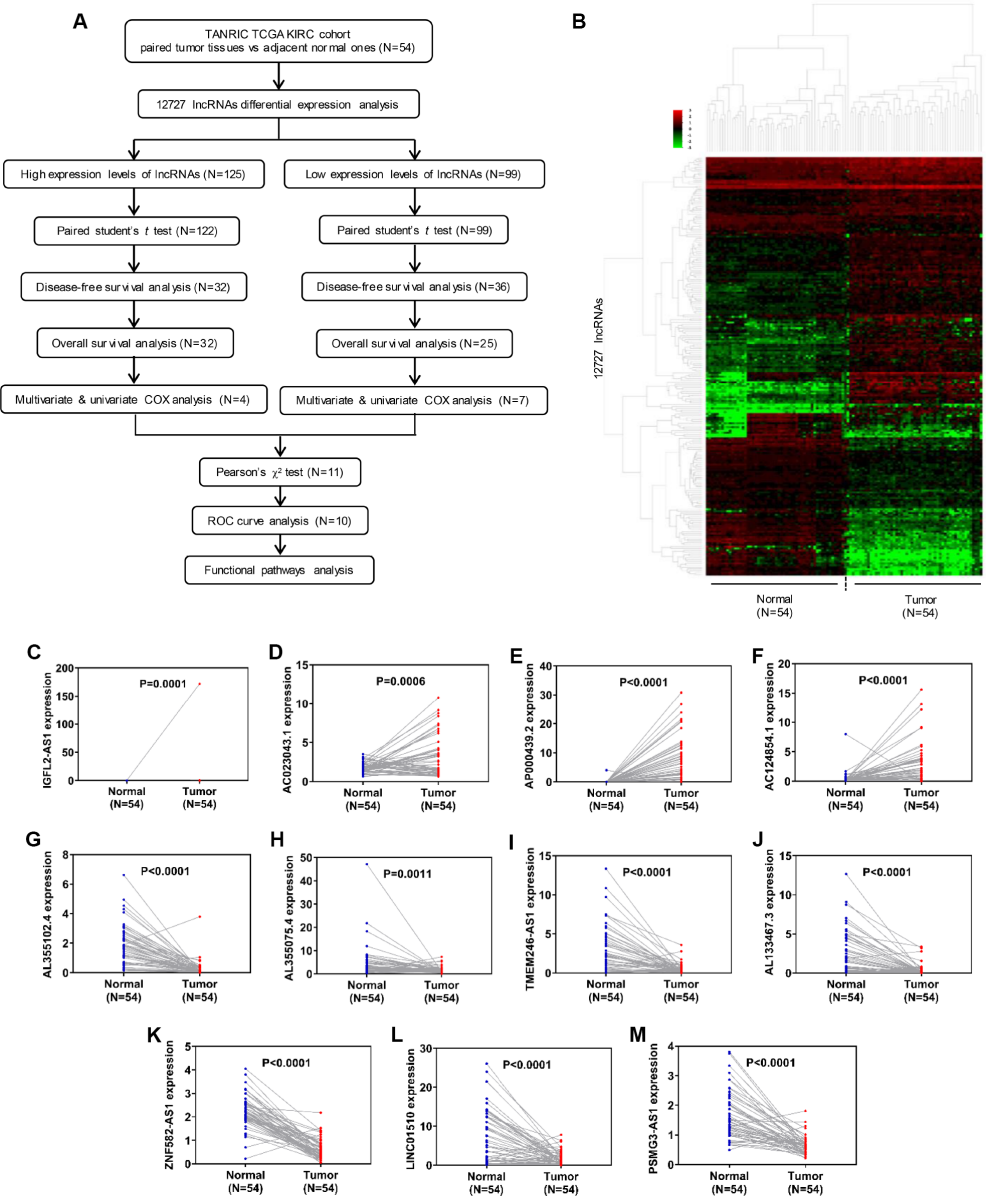
**Identification of differentially expressed lncRNAs in clear cell renal cell carcinoma.** (**A**) Flow chart of study design. (**B**) Hierarchical clustering heatmap analysis of 12,727 lncRNAs with 54 paired samples from the TANRIC database. (**C**–**M**) Paired student’s *t*-test of candidate lncRNA relative expression between 54 paired normal and tumor samples. lncRNAs, long non-coding RNAs.

The flow chart in Figure 1A was created in order to break down the present study step by step. LncRNA expression profiles were downloaded from the TANRIC database, which included 12,727 lncRNAs and 515 patient samples. Fifty-four paired normal and tumor samples were included in the cohort, and hierarchical clustering heatmap analysis was performed using R package ‘edgeR’ with |log_2_FC|>1, adjusted P<0.05 and FDR<0.01 (Figure 1B). The analysis identified 125 upregulated and 99 downregulated lncRNAs, which indicated that these differentially expressed genes could distinguish ccRCC from normal tissues (Supplementary Tables 1–2). To validate these results, we further examined the relative lncRNA expression levels between 54 normal and tumor samples using paired student's *t*-test. Out of the 125 upregulated lncRNAs, a statistically significant difference in relative expression was identified in 122, while all 99 of the downregulated lncRNAs passed the statistical tests (Figure 1C–1M, Supplementary Figures 1–4).

### Candidate lncRNAs act as potentially prognostic biomarkers for ccRCC

**Figure 2:**
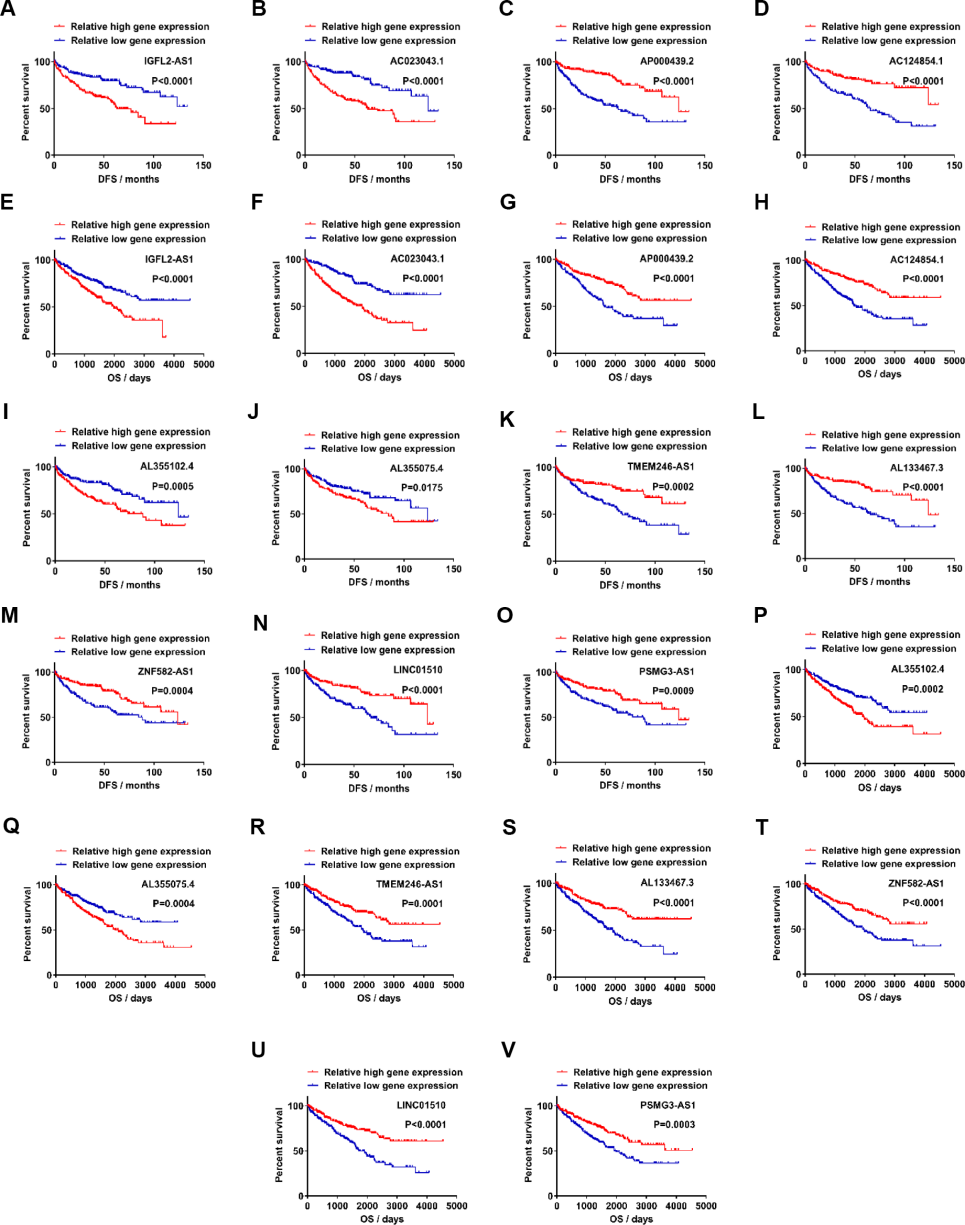
**Candidate lncRNAs acts as potentially prognostic biomarkers for clear cell renal cell carcinoma.** (**A**–**D**) Upregulated lncRNAs exhibited statistically significant difference between relative high gene expression and low expression in DFS. (**E**–**H**) Upregulated lncRNAs exhibited statistical difference in OS. (**I**–**O**) Downregulated lncRNAs exhibited statistical difference in DFS. (**P**–**V**) Downregulated lncRNAs exhibited statistical difference in OS. lncRNAs, long non-coding RNAs.

To determine which lncRNAs were associated with the prognosis of cancer patients, 122 upregulated lncRNAs were analyzed in relation to DFS. Samples from the TCGA-KIRC database (n=356) were divided into two groups, according to candidate lncRNA expression (from high to low; Supplementary Table 3). Out of 122 upregulated lncRNAs, 32 exhibited statistical difference between relative high gene expression and relative low gene expression groups in DFS (Figure 2A–2D, Supplementary Figure 5). All 32 of the upregulated lncRNAs passed the statistical tests in OS analysis between relative high gene expression and low gene expression groups (Figure 2E–2H, Supplementary Table 4, Supplementary Figure 5). Out of the 99 downregulated lncRNAs, 36 were associated with DFS, while 25/36 exhibited statistical difference in OS (Figure 2I–2V, Supplementary Figure 6). To further verify the role of candidate lncRNAs in prognosis, we applied multivariate and univariate Cox regression analysis. Considering that age and gender are risk factors for RCC and other clinicopathological characteristics, such as T, N, M, grade and stage, are classic diagnostic markers, Cox regression analysis was performed for these factors (age, gender, T, N, M, grade, stage and gene expression) [26]. The results showed that 4/32 upregulated and 7/25 downregulated lncRNAs were valuable both in DFS and OS, which indicated that these 11 candidate lncRNAs could potentially be prognostic indicators for kidney cancer. In multivariate Cox regression analysis for DFS, 10/11 candidate lncRNAs, together with N, M, grade and stage, constituted prognostic factors in ccRCC. AC023043.1, AL355102.4 and AL355075.4 were risk factors with a hazard ratio (HR) >1 (Supplementary Tables 7, 13, 15), while AP000439.2, AC124854.1, TMEM246-AS1, AL133467.3, ZNF582-AS1, LINC01510 and PSMG3-AS1 were protective factors with an HR <1 (Supplementary Tables 9, 11, 17, 19, 21, 23, 25). N, M, grade and stage, which were used for primary diagnosis, all exhibited poor prognosis with advanced levels (N1, M1, G3+G4 and S3+S4). IGFL2-AS1, a risk factor in ccRCC, together with M, grade and stage, were identified as prognostic factors (Supplementary Table 5). In multivariate Cox regression analysis for OS, 9/11 candidate lncRNAs, together with age, M, grade and stage, were found to be prognostic factors in ccRCC. Still, IGFL2-AS1, AC023043.1, AL355102.4 and AL355075.4 were identified as risk factors with an HR >1 (Supplementary Tables 6, 8, 14, 16), while AC124854.1, AL133467.3, ZNF582-AS1, LINC01510 and PSMG3-AS1 were identified as protective factors with an HR <1 (Supplementary Tables 12, 20, 22, 24, 26). AP000439.2 and TMEM246-AS1, together with age, N, M, grade and stage, constituted prognostic factors in ccRCC, among which AP000439.2 and TMEM246-AS1 exhibited protective values (Supplementary Tables 10, 18). Age, which was indirectly associated with patient health status, was identified as a risk factor with an HR >1. The reason why age was a prognostic factor for OS but not DFS, may be due to the larger patient population (445 samples) in the OS analysis cohort.

### Assessment of the correlation between candidate lncRNAs and clinicopathological characteristics

As shown in Figures 1C, 1D, 2A, 2B and 2E–2F, and Supplementary Tables 5–8, the IGFL2-AS1 and AC023043.1 genes were upregulated in kidney cancer samples and indicated poor prognosis when highly expressed, which revealed that IGFL2-AS1 and AC023043.1 tended to be risk factors with higher gene expression for Cox HR >1. However, in Figures 1E, 1F, 2C, 2D and 2G–2H, and Supplementary Tables 9–12, the AP000439.2 and AC124854.1 genes were upregulated in kidney cancer samples and indicated good prognosis when highly expressed, which revealed that AP000439.2 and AC124854.1 tended to be protective factors with higher gene expression for a Cox HR <1. Similar phenomena were observed in 7 downregulated candidate lncRNAs. In Figure 1G, 1H, 2I–J and 2P–Q, and Supplementary Tables 13–16, the AL355102.4 and AL355075.4 genes were downregulated in kidney cancer samples and indicated good prognosis when lowly expressed, which revealed that AL355102.4 and AL355075.4 tended to be risk factors with higher gene expression for a Cox HR >1. Nevertheless, in Figures 1I–1M, 2K–2O and 2R–2V, and Supplementary Tables 17–26, the TMEM246-AS1, AL133467.3, ZNF582-AS1, LINC01510 and PSMG3-AS1 genes were downregulated in kidney cancer samples and indicated poor prognosis when lowly expressed, which revealed that TMEM246-AS1, AL133467.3, ZNF582-AS1, LINC01510 and PSMG3-AS1 tended to be protective factors with higher gene expression for a Cox HR <1.

**Figure 3:**
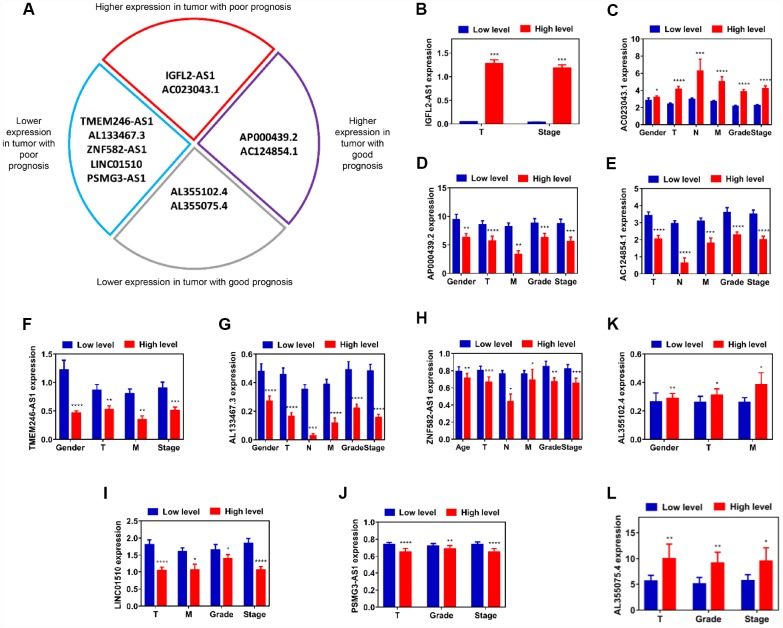
**Assessment of correlation between candidate lncRNAs and clinicopathological characteristics.** (**A**) Schematic diagram of four subgroups from 11 candidate lncRNAs. (**B**–**L**) Relative expression level comparison of each lncRNA in different characteristic subgroups (age, gender, T, N, M, grade and stage). *P<0.05, **P<0.01, ***P<0.001 and ****P<0.0001. lncRNAs, long non-coding RNAs.

Based on the data from this classified analysis, these 11 lncRNAs were divided into four subgroups, as follows: (i) higher expression in tumor with poor prognosis; (ii) higher expression in tumor with good prognosis; (iii) lower expression in tumor with good prognosis; (iv) lower expression in tumor with poor prognosis (Figure 3A). Pearson’s χ^2^ test was performed to determine whether lncRNAs were closely associated with clinicopathological factors of kidney cancer patients (Supplementary Tables 27–37). To visualize and clarify these relationships, we divided patient samples into two subgroups, according to corresponding characteristics (age <=60 vs age >60, female vs male, T1+T2 vs T3+T4, N0 vs N1, M0 vs M1, G1+G2 vs G3+G4, S1+S2 vs S3+S4), which exhibited statistical differences with respect to each lncRNA expression after Pearson’s χ ^2^ test. IGFL2-AS1 gene expression was closely associated with T and stage, which meant that advanced T and stage kidney cancer patients exhibited higher IGFL2-AS1 expression levels (Figure 3B). The AC023043.1 gene expression was closely associated with gender, T, N, M, grade and stage, which revealed that male patients and advanced T, N, M, grade and stage kidney cancer patients exhibited higher AC023043.1 expression levels (Figure 3C). The AP000439.2 gene expression was closely associated with gender, T, M, grade and stage, which meant that male patients and advanced T, M, grade and stage kidney cancer patients exhibited lower AP000439.2 expression levels (Figure 3D). The AC124854.1 gene expression was closely associated with T, N, M, grade and stage, which revealed that advanced T, N, M, grade and stage kidney cancer patients exhibited lower AC124854.1 expression levels (Figure 3E). The TMEM246-AS1 gene expression was closely associated with gender, T, M and stage, which meant that male patients and advanced T, M and stage kidney cancer patients exhibited lower TMEM246-AS1 expression levels (Figure 3F). The AL133467.3 gene expression was closely associated with gender, T, N, M, grade and stage, which revealed that male patients and advanced T, N, M, grade and stage kidney cancer patients exhibited lower AL133467.3 expression levels (Figure 3G). The ZNF582-AS1 gene expression was closely associated with age, T, N, M, grade and stage, which meant that older patients (>60 years) and advanced T, N, M, grade and stage kidney cancer patients exhibited lower ZNF582-AS1 expression levels (Figure 3H). LINC01510 gene expression was closely associated with T, M, grade and stage, which revealed that advanced T, M, grade and stage kidney cancer patients exhibited lower LINC01510 expression levels (Figure 3I). PSMG3-AS1 gene expression was closely associated with T, grade and stage, which meant that advanced T, grade and stage kidney cancer patients exhibited lower PSMG3-AS1 expression levels (Figure 3J). The AL355102.4 gene expression was closely associated with gender, T and M, which revealed that male patients and advanced T and M kidney cancer patients exhibited higher AL355102.4 expression levels (Figure 3K). The AL355075.4 gene expression was closely associated with T, grade and stage, which meant that advanced T, grade and stage kidney cancer patients exhibited higher AL355075.4 expression levels (Figure 3L). In a word, gene expression differences between normal and tumor samples have no relation to prognosis for survival rates. Impact factors are relative gene expression differences between advanced stages and early stages in tumor samples. That is, in tumor samples with advanced T, N, M, grade, stage and relatively older age, lncRNAs in higher expression levels indicated poor prognosis for ccRCC patients.

### LncRNAs play diagnostic roles in ccRCC

**Figure 4:**
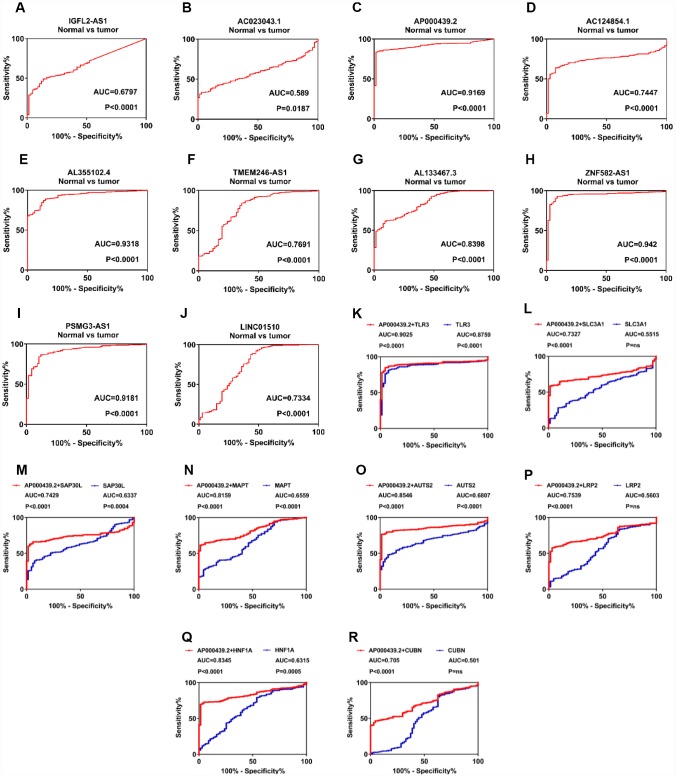
**LncRNAs play a diagnostic role in patients with clear cell renal cell carcinoma.** (**A**–**J**) ROC curve analysis was performed to differentiate kidney cancer patients from healthy individuals, according to lncRNA expression. (**K**–**R**) Diagnostic accuracy was improved after combing AP000439.2 with previously reported RCC biomarkers. lncRNAs, long non-coding RNAs; ROC, receiver operating characteristic curve; RCC, renal cell carcinoma.

To further investigate the diagnostic values of 11 candidate lncRNAs in ccRCC, ROC analysis was performed to differentiate kidney cancer patients from healthy individuals, according to lncRNA expression. Out of 11 genes, 10 (all but AL355075.4) exhibited good diagnostic values (AUC>0.5 and P<0.05; Figure 4A–4J). Among these, AP000439.2 (AUC=0.9169, P<0.0001), AL355102.4 (AUC=0.9318, P<0.0001), ZNF582-AS1 (AUC=0.942, P<0.0001) and PSMG3-AS1 (AUC=0.9181, P<0.0001) exhibited the strongest diagnostic values. To obtain more detailed information about the 10 candidate lncRNAs, DFS and OS analysis of the ccRCC subgroups was performed in relation to clinicopathological characteristics such as G1+G2, M0, N0, T1+T2 and S1+S2. In addition, ROC curves were used to distinguish between the G1+G2 and G3+G4, M0 and M1, N0 and N1, T1+T2 and T3+T4, and S1+S2 and S3+S4 patient samples (Supplementary Figure 7–16). Two lncRNAs, AC023043.1 and AL133467.3, exhibited statistically significant differences for both DFS and OS in the G1+G2, M0, N0, T1+T2 and S1+S2 subgroups. Furthermore, AC023043.1 and AL133467.3 could also distinguish between the G1+G2 and G3+G4, M0 and M1, N0 and N1, T1+T2 and T3+T4, and S1+S2 and S3+S4 kidney cancer patient subgroups (Supplementary Figure 8, 13). Overall, 10 candidate lncRNAs (IGFL2-AS1, AC023043.1, AP000439.2, AC124854.1, AL355102.4, TMEM246-AS1, AL133467.3, ZNF582-AS1, LINC01510 and PSMG3-AS1) were selected as diagnostic and prognostic biomarkers for ccRCC, as they exhibited a strong correlation with clinicopathological characteristics.

To further explore additional values when combining identified lncRNAs with known RCC biomarkers, we have performed ROC analysis for multi-gene diagnosis. Considering highest diagnostic value of AP000439.2 among four upregulated lncRNAs, 11 previously reported RCC biomarkers were selected and undertaken multivariate COX analysis with AP000439.2 (Supplementary Figure 17A). As shown in Figure 4K–4R, compared with single-gene diagnosis, AP000439.2 significantly improved diagnostic value of 8 reported RCC biomarkers, which indicated strong diagnostic efficacy of AP000439.2. However, no improvement of diagnostic accuracy was found when combining AP000439.2 with AQP1, HAO2 and PDZK1 respectively (Supplementary Figure 17B–17D).

### Potential functional roles of 10 candidate lncRNAs in ccRCC tumorigenesis

**Figure 5:**
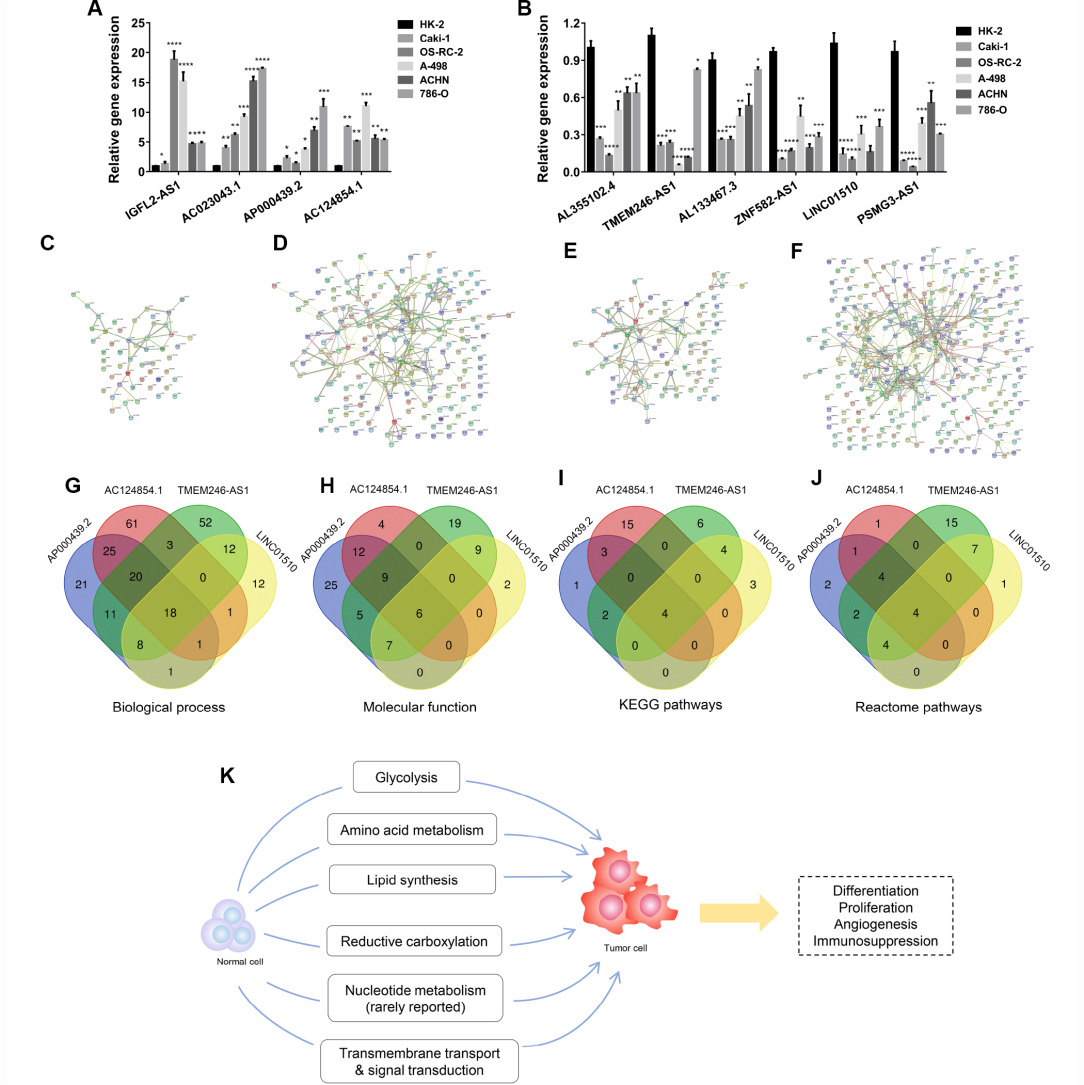
**Potentially functional roles of candidate lncRNAs participating in ccRCC tumorigenesis.** (**A**–**B**) Relative expression of lncRNAs was verified in cell lines by RT-qPCR. (**C**–**F**) Four modules of protein-protein interaction networks. (**G**–**J**) Intersections of these modules with respect to biological process, molecular function, and KEGG and Reactome pathways. (**K**) Potential mechanistic schematic diagram of the role of candidate lncRNAs in kidney cancer tumorigenesis. Each experiment was performed at least three times and data are presented as the mean ± SEM. P<0.05 was considered to indicate a statistically significant difference. *P<0.05, **P<0.01, ***P<0.001 and ****P<0.0001. lncRNAs, long non-coding RNA.

Apart from diagnostic and prognostic values in the clinic, we wanted to further explore which mechanisms these lncRNAs might participate in during ccRCC tumorigenesis. First, the lncRNA relative expression levels was verified in kidney cancer cell lines and 12 paired tumor and normal tissues (Figure 5A, 5B, Supplementary Figure 18A–18D, Supplementary Figure 19A–19F). As shown by the results, IGFL2-AS1, AC023043.1, AP000439.2 and AC124854.1 were upregulated in tumor cells and tissues, while AL355102.4, TMEM246-AS1, AL133467.3, ZNF582-AS1, LINC01510 and PSMG3-AS1 were downregulated in KIRC cell lines and tumors. Protein-protein interactions (PPI) network was constructed from STRING database and biological process, molecular function, KEGG pathways and Reactome pathways were presented. Four modules of the PPI networks were visualized. The corresponding lncRNAs were LINC01510 (Figure 5C), AP000439.2 (Figure 5D), TMEM246-AS1 (Figure 5E) and AC124854.1 (Figure 5F), respectively. Intersections of enrichment pathways were conducted with respect to biological process (Figure 5G), molecular function (Figure 5H), KEGG pathways (Figure 5I) and Reactome pathways (Figure 5J). Each kind of functional pathway is summarized and listed in the tables, if it was mapped to three or four lncRNAs (Supplementary Tables 38–41). Most functional pathways were enriched in cellular metabolic processes, such as glycolysis, amino acid metabolism, lipid synthesis, reductive carboxylation, nucleotide metabolism, transmembrane transport and signal transduction. To verify these, GSEA was also performed using the TCGA-KIRC database for each lncRNA. IGFL2-AS1 was enriched in cytokine-cytokine receptor interaction, the p53 and JAK-STAT signaling pathways (Supplementary Figure 20A), while AC023043.1 possibly participated in α-linolenic acid metabolism and the cytosolic DNA sensing pathway (Supplementary Figure 20B). AP000439.2 was found to involve renal cell carcinoma, fatty acid, tryptophan, histidine and glycerolipid metabolism, and the mTOR signaling pathway (Supplementary Figure 20C), while AC124854.1 might participate in the adipocytokine signaling pathway, renal cell carcinoma, histidine metabolism, mTOR signaling pathway and glycolysis gluconeogenesis (Supplementary Figure 21A). AL355102.4 was enriched in histidine, tryptophan, beta alanine, fatty acid and arginine-proline metabolism (Supplementary Figure 21B), while ZNF582-AS1 possibly participated in pentose and glucuronate interconversions, the pentose phosphate pathway, alanine aspartate-glutamate metabolism and the p53 signaling pathway (Supplementary Figure 22A). LINC01510 was involved in the p53 and Wnt signaling pathways, and cell cycle (Supplementary Figure 22B). Results from GSEA analysis were consistent with PPI enrichment pathways. These indicated that ccRCC progression was closely associated with metabolic mechanisms, including glycolysis, amino acid metabolism, lipid synthesis, reductive carboxylation, nucleotide metabolism, transmembrane transport and signal transduction, which were further associated with tumor cell proliferation, differentiation, angiogenesis and immunosuppression [26, 27] (Figure 5K).

### Inner mechanisms of dysregulated lncRNAs

LncRNAs play crucial roles in various cellular and physiological processes and exert functional effects on tumor progression. Dysregulated expression patterns of lncRNAs in cancer development indicate that these lncRNAs may act as potential molecular biomarkers. The inner mechanisms of lncRNA dysregulation would be categorized as somatic copy-number alterations (SCNAs) amplification, chromosome loci deletion, transcription factor binding, histone acetylation, histone methylation and DNA methylation [28–33]. Hence, we attempted to make histone acetylation and histone methylation prediction of 10 candidate lncRNAs. As shown in Supplementary Figure 23A–23J, high enrichment of H3K27Ac, H3K4Me1 and H3K4Me3 were found at the promoter regions of AC023043.1, AL133467.3, PSMG3-AS1, ZNF582-AS1 and AL355102.4. These indicated that histone acetylation and methylation activation at promoter regions may partly account for dysregulation of above 5 lncRNAs in RCC. Considering some dysregulation might occur at transcriptional level, we wanted to find these transcriptional regulators altered in the kidney cancer and figured out their clinical values. Transcriptional regulators of each candidate lncRNA were screened and selected from at least 3 gene sets. Relative gene expression levels of 21 transcriptional factors were compared between 72 normal and tumor samples using paired student's *t*-test. Out of the 16 transcriptional factors, 8 were associated with OS, while 7/8 exhibited statistical difference for DFS (Supplementary Figure 24A). Among these 7 genes, CTBP1 was upregulated in kidney cancer samples and indicated poor prognosis when highly expressed, which revealed that CTBP1 tended to be a risk factor with higher gene expression (Supplementary Figure 24C). ARNT, CTCF, POLR2A, RAD21 and REST were downregulated in kidney cancer samples and indicated poor prognosis when lowly expressed, which revealed that ARNT, CTCF, POLR2A, RAD21 and REST tended to be risk factors with lower gene expression (Supplementary Figure 24B, 24D, 24E, 24F, 24H). SP1 was upregulated in kidney cancer samples and indicated good prognosis when highly expressed, which revealed that SP1 tended to be a protective factor with higher gene expression (Supplementary Figure 24G).

## DISCUSSION

Following complete surgical resection, 20-40% of ccRCC patients were at risk of postoperative metastasis or local recurrence [34]. Traditional T, N, M, grade and stage are not sufficient to distinguish early-stage and indolent tumors, therefore requiring the assistance of methods such as preoperative biopsy or imaging. This limitation renders the discovery of novel prognostic and diagnostic biomarkers crucial to early intervention in ccRCC. An increasing body of evidence has revealed that lncRNAs could preliminarily identify tumor progression and predict the outcome prior to or following treatment, which indicated that lncRNAs might be biomarkers for clinical diagnosis and prognosis, especially in ccRCC [16, 19, 23, 35–44].

In the present study, we identified 10 candidate lncRNAs, which exhibited good diagnostic and prognostic value in ccRCC. Hierarchical clustering heat map analysis was performed for differentially expressed lncRNAs. Similar methods have been used, but the application of unpaired normal and tumor samples caused population-related statistical bias [16, 19, 20, 24, 25]. Herein, 54 paired samples were included in the cohort and underwent differential expression analysis using R package ‘edgeR’. In addition, paired student's *t*-test was performed to compare 54 paired normal and tumor samples, one by one, to eliminate errors in the R package output. Three upregulated lncRNAs exhibited no statistically significant difference in gene expression of 54 paired samples. DFS and OS survival analysis was also performed for each selected lncRNA, to ensure that the candidate lncRNAs were closely associated with the prognosis of cancer patients. Following this process, only 1/4 of the lncRNAs were selected for the next step. Multivariate and univariate Cox regression analysis was applied to identify interactions between gene expression and several clinicopathological parameters. Multivariate Cox regression analysis revealed that N, M, grade and stage were risk factors for KIRC. Of note, the T classification did not act as a significant prognostic factor. This may be due to the fact that the T classification reflects tumor size, which cannot determine the metastatic status of ccRCC. However, the N and M classifications were closely associated with cancer metastasis, and were therefore of great prognostic importance. Eleven candidate lncRNAs were found to have an effect on KIRC prognosis. In order to investigate the diagnostic value of 11 candidate lncRNAs in ccRCC, ROC analysis was performed to differentiate kidney cancer patients from healthy individuals, according to lncRNA expression. LncRNA-AL355075.4 exhibited poor diagnostic values (AUC=0.5013, P=0.9726), and was not considered. Finally, to explore the inner mechanisms associated with candidate lncRNAs during ccRCC progression, we made correlation analysis of the 10 candidate lncRNAs with each gene in the TCGA-KIRC database (60,483 genes) one by one. LncRNAs have no protein coding potential, which means that they need to exert regulatory functions, assisted by mRNA [45]. A coefficient of >0.5 was set as the cut-off value and mapping genes in the TCGA-KIRC database were used to construct PPI networks, which could reflect the potentially regulatory functions of lncRNAs. Certain studies intended to build competing endogenous RNA networks to link corresponding mRNA to relevant lncRNAs [16, 19, 42]. However, this construction largely depended on databases such as miRcode, miRTarBase, miRDB and TargetScan. Existing interactions between lncRNA and mRNA towards specific tumor types are recorded in these databases, while unexplored or not reported connections between them are not included. We therefore chose the correlation coefficient method to find mRNAs or proteins corresponding to candidate lncRNAs. In addition, GSEA analysis was performed for each lncRNA, and KEGG signaling pathways were matched to enrichment processes derived from PPI networks.

Among the 10 candidate lncRNAs, AC023043.1, AC124854.1, AL355102.4, TMEM246-AS1, AL133467.3 and PSMG3-AS1 had not been previously reported. IGFL2-AS1 was found to be involved in breast cancer progression following transcriptome wide sequencing analysis [46]. AP000439.2 was found to be associated with non-small cell lung cancer by pathway analysis method based on global influence (PAGI) [47]. ZNF582-AS1 was reported to be methylated in colorectal cancer cells, and this methylation was closely associated with colorectal tumorigenesis [48]. LINC01510 was also upregulated in colorectal cancer tissues, and the higher LINC01510 expression predicted poor prognosis with respect to DFS and OS [49, 50]. In addition, LINC01510 was also associated with poor prognosis and promoted malignant progression in non-small cell lung cancer [51]. One study verified that LINC01510 was downregulated in RCC and suppressed cell proliferation by inhibiting Wnt/β-catenin signaling [52]. In the ‘Results’ section, we discussed that gene expression differences between normal and tumor samples have no relation to prognosis for survival rates. Impact factors are relative gene expression differences between advanced stages and early stages in tumor samples. That is, in tumor samples with advanced T, N, M, grade, stage and relatively older age, lncRNAs in higher expression levels indicated poor prognosis for ccRCC patients.

However, another question raised our interests. Upregulated lncRNAs in tumor samples exhibited relatively lower expression levels in advanced stages (such as AP000439.2 and AC124854.1, Figure 1E, 1F, Figure 3D, 3E). Downregulated lncRNAs in cancer tissues exhibited relatively higher expression levels in advanced stages (such as AL355102.4, Figure 1G, Figure 3K). Relevant studies have been conducted in nine morphological stages of lung squamous cell carcinoma. Four distinct and successive biological processes were discovered and molecular pathways were involved in specific steps of tumorigenesis [53]. A previous study has also reported that miR-34c might be upregulated in lung formation, but was inversely downregulated during bronchial tumorigenesis [54]. These results have indicated that carcinogenesis is closely associated with consecutive developmental stages. Inner mechanisms underlying the early evolution among different cancer stages might be different or even inverse. That is why the expression levels of lncRNAs might differ between early and advanced stages.

Enrichment pathways have indicated that, during ccRCC carcinogenesis, changes in metabolic levels mainly contribute to the formation of tumor cells. Several studies have revealed that RCC was closely associated with glycolysis [55–62]. In normal cells, pyruvate mainly comes from glucose under normoxia. However, cancer cells produce energy primarily by glycolysis followed by lactate fermentation, rather than by glycolysis followed by TCA cycle in mitochondria, regardless of the oxygen level [27]. This phenomenon is especially common in ccRCC [63]. Some studies have reported that increased expression levels of glucose transporter 1 (GLUT-1) were correlated with decreased numbers of infiltrating CD8^+^ T cells, which indicated that increased lactate levels caused by GLUT-1 induction might inhibit T cell activities [64, 65]. Furthermore, HIF-1α increased the expression level of GLUT-1, which promoted cellular glucose uptake in VHL-deficient ccRCC and further induced angiogenesis [66]. Amino acid metabolism has also been reported to participate in ccRCC tumorigenesis [67–69]. Tryptophan was increasingly used in ccRCC cells, which enhanced tumor growth and further resulted in immunosuppression with low efficiency of interferon-α immunotherapy [70]. Arginine played a vital role during ccRCC progression and is largely depended on activities of argininosuccinate synthase 1 (ASS1) [71]. ASS1 was downregulated in kidney cancer cells, but upregulated in normal proximal tubule cells, which made cells rely on extracellular sources of arginine for survival [72]. Abnormal lipid metabolism in ccRCC was reported early in 1987, in a study that identified increased cholesterol ester in the tumor tissues, as compared with normal kidney tissues [73]. Other studies have found that increased levels of Stearoyl-CoA desaturase 1 and fatty acid synthase in KIRC tissues are required for tumor growth and progression [74, 75]. In addition, a recent study has found that phospholipase C-like 1/uncoupling protein 1 mediating lipid browning could promote kidney cancer cell “slimming” and consume abnormal lipid accumulation, which represses the progression of ccRCC [76]. Reductive carboxylation was also involved in the progression of RCC [58, 77–80]. It was reported that glutamine was utilized in the reductive carboxylation pathway, resulting in increased levels of citrate and cis-aconitate, which further promoted fatty acid synthesis [81, 82]. Nucleotide metabolism, however, has rarely been studied to date. Most researches concentrated on predicted pathways of profile sequencing [83–85]. Despite the altered energy and glutamine metabolism, or altered arginine metabolism, these mentioned events happen in the mitochondria, which are related to transmembrane transport and signal transduction [27]. We therefore summarized 6 metabolic and functional pathways which may be closely associated with kidney cancer tumorigenesis: glycolysis, amino acid metabolism, lipid synthesis, reductive carboxylation, nucleotide metabolism, transmembrane transport and signal transduction.

In conclusion, 10 candidate lncRNAs, which exhibited diagnostic and prognostic values in ccRCC patients, were identified in the present study. Further studies focusing on the detailed functional roles of these lncRNAs should be conducted and more information needed to be provided during ccRCC tumorigenesis.

## MATERIALS AND METHODS

### Cell culture

The HK-2 human renal proximal tubular epithelial cell line and the 786-O, OS-RC-2, ACHN, A-498 and Caki-1 human RCC cell lines were purchased from the American Type Culture Collection (Manassas, VA, USA). Cells were maintained at 37°C in a 5% CO_2_ incubator and cultured in high glucose Dulbecco's modified Eagle's medium (Thermo Fisher Scientific, Inc., Waltham, MA, USA), containing 10% fetal bovine serum (Thermo Fisher Scientific, Inc.) and 1% penicillin-streptomycin.

### Clinical sample preparation

Twelve paired kidney cancer and adjacent normal tissues were collected between 2016 and 2018 at the Department of Urology, Union Hospital (Wuhan, China). The patients whose tissues were used in the present study had never received chemotherapy or radiotherapy. The specimens were stored at −80°C until use. The study protocol was approved by the ethics committee of the Union Hospital and Huazhong University of Science and Technology, and all patients provided written informed consent. The study methodologies conformed to the standards set by the Declaration of Helsinki.

### RT-qPCR

Total RNA was extracted using TRIzol reagent (Thermo Fisher Scientific, Inc.). lncRNAs were reverse-transcribed to cDNA using a PrimeScript RT reagent Kit (Takara Biotechnology Co., Ltd., Dalian, China). RT-qPCR was performed using the ABI StepOnePlus system (Thermo Fisher Scientific, Inc.). Relative expression was calculated using the −2^ΔΔCt^ method, and GAPDH was used as the internal control. The PCR primers used were as follows: AP000439.2, TACTGGGCTAGGCGTCAGAT forward and GATGGCCCAGATACATCGCA reverse; AC124854.1, ACGAAGCCAGCAAAGCAAC forward and GCCCAAACACTTAAACGCCA reverse; IGFL2-AS1, TGACCTATGGACTCAGGAGCA forward and CCTTGGGGCTGAAATCAGGA reverse; AC023043.1, CCGACCGGACTTTCACTTCTG forward and TCTGG AAACATCTTCGTGGGA reverse; AL355102.4, TACTGGGCTAGGCGTCAGAT forward and GATGGC CCAGATACATCGCA reverse; TMEM246-AS1, TGCA CAAGAGGCCAAGTCAA forward and GGTCAGCTC ATAGGTGCCAG reverse; AL133467.3, CCAAGGTCA CACAGCCAAGT forward and CATGAAACTGGCC TCACCAC reverse; ZNF582-AS1, ATGATTGGGAG TCCGAGTGC forward and TCCAGTCCTCACTCAAG GACA reverse; LINC01510, GCAAGAGAAAGACTTG GGGTG forward and TTCCTGTTCCCACTGGCTTG reverse; PSMG3-AS1, TGCCTCGGATTCTGTCCAAC forward and AGGTGAGAAACACCGAGCTG reverse; GAPDH, AAAAGCATCACCCGGAGGAGAA forward and AAGGAAATGAATGGGCAGCCG reverse.

### Differentially expressed gene exploration

The lncRNA expression pattern and clinicopathological characteristics were downloaded from the TANRIC (https://ibl.mdanderson.org/tanric/_design/basic/main.html) and The Cancer Genome Atlas Kidney Clear Cell Carcinoma (TCGA-KIRC; https://xenabrowser.net/heatmap/) databases, respectively. Pairs of tumor and adjacent normal samples were compared using R 3.6.0 software with the edgeR package. Annotations for candidate lncRNAs were retrieved from GeneCards (https://www.genecards.org/) and Ensembl genome browser 90 (http://asia.ensembl.org/index.html). The filtering value was set at |log_2_ fold change (FC)|>1, adjusted P<0.05 and false discovery rate (FDR)<0.01.

### Correlation and protein-protein interactions (PPI) network analysis

Correlation analysis of lncRNAs expression in kidney cancer samples was conducted on Excel 2016 using the CORREL function. Correlation analysis of the 10 candidate lncRNAs was made with each gene in the TCGA-KIRC database (60,483 genes) one by one. After ranking the correlation coefficients from high to low, mapping genes (MGs) of each candidate lncRNA with a correlation coefficient >0.5 were selected. Next, MGs of each lncRNA were put into the STRING database (https://string-db.org/), which automatically constructed PPI networks and presented biological process, molecular function, KEGG pathways and Reactome pathways. P-value <0.05 was considered statistically significant. Venn diagrams were created using Draw Venn Diagram (http://bioinformatics.psb.ugent.be/webtools/Venn/) and jvenn (http://jvenn.toulouse.inra.fr/app/example.html).

### Gene set enrichment analysis (GSEA)

GSEA (http://www.broadinstitute.org/gsea) was performed on KEGG gene sets collected from the TCGA-KIRC database. Gene expression profiles were ranked from high to low and divided into two groups, according to candidate lncRNA expression level. For enriched gene sets, FDR<25% and nominal P<0.05 were considered to be statistically significant.

### Histone acetylation, histone methylation and transcriptional regulators screening

Histone acetylation, histone methylation and transcriptional regulators of candidate lncRNAs was screened on UCSC Genome Browser (GRCh38/hg38). Histone H3 on lysine 27 acetylation (H3K27Ac), histone H3 on lysine 4 monomethylation (H3K4Me1), histone H3 on lysine 4 trimethylation (H3K4Me3) and transcriptional regulators at promoter regions were annotated respectively. Gene expression differences of transcriptional regulators were analyzed in 72 paired tissues. Sample information was downloaded from TCGA KIRC database.

### Selection of previously reported RCC biomarkers

Mapping genes of four upregulated lncRNAs were retrieved from TCGA-KIRC database and 158 genes were found both in AP000439.2 and AC124854.1 gene sets. 14 protein coding genes out of 158 were previously reported as RCC biomarkers and 11/14 exhibited statistical difference together with AP000439.2 after multivariate COX analysis. Risk score was calculated as exp_gene1_ * β_gene1_ + exp_gene2_ * β_gene2_ (exp, expression level, β, the regression coefficient derived from the multivariate Cox regression model). ROC analysis for multi-gene diagnosis was conducted based on risk score of each sample.

### Statistics

Statistical analysis was carried out using Graphpad Prism 7.0 software, and each experiment was performed at least three times. The Student's *t*-test was used to analyze differences in the gene expression between paired samples. Kaplan-Meier curves with log-rank tests were used to assess the correlation between the gene expression and disease-free survival (DFS) and OS rates. The association between gene expression and clinicopathological parameters in RCC was evaluated using Pearson’s χ^2^ test. Receiver operating characteristic curve (ROC) and areas under the curve (AUC) were generated to evaluate the diagnostic values of candidate genes in ccRCC. Multivariate and univariate Cox regression analysis was carried out by SPSS 22.0 software (IBM Corp., Armonk, NY, USA). Data are presented as the mean ± SEM, and P<0.05 was considered to indicate a statistically significant difference.

## Supplementary Material

Supplementary Figures 1-24

Supplementary Table 1

Supplementary Table 2

Supplementary Table 3

Supplementary Table 4

Supplementary Tables 5-41
