# Supplementary material for: A cluster of long non-coding RNAs exhibit diagnostic and prognostic values in renal cell carcinoma
Source: Aging (Albany NY). 2019 Nov 14;11(21):9597–615. doi: 10.18632/aging.102407 (PMC6874440; doi:10.18632/aging.102407)
Supplement: Supplementary Figures 1-24 [file aging-11-102407-s006.pdf]

SUPPLEMENTARY FIGURES

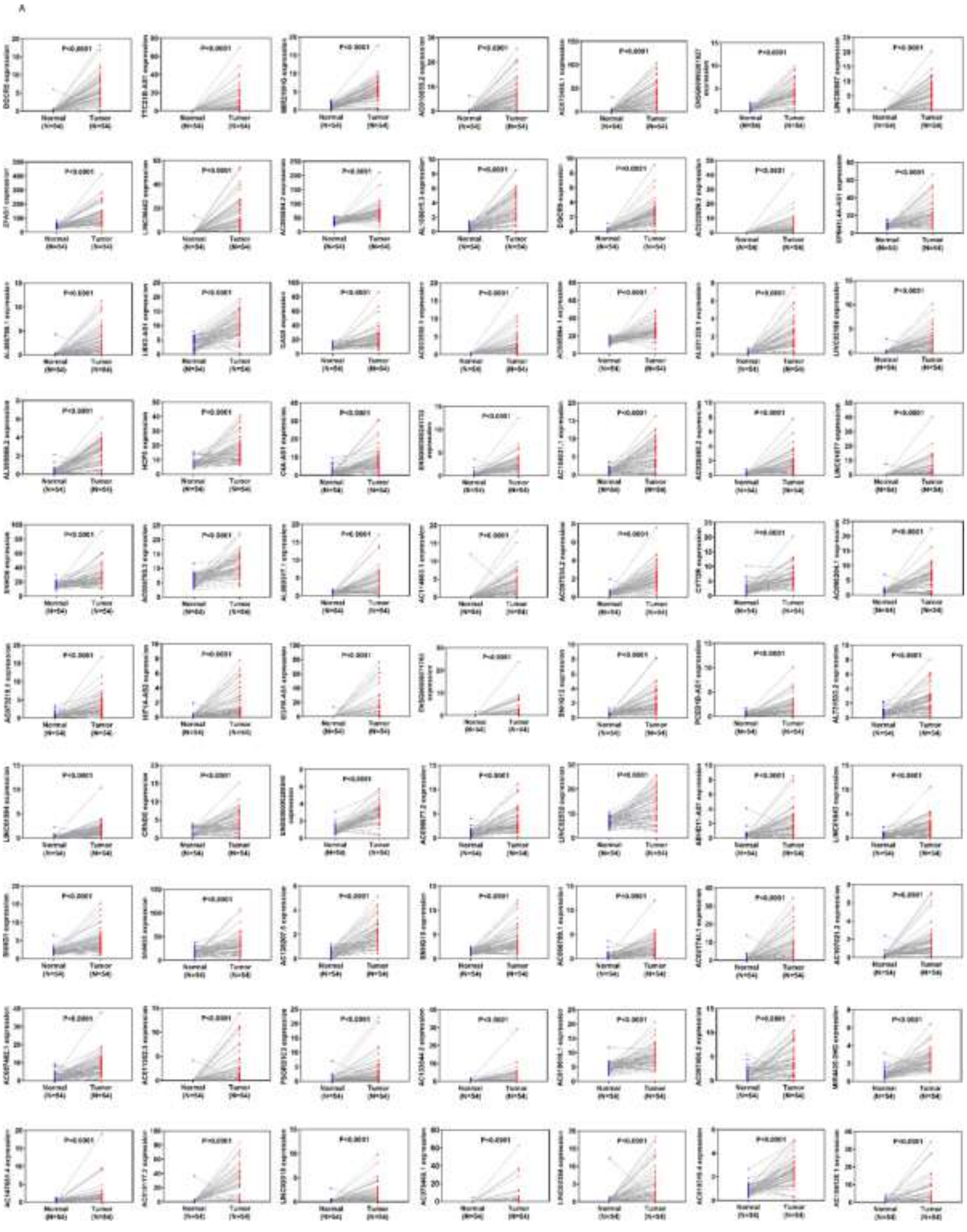

Supplementary Figure 1. Paired tumour and adjacent normal tissues of upregulated lncRNAs with student's *t*-test. 70 upregulated lncRNAs of 54 paired normal and tumor samples exhibited statistical difference after student's *t*-test.

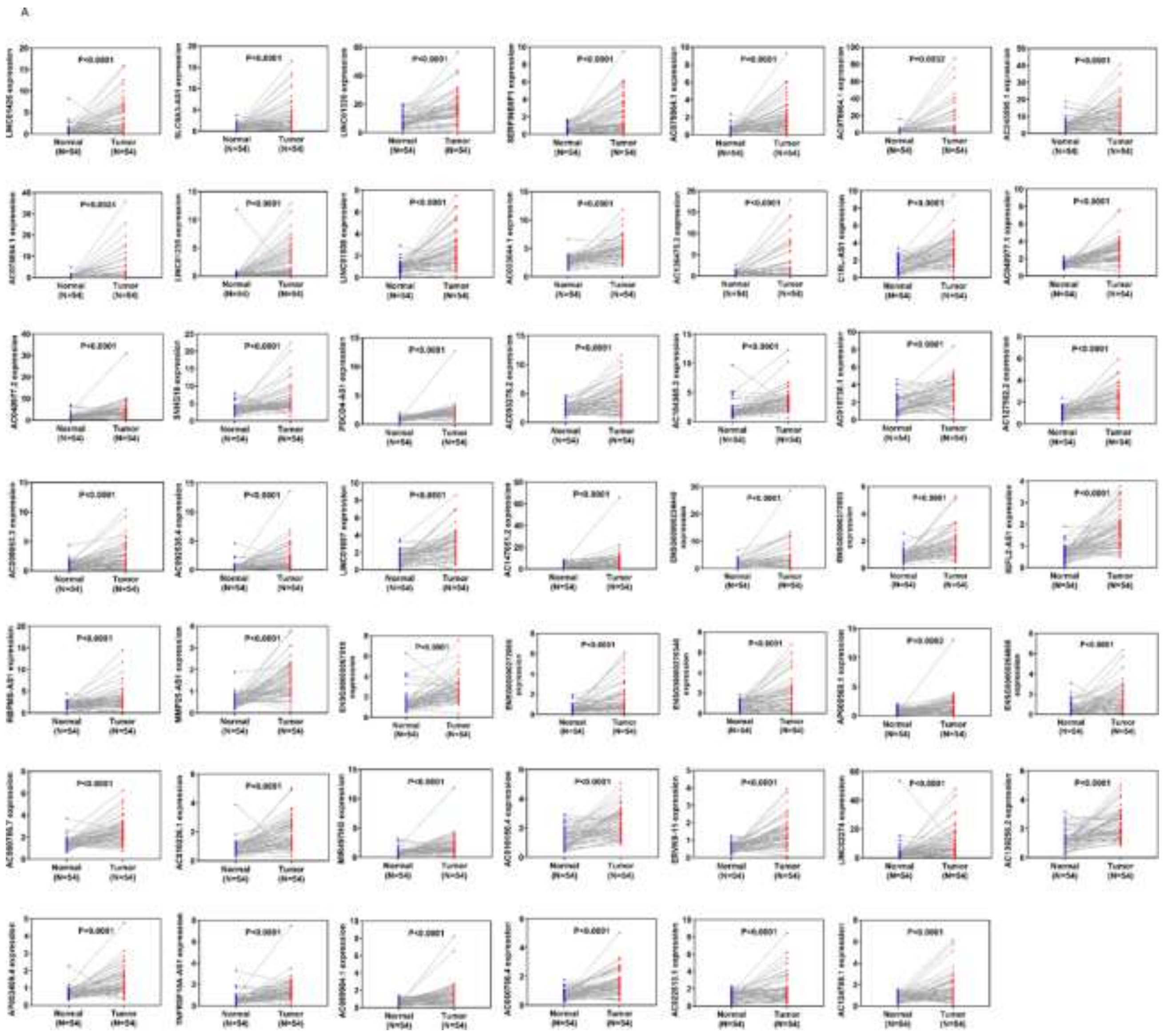

Supplementary Figure 2. Paired tumour and adjacent normal tissues of upregulated lncRNAs with student's *t*-test (continued). 48 upregulated lncRNAs of 54 paired normal and tumor samples exhibited statistical difference after student's *t*-test.

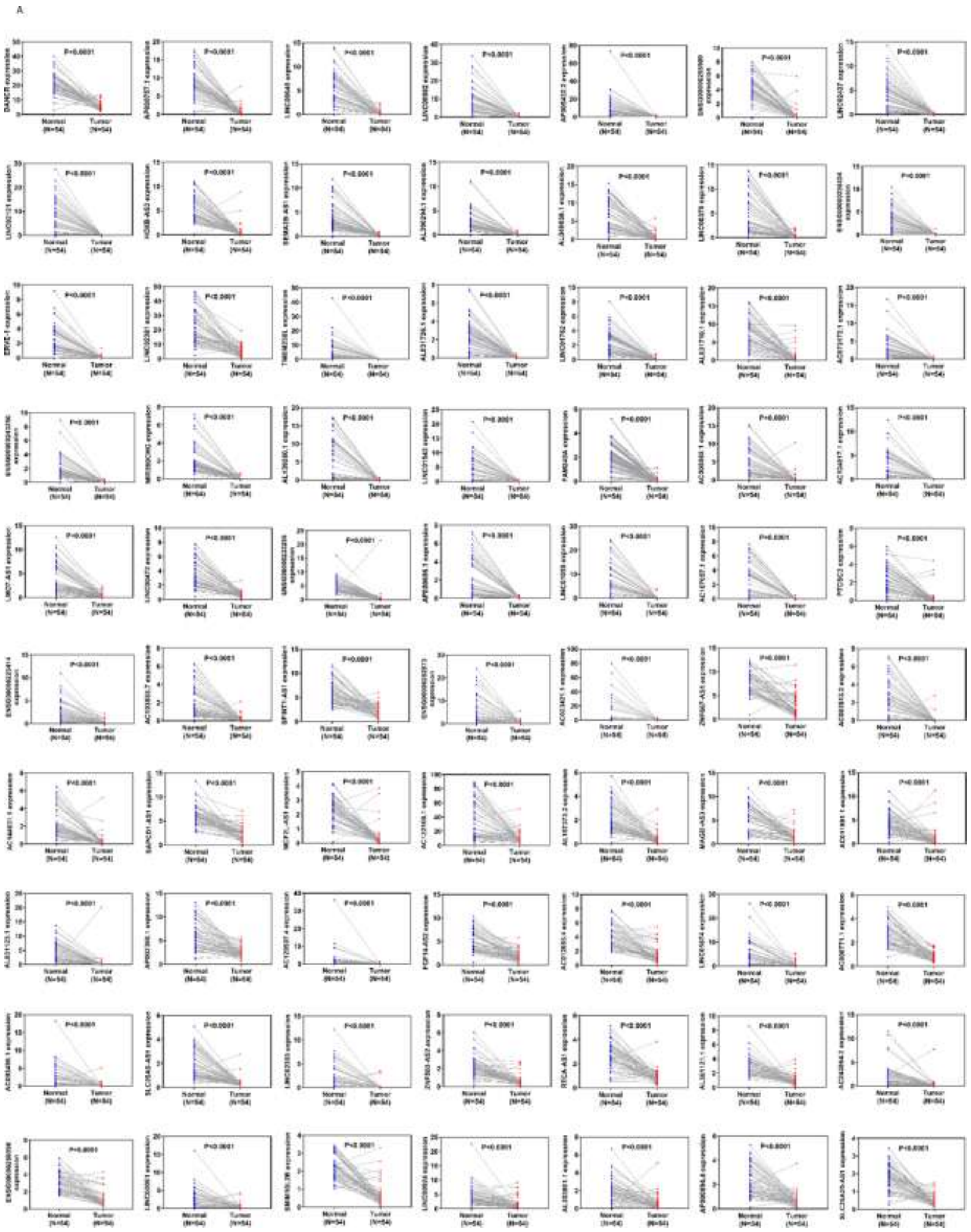

Supplementary Figure 3. Paired tumour and adjacent normal tissues of downregulated lncRNAs with student's *t*-test. 70 downregulated lncRNAs of 54 paired normal and tumor samples exhibited statistical difference after student's *t*-test.

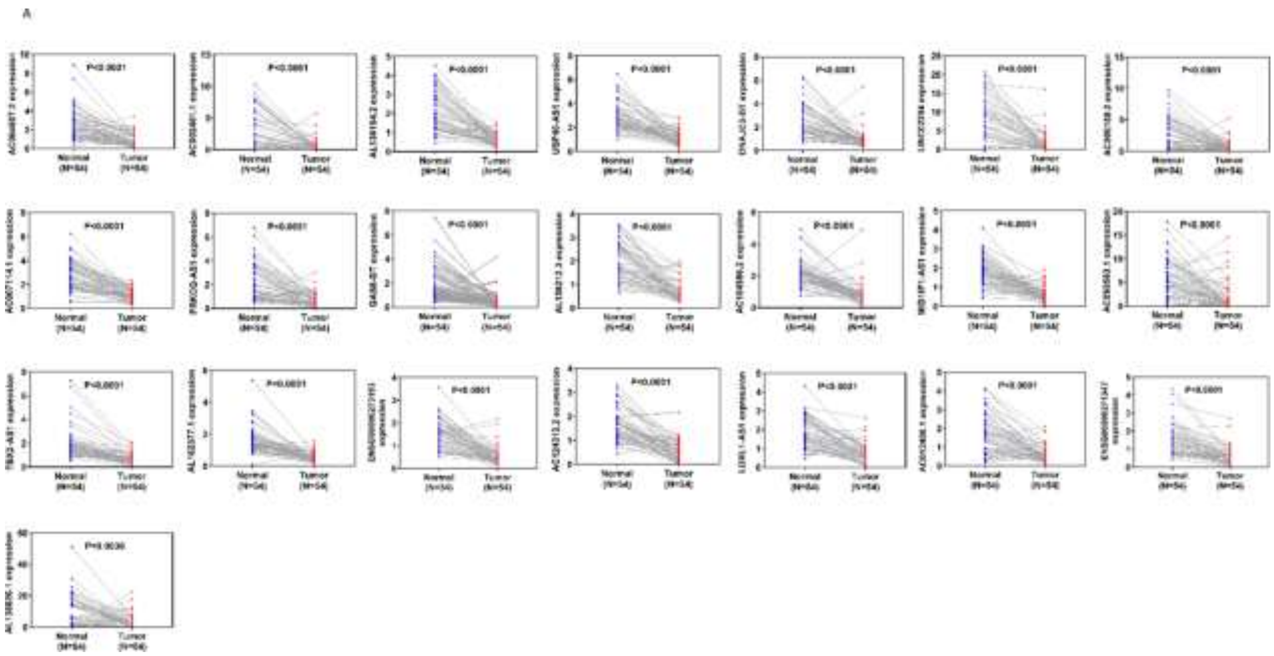

Supplementary Figure 4. Paired tumour and adjacent normal tissues of downregulated lncRNAs with student's *t*-test (continued). 22 downregulated lncRNAs of 54 paired normal and tumor samples exhibited statistical difference after student's *t*-test.

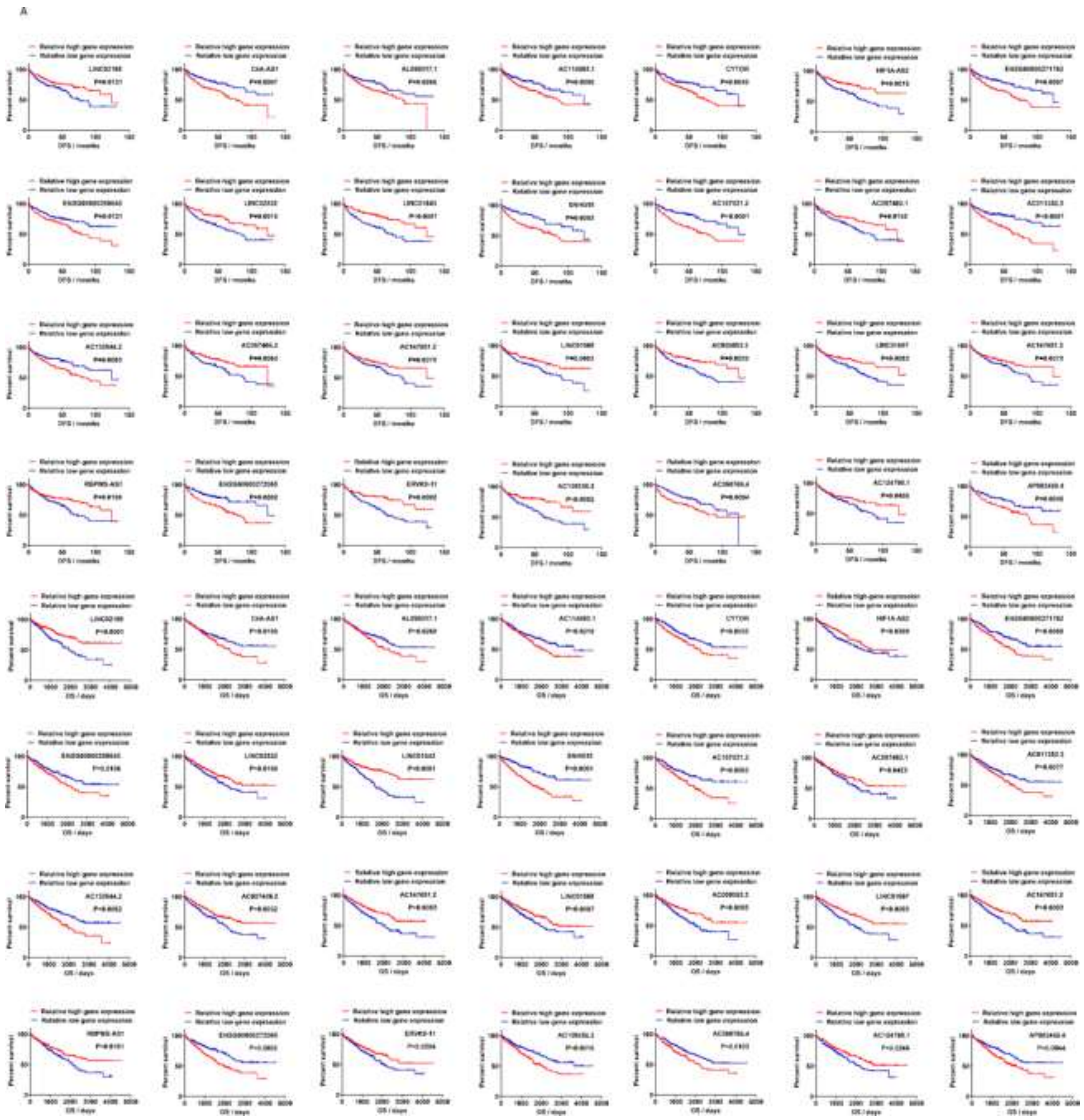

Supplementary Figure 5. Candidate upregulated lncRNAs acts as potentially prognostic biomarkers for clear cell renal cell carcinoma. 28 upregulated lncRNAs exhibited statistically significant difference between relative high gene expression and low gene expression regarding DFS and OS.

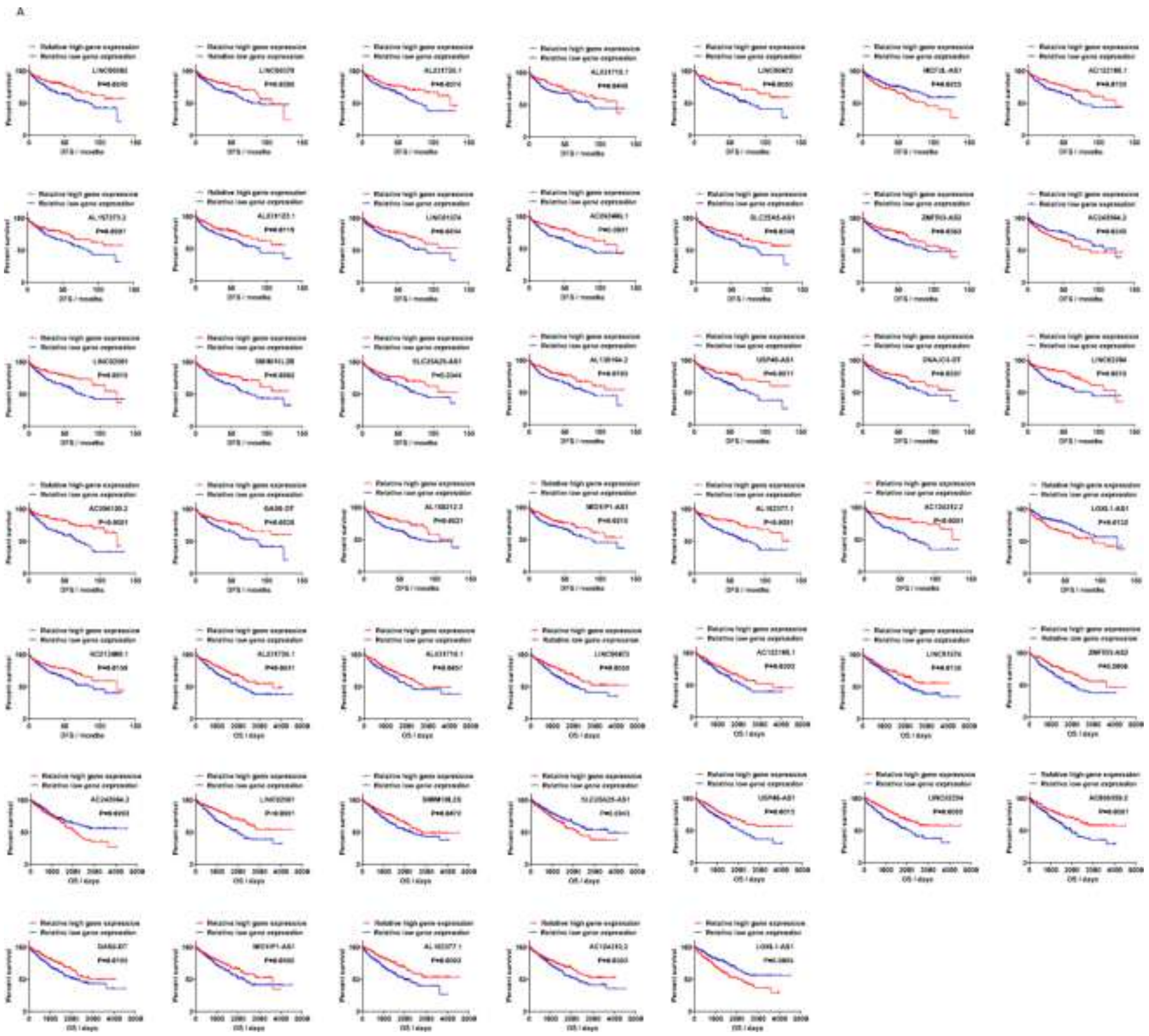

Supplementary Figure 6. Candidate downregulated lncRNAs acts as potentially prognostic biomarkers for clear cell renal cell carcinoma. Downregulated lncRNAs exhibited statistically significant difference between relative high gene expression and low gene expression regarding DFS (n=29) and OS (n=18).

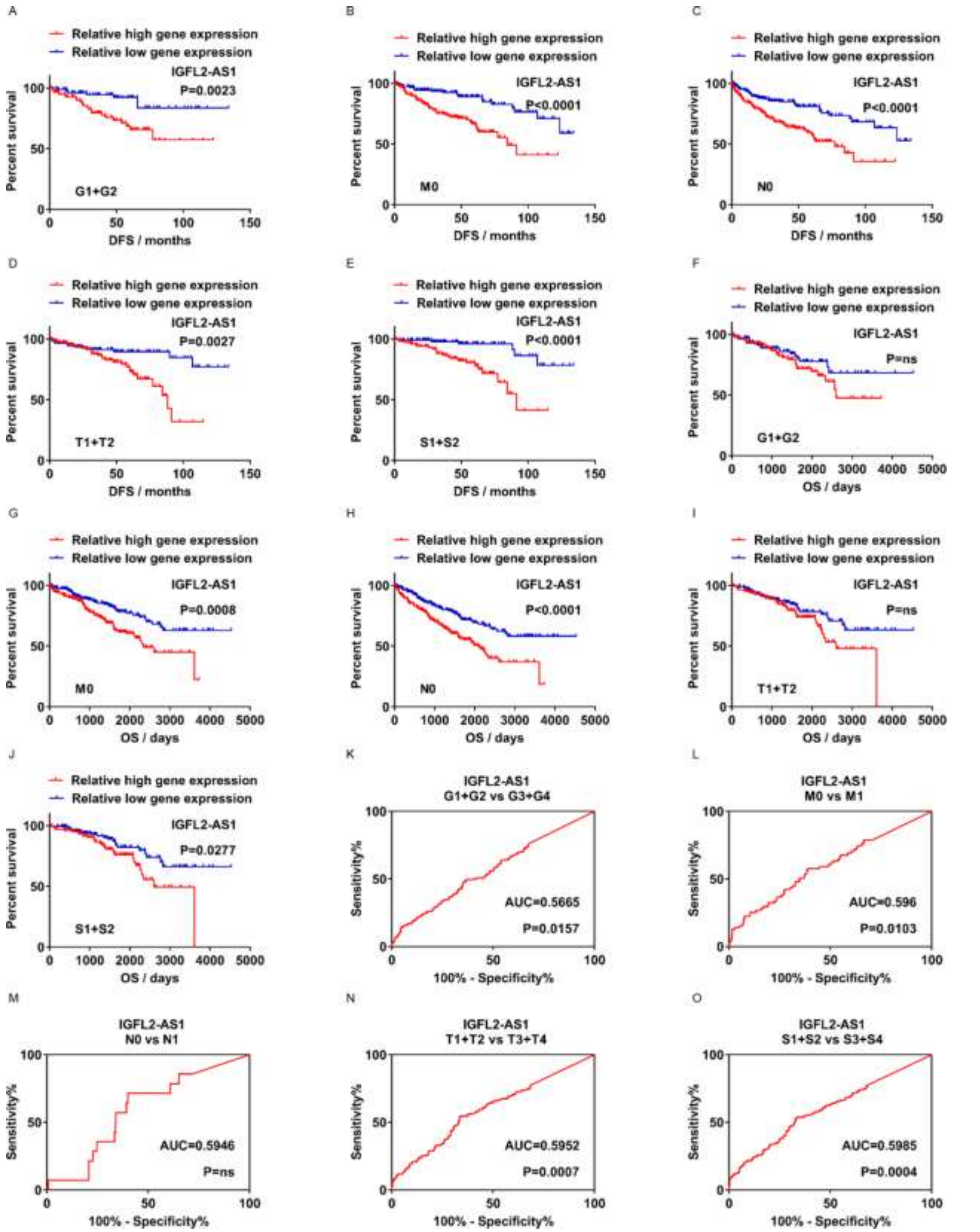

Supplementary Figure 7. Detailed information about IGFL2-AS1 regarding DFS, OS and ROC analysis. (**A**–**J**) DFS and OS analysis were conducted respectively regarding G1+G2, M0, N0, T1+T2, S1+S2 subgroups. (**K**–**O**) ROC curves were performed to distinguish G1+G2 vs G3+G4, M0 vs M1, N0 vs N1, T1+T2 vs T3+T4, S1+S2 vs S3+S4 patient samples.

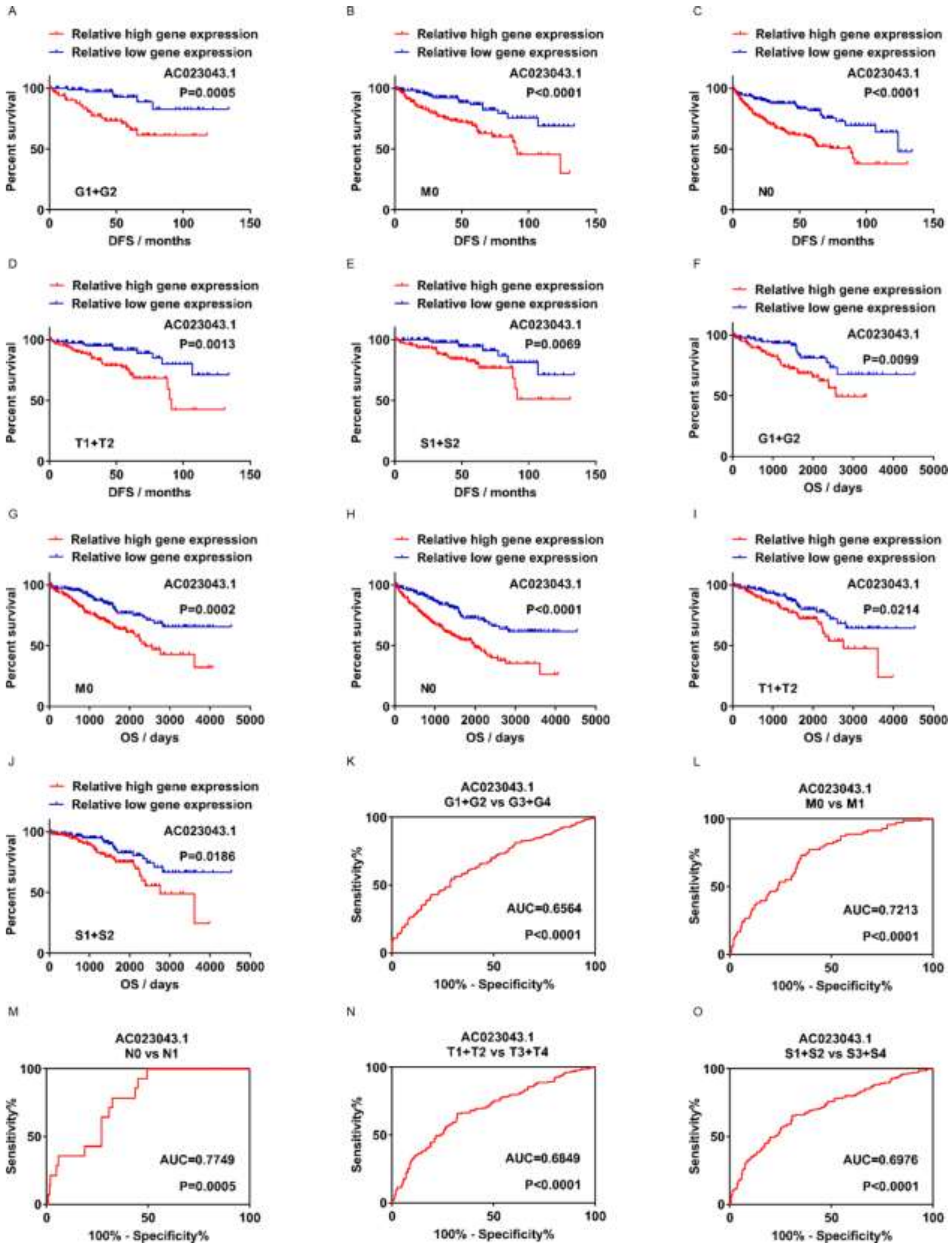

Supplementary Figure 8. Detailed information about AC023043.1 regarding DFS, OS and ROC analysis. (**A**–**J**) DFS and OS analysis were conducted respectively regarding G1+G2, M0, N0, T1+T2, S1+S2 subgroups. (**K**–**O**) ROC curves were performed to distinguish G1+G2 vs G3+G4, M0 vs M1, N0 vs N1, T1+T2 vs T3+T4, S1+S2 vs S3+S4 patient samples.

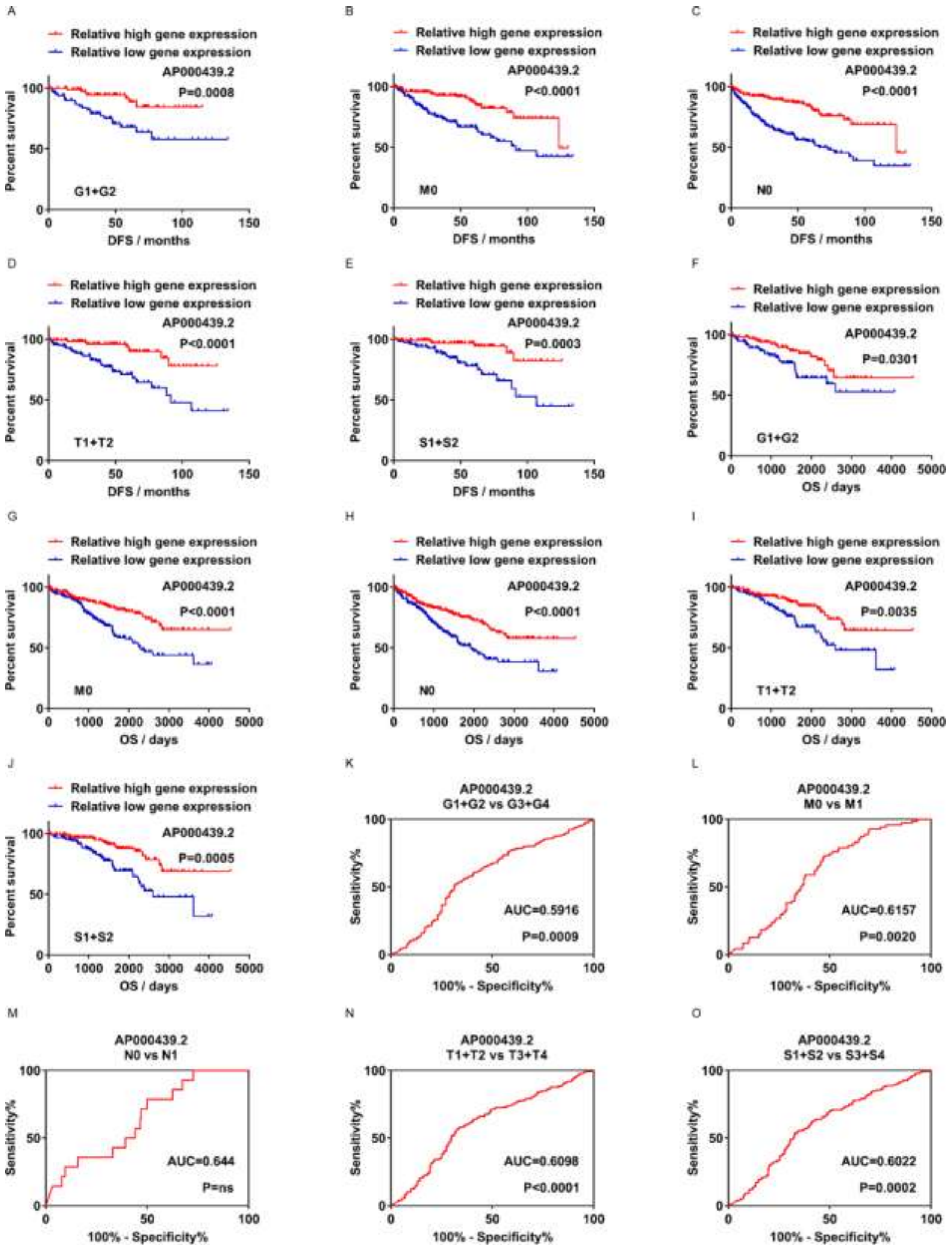

Supplementary Figure 9. Detailed information about AP000439.2 regarding DFS, OS and ROC analysis. (**A**–**J**) DFS and OS analysis were conducted respectively regarding G1+G2, M0, N0, T1+T2, S1+S2 subgroups. (**K**–**O**) ROC curves were performed to distinguish G1+G2 vs G3+G4, M0 vs M1, N0 vs N1, T1+T2 vs T3+T4, S1+S2 vs S3+S4 patient samples.

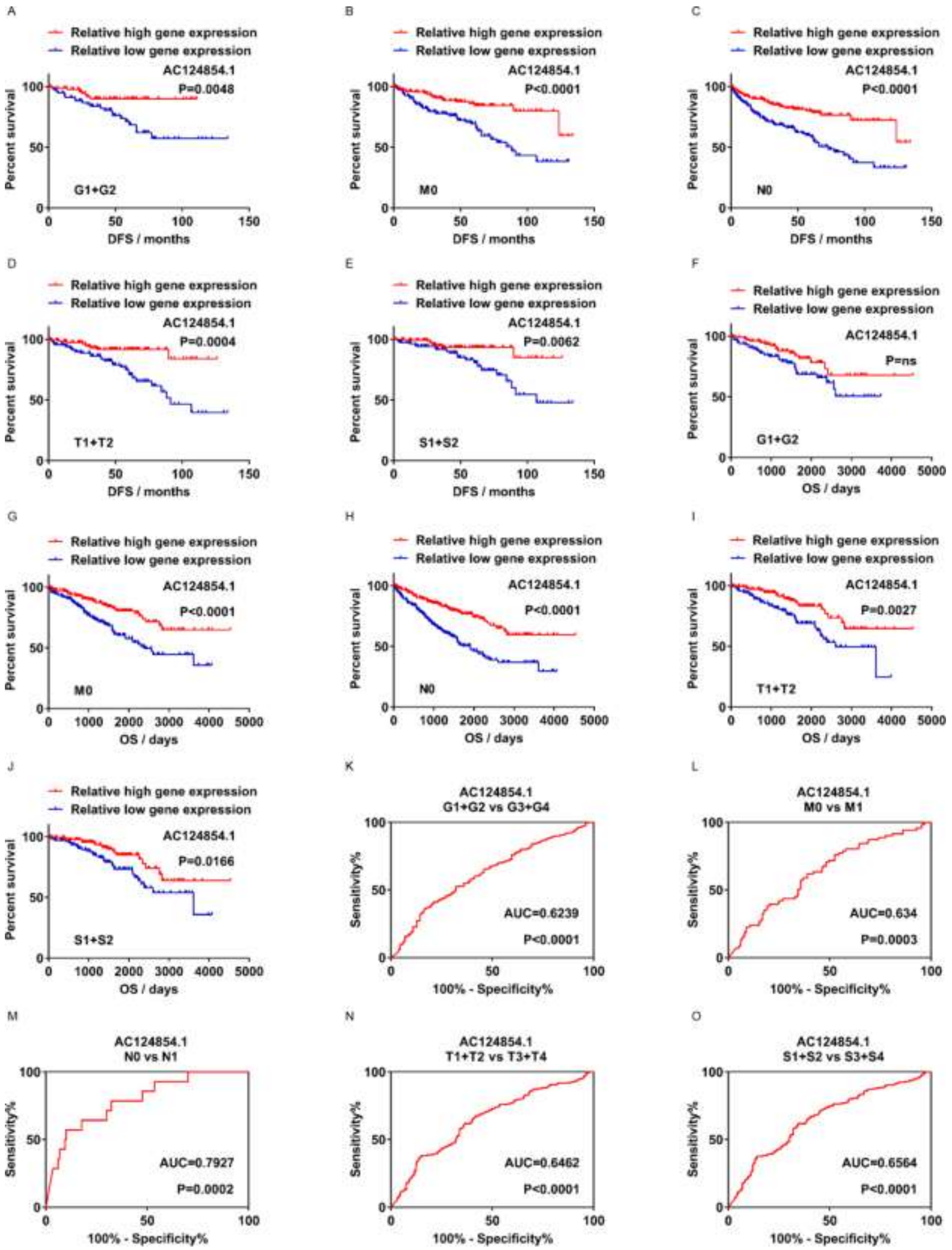

Supplementary Figure 10. Detailed information about AC124854.1 regarding DFS, OS and ROC analysis. (**A**–**J**) DFS and OS analysis were conducted respectively regarding G1+G2, M0, N0, T1+T2, S1+S2 subgroups. (**K**–**O**) ROC curves were performed to distinguish G1+G2 vs G3+G4, M0 vs M1, N0 vs N1, T1+T2 vs T3+T4, S1+S2 vs S3+S4 patient samples.

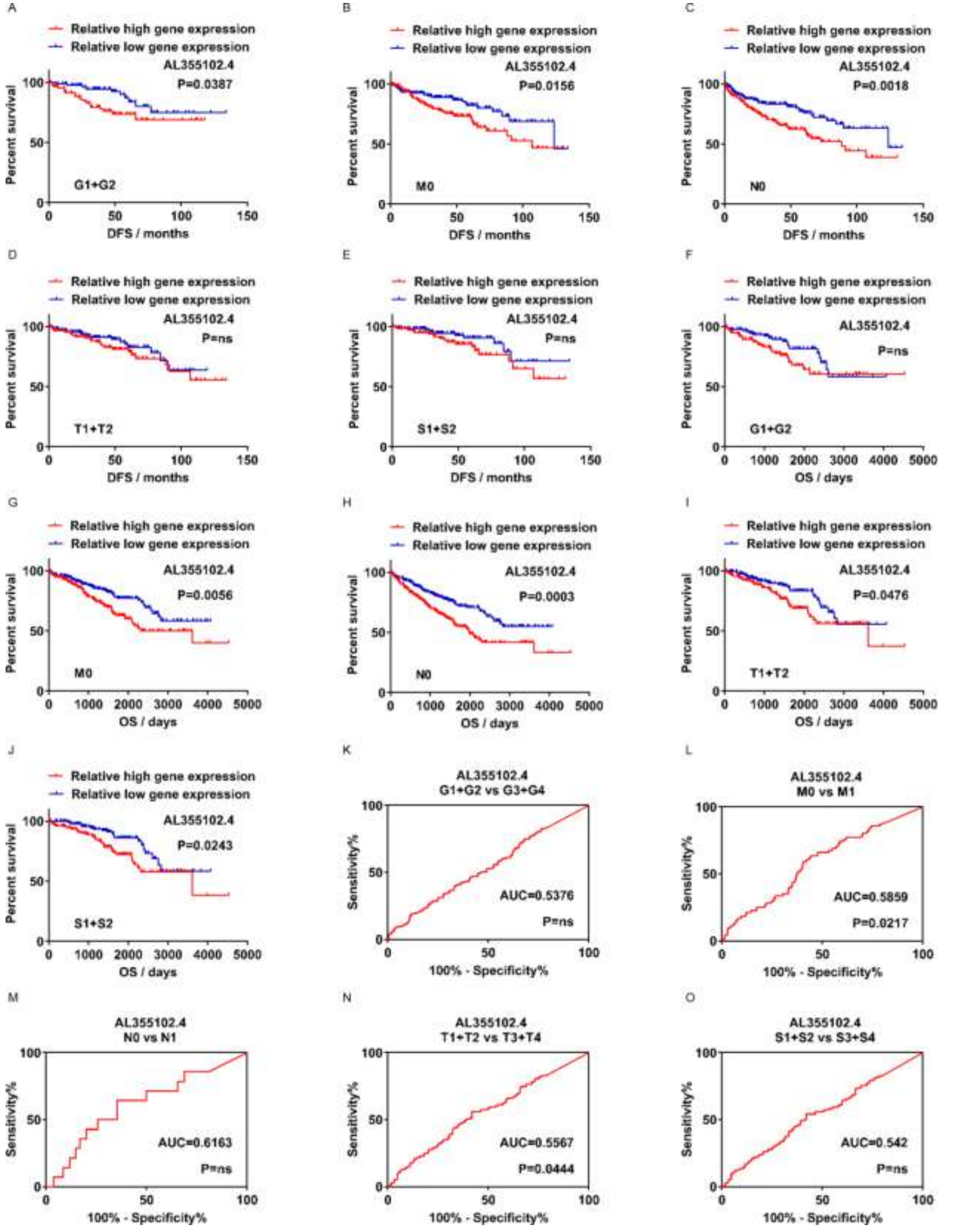

Supplementary Figure 11. Detailed information about AL355102.4 regarding DFS, OS and ROC analysis. (**A**–**J**) DFS and OS analysis were conducted respectively regarding G1+G2, M0, N0, T1+T2, S1+S2 subgroups. (**K**–**O**) ROC curves were performed to distinguish G1+G2 vs G3+G4, M0 vs M1, N0 vs N1, T1+T2 vs T3+T4, S1+S2 vs S3+S4 patient samples.

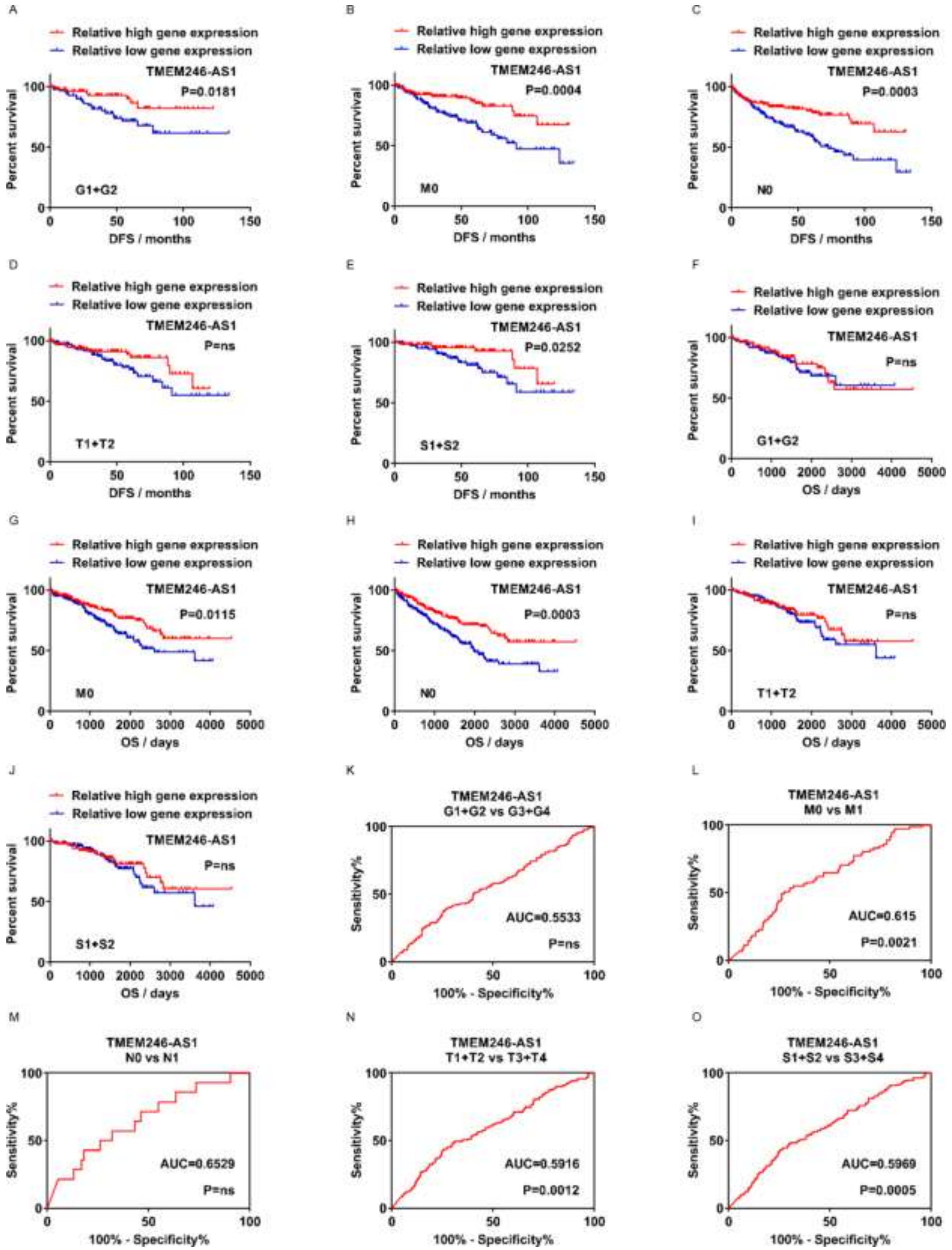

Supplementary Figure 12. Detailed information about TMEM246-AS1 regarding DFS, OS and ROC analysis. (**A**–**J**) DFS and OS analysis were conducted respectively regarding G1+G2, M0, N0, T1+T2, S1+S2 subgroups. (**K**–**O**) ROC curves were performed to distinguish G1+G2 vs G3+G4, M0 vs M1, N0 vs N1, T1+T2 vs T3+T4, S1+S2 vs S3+S4 patient samples.

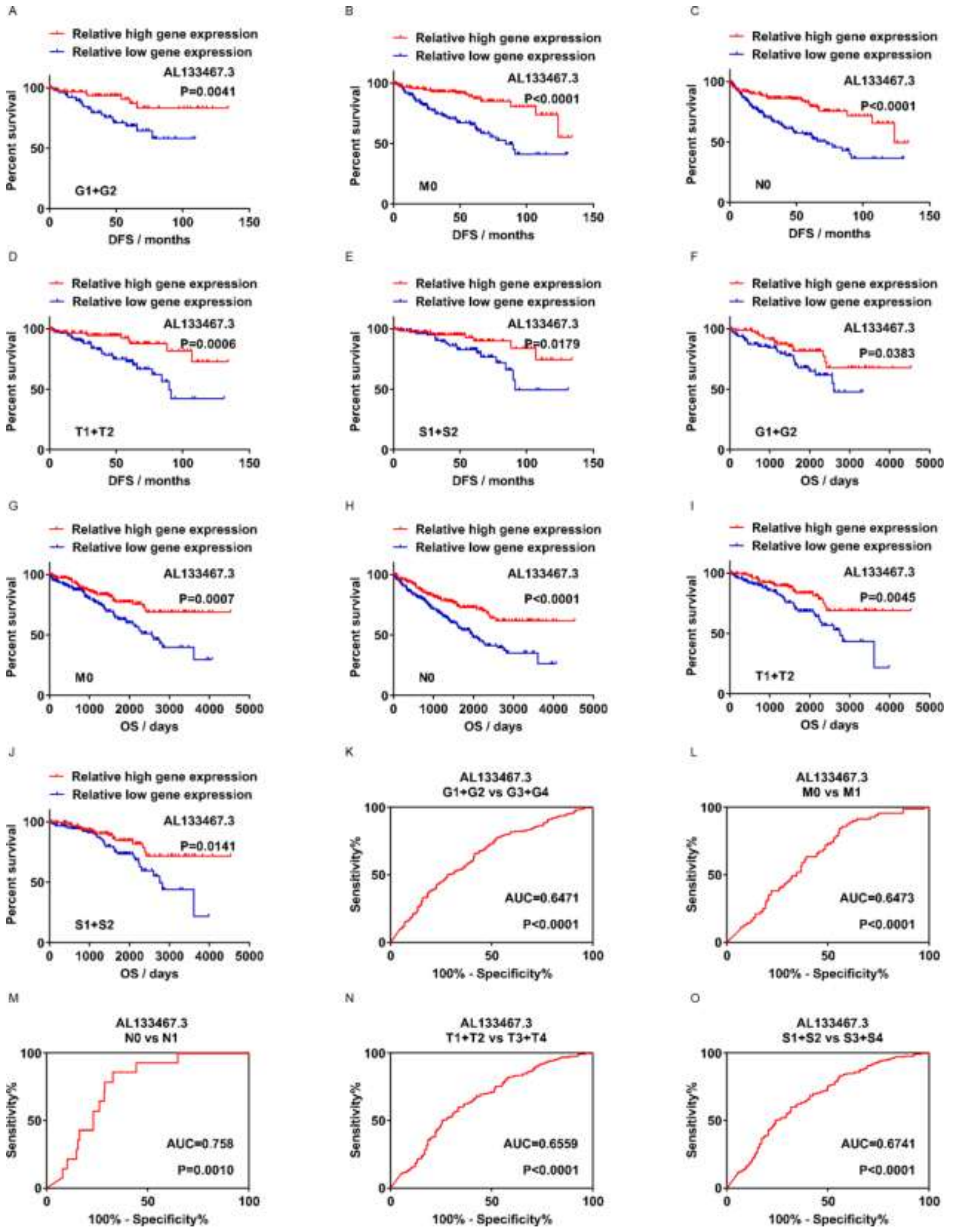

Supplementary Figure 13. Detailed information about AL133467.3 regarding DFS, OS and ROC analysis. (**A**–**J**) DFS and OS analysis were conducted respectively regarding G1+G2, M0, N0, T1+T2, S1+S2 subgroups. (**K**–**O**) ROC curves were performed to distinguish G1+G2 vs G3+G4, M0 vs M1, N0 vs N1, T1+T2 vs T3+T4, S1+S2 vs S3+S4 patient samples.

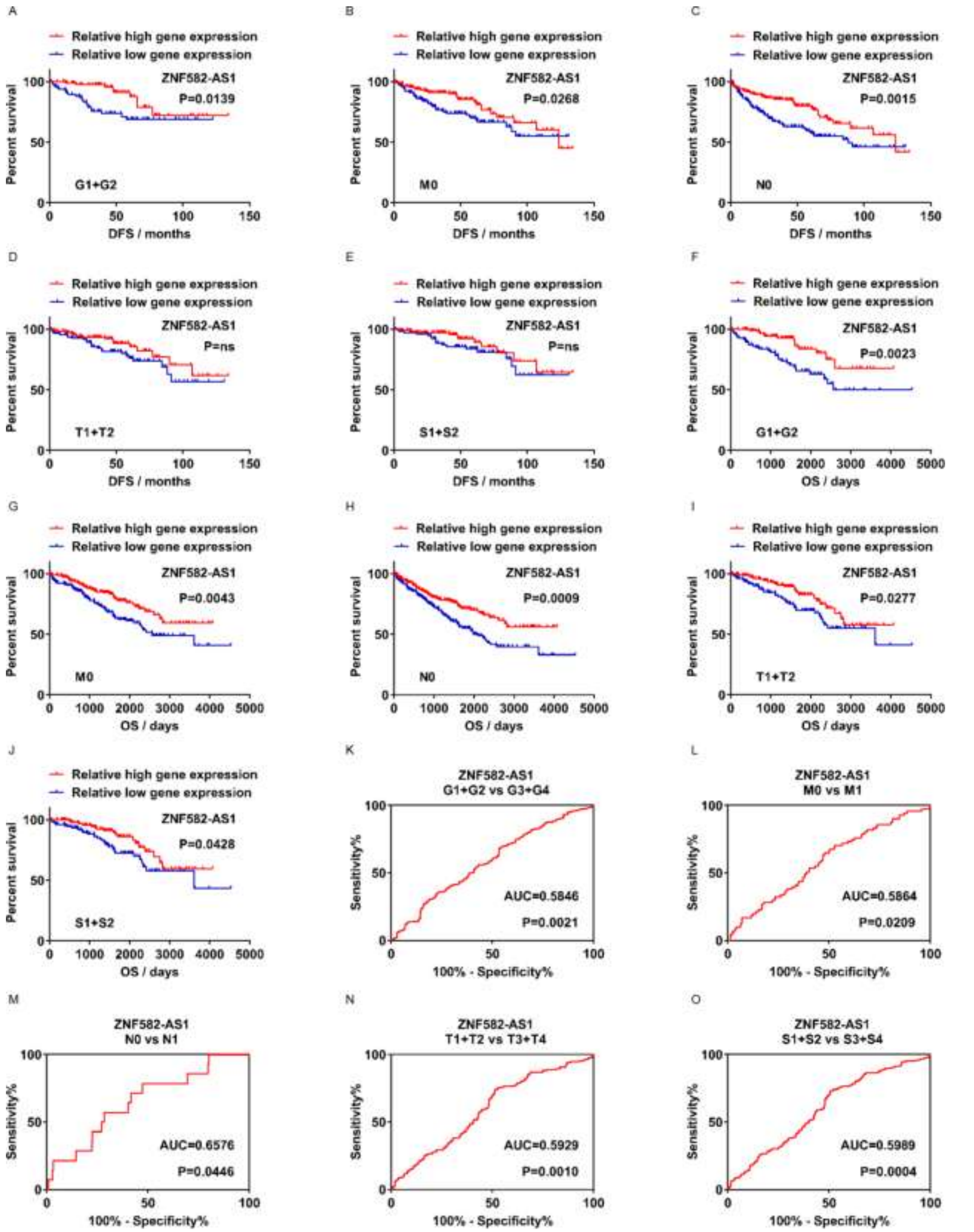

Supplementary Figure 14. Detailed information about ZNF582-AS1 regarding DFS, OS and ROC analysis. (**A**–**J**) DFS and OS analysis were conducted respectively regarding G1+G2, M0, N0, T1+T2, S1+S2 subgroups. (**K**–**O**) ROC curves were performed to distinguish G1+G2 vs G3+G4, M0 vs M1, N0 vs N1, T1+T2 vs T3+T4, S1+S2 vs S3+S4 patient samples.

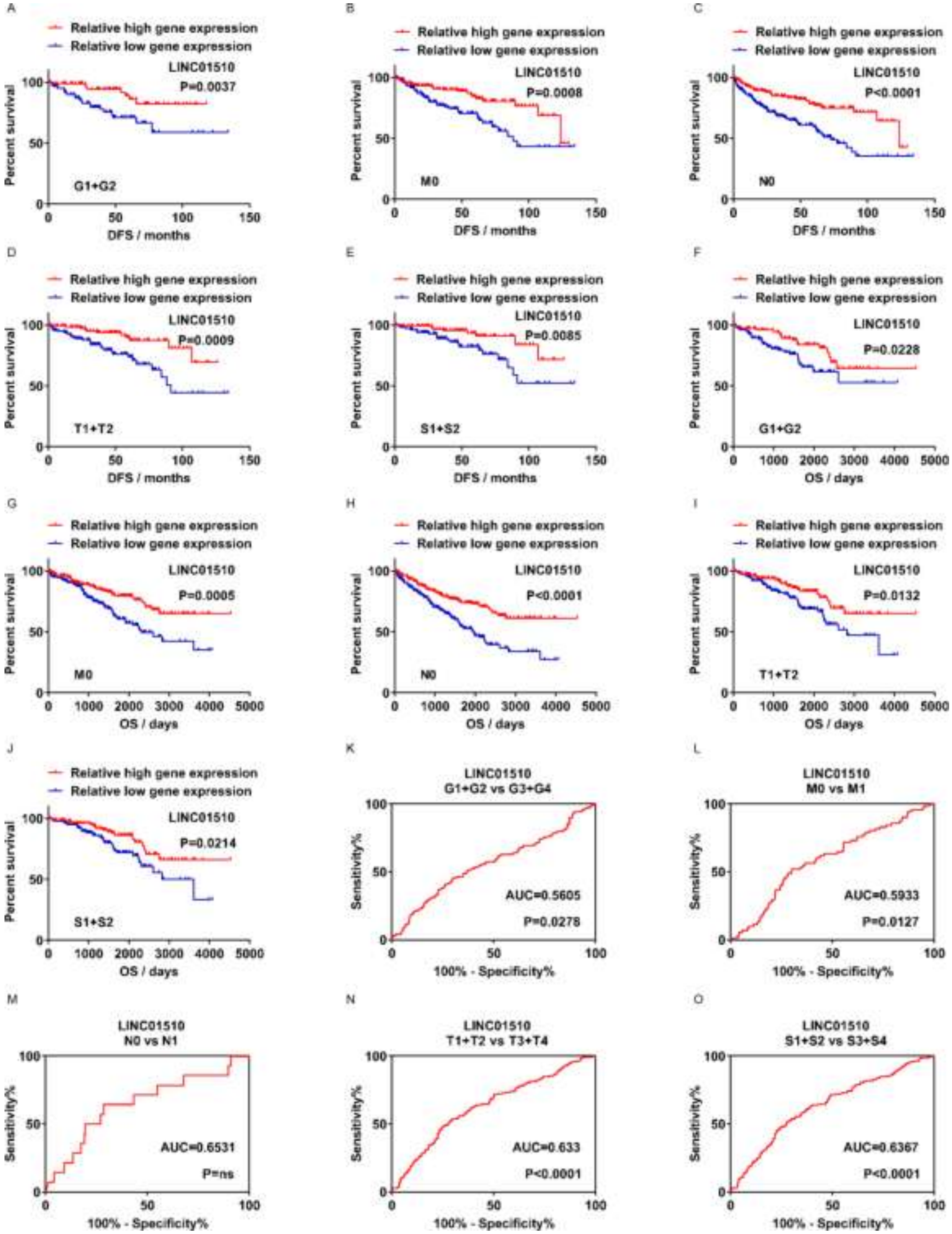

Supplementary Figure 15. Detailed information about LINC01510 regarding DFS, OS and ROC analysis. (**A**–**J**) DFS and OS analysis were conducted respectively regarding G1+G2, M0, N0, T1+T2, S1+S2 subgroups. (**K**–**O**) ROC curves were performed to distinguish G1+G2 vs G3+G4, M0 vs M1, N0 vs N1, T1+T2 vs T3+T4, S1+S2 vs S3+S4 patient samples.

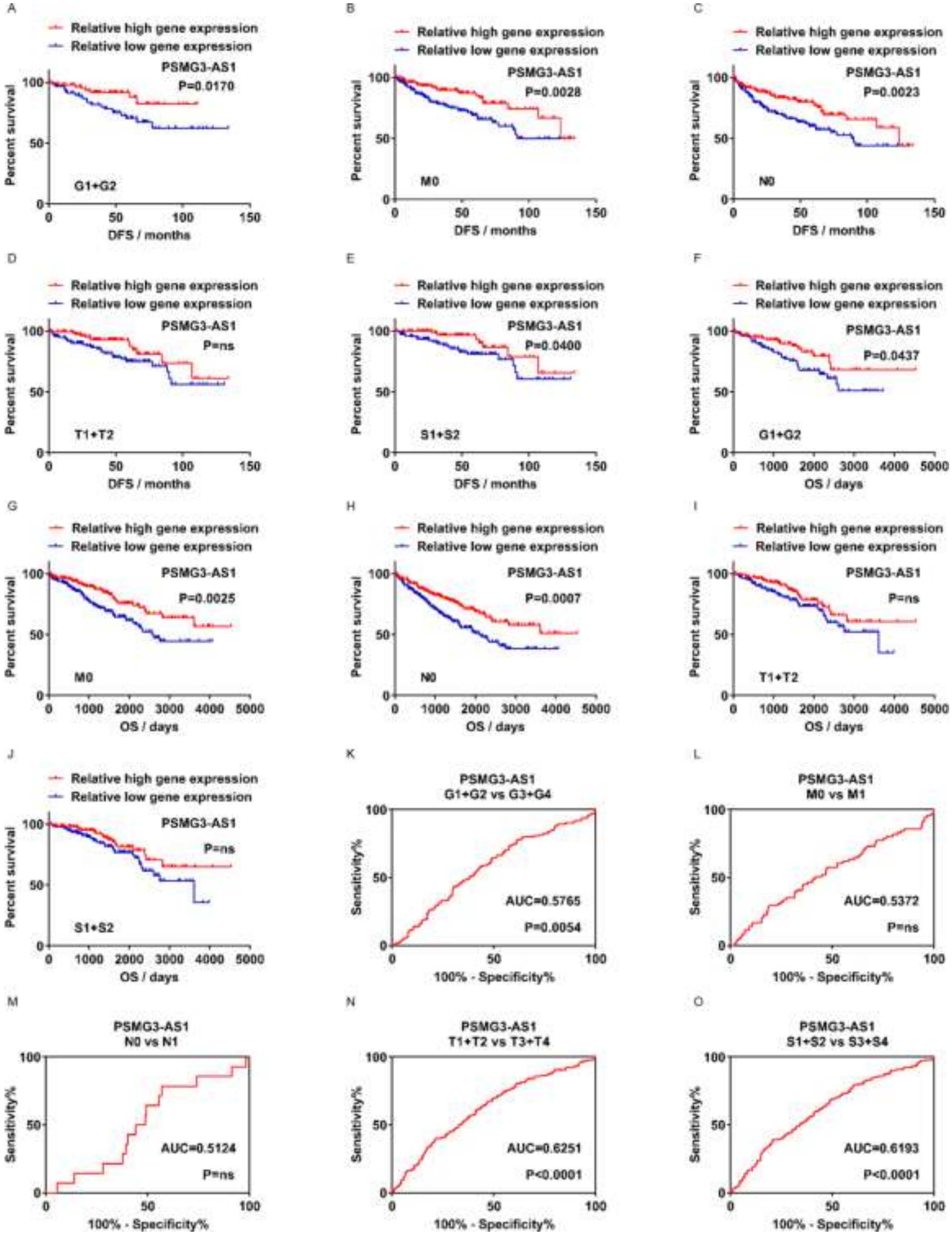

Supplementary Figure 16. Detailed information about PSMG3-AS1 regarding DFS, OS and ROC analysis. (**A**–**J**) DFS and OS analysis were conducted respectively regarding G1+G2, M0, N0, T1+T2, S1+S2 subgroups. (**K**–**O**) ROC curves were performed to distinguish G1+G2 vs G3+G4, M0 vs M1, N0 vs N1, T1+T2 vs T3+T4, S1+S2 vs S3+S4 patient samples.

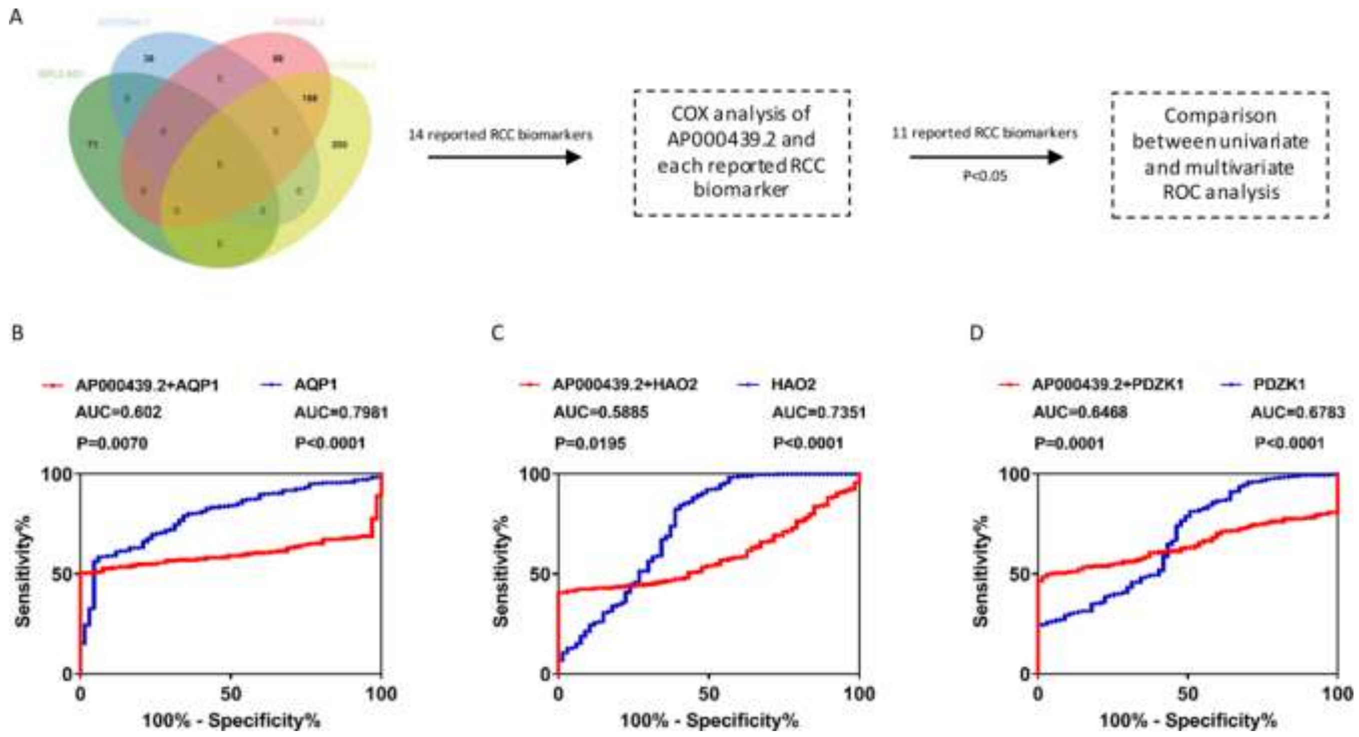

Supplementary Figure 17. The diagnostic accuracy comparison between univariate and multivariate RCC biomarkers under ROC curves. (**A**) Flow chart of the diagnostic accuracy comparison. (**B**–**D**) No improvement of diagnostic accuracy when combining AP000439.2 with AQP1 (**B**), HAO2 (**C**) and PDZK1 (**D**).

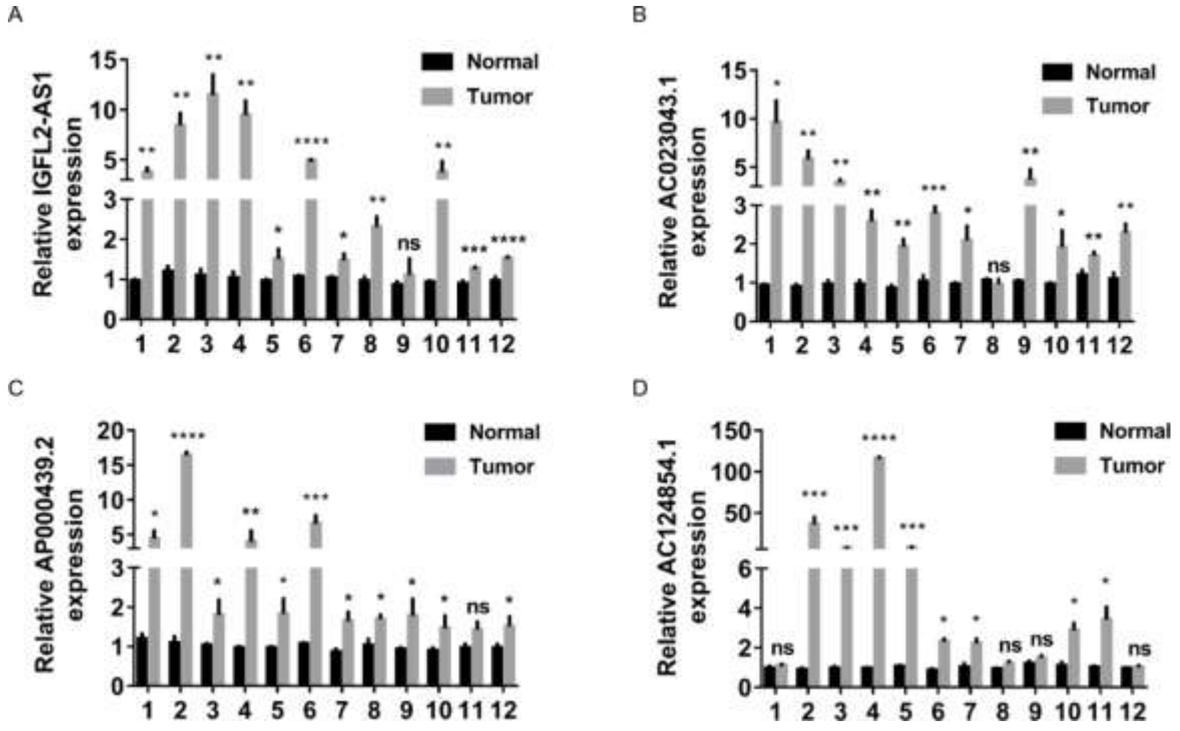

Supplementary Figure 18. RT-PCR results of 4 upregulated lncRNAs in twelve paired kidney cancer tissues. (**A**–**D**) RT-PCR results of IGFL2-AS1 (**A**), AC023043.1 (**B**), AP000439.2 (**C**) and AC124854.1 (**D**) in twelve paired kidney cancer tissues.

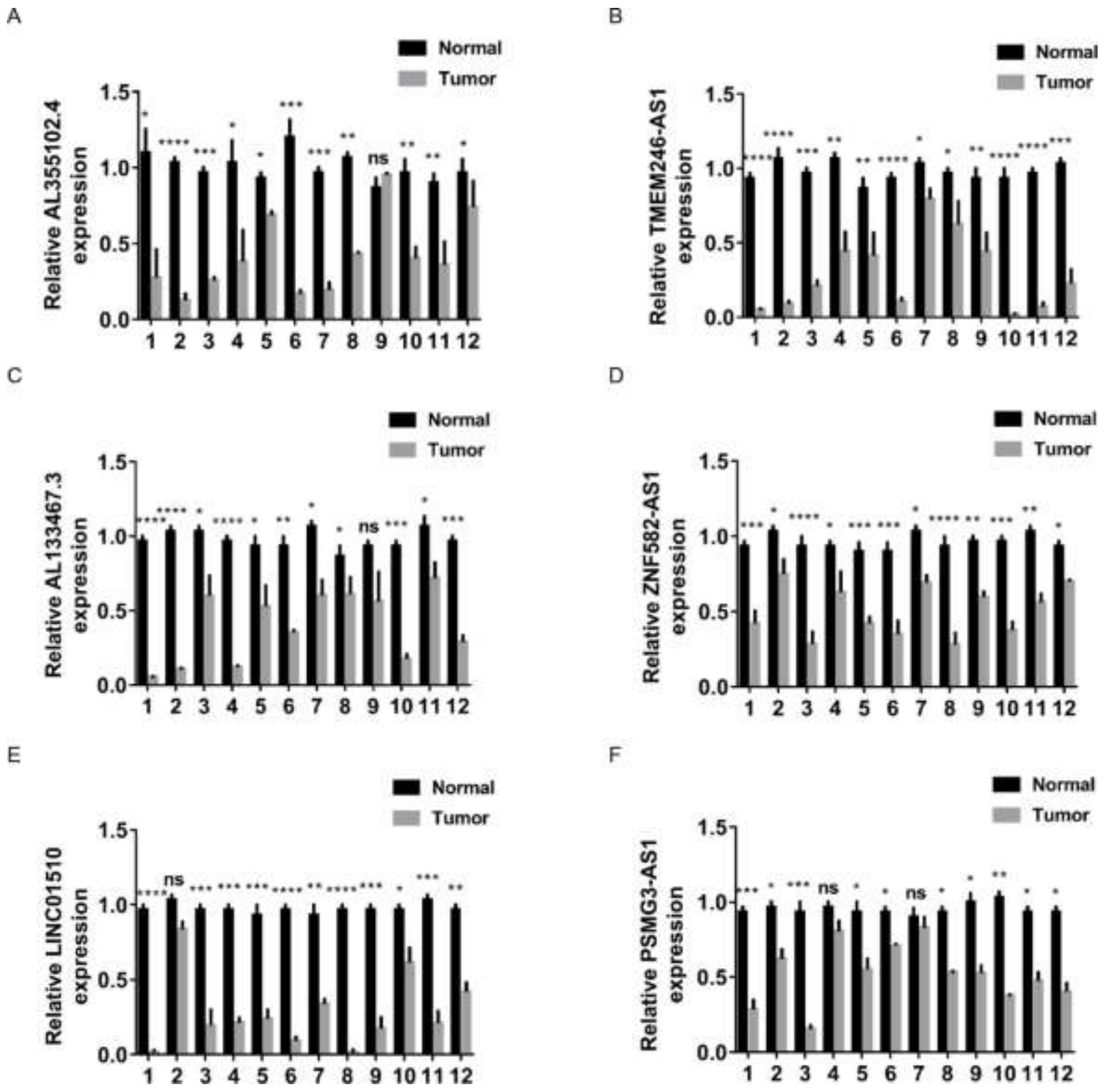

Supplementary Figure 19. RT-PCR results of 6 downregulated lncRNAs in twelve paired kidney cancer tissues. (**A**–**F**) RT-PCR results of AL355102.4 (**A**), TMEM246-AS1 (**B**), AL133467.3 (**C**), ZNF582-AS1 (**D**), LINC01510 (**E**) and PSMG3-AS1 (**F**) in twelve paired kidney cancer tissues.

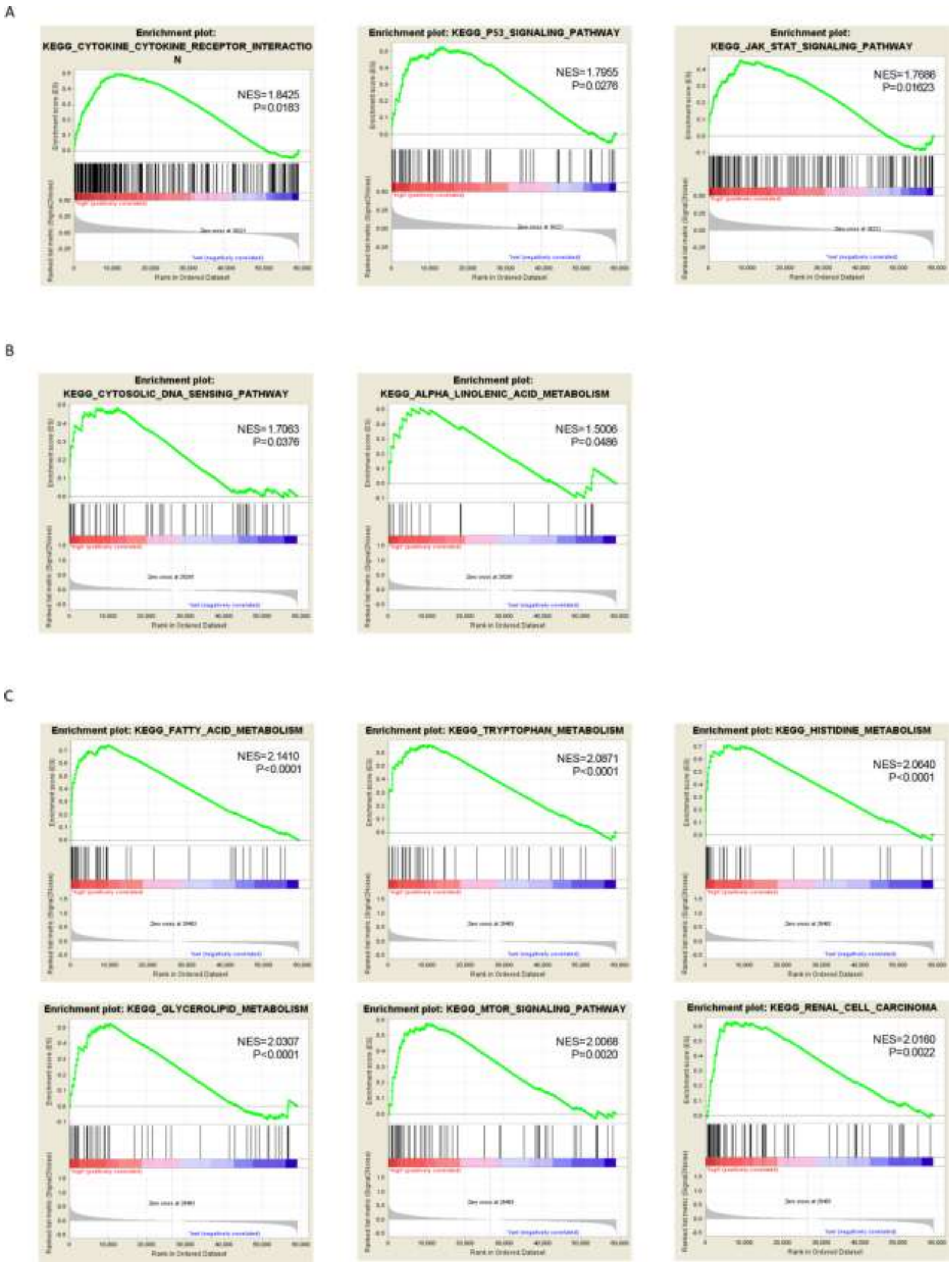

Supplementary Figure 20. GSEA pathways about IGFL2-AS1, AC023043.1 and AP000439.2. (**A**–**C**) Enrichment pathways of IGFL2-AS1 (**A**), AC023043.1 (**B**) and AP000439.2 (**C**) were shown after GSEA analysis respectively.

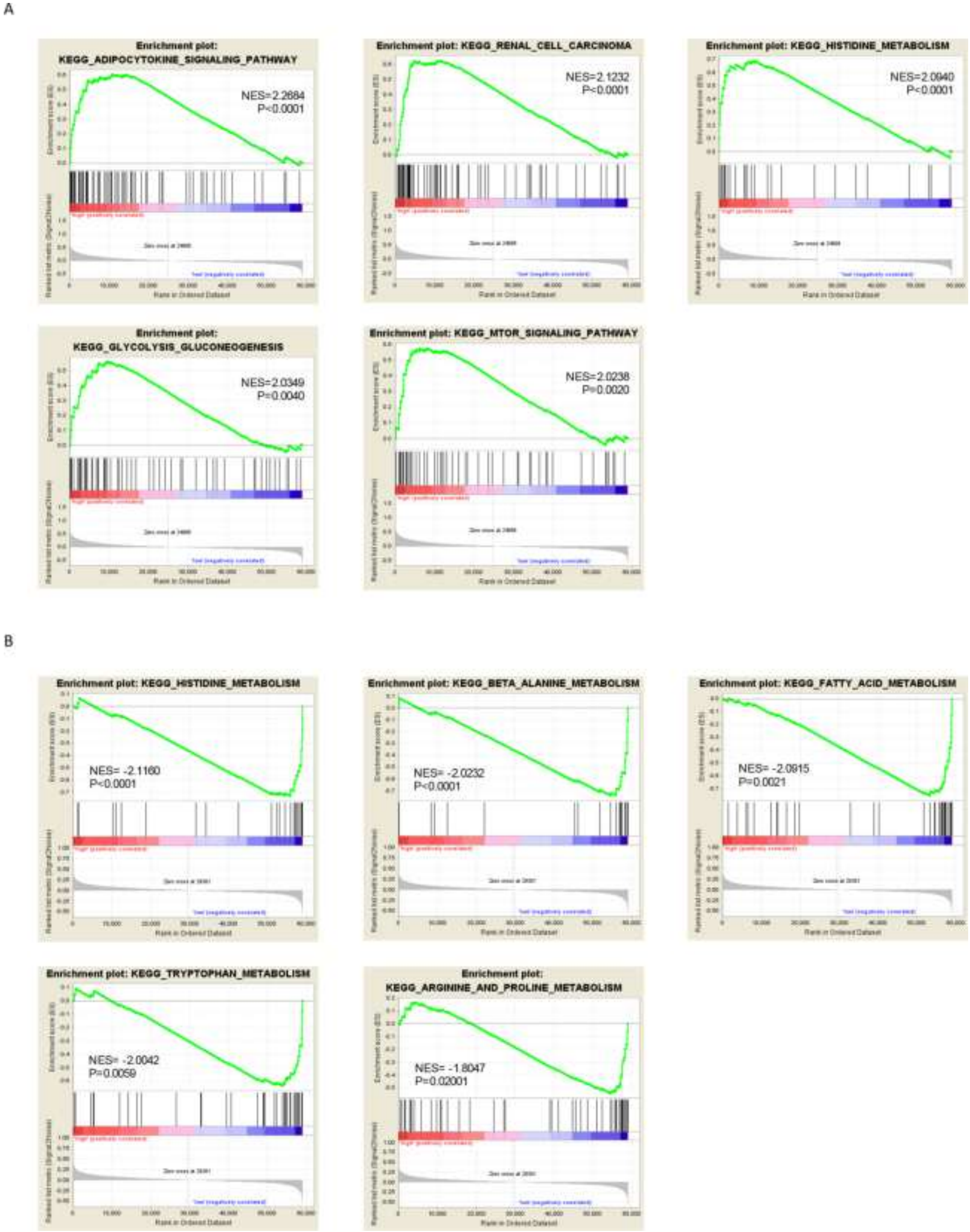

Supplementary Figure 21. GSEA pathways about AC124854.1 and AL355102.4. (**A**–**B**) Enrichment pathways of AC124854.1 (**A**) and AL355102.4 (**B**) were shown after GSEA analysis respectively.

A

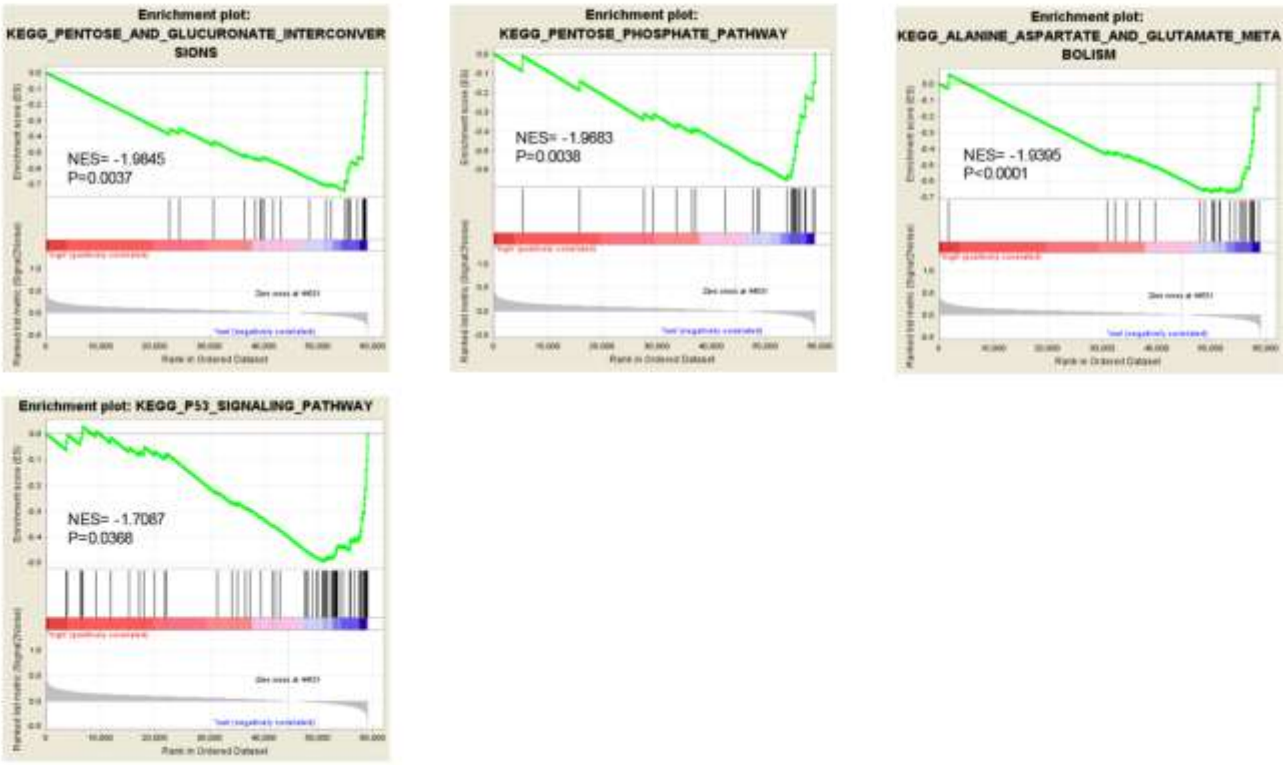

B

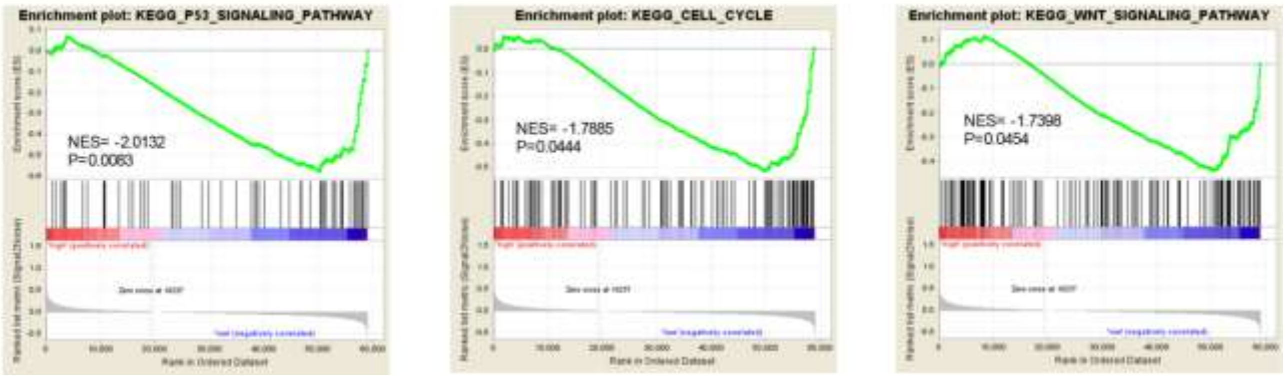

Supplementary Figure 22. GSEA pathways about ZNF582-AS1 and LINC01510. (**A**–**B**) Enrichment pathways of ZNF582-AS1 (**A**) and LINC01510 (**B**) were shown after GSEA analysis respectively.

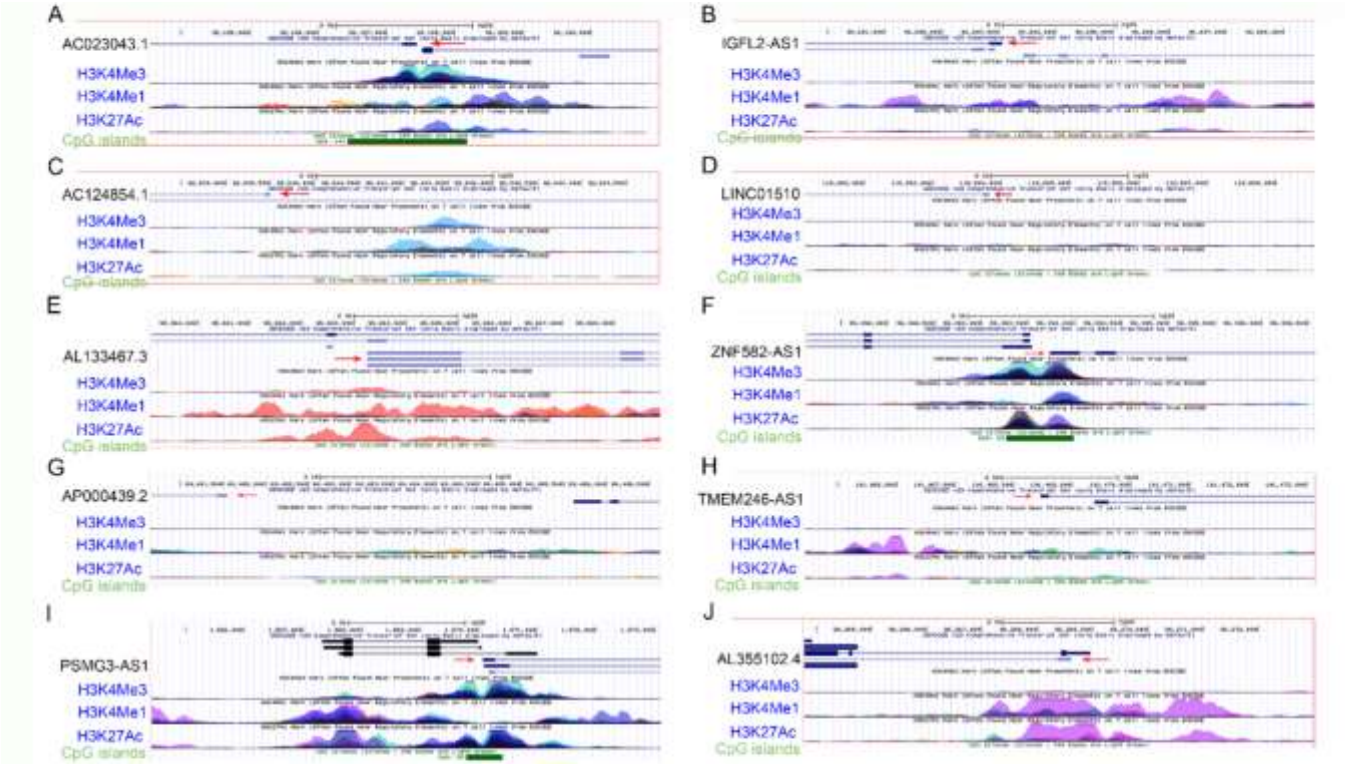

Supplementary Figure 23. Histone acetylation and methylation status of 10 candidate lncRNAs at promoter regions. (**A**–**J**) Histone acetylation and methylation status of 10 candidate lncRNAs at promoter regions were shown based on UCSC Genome Browser (GRCh38/hg38).

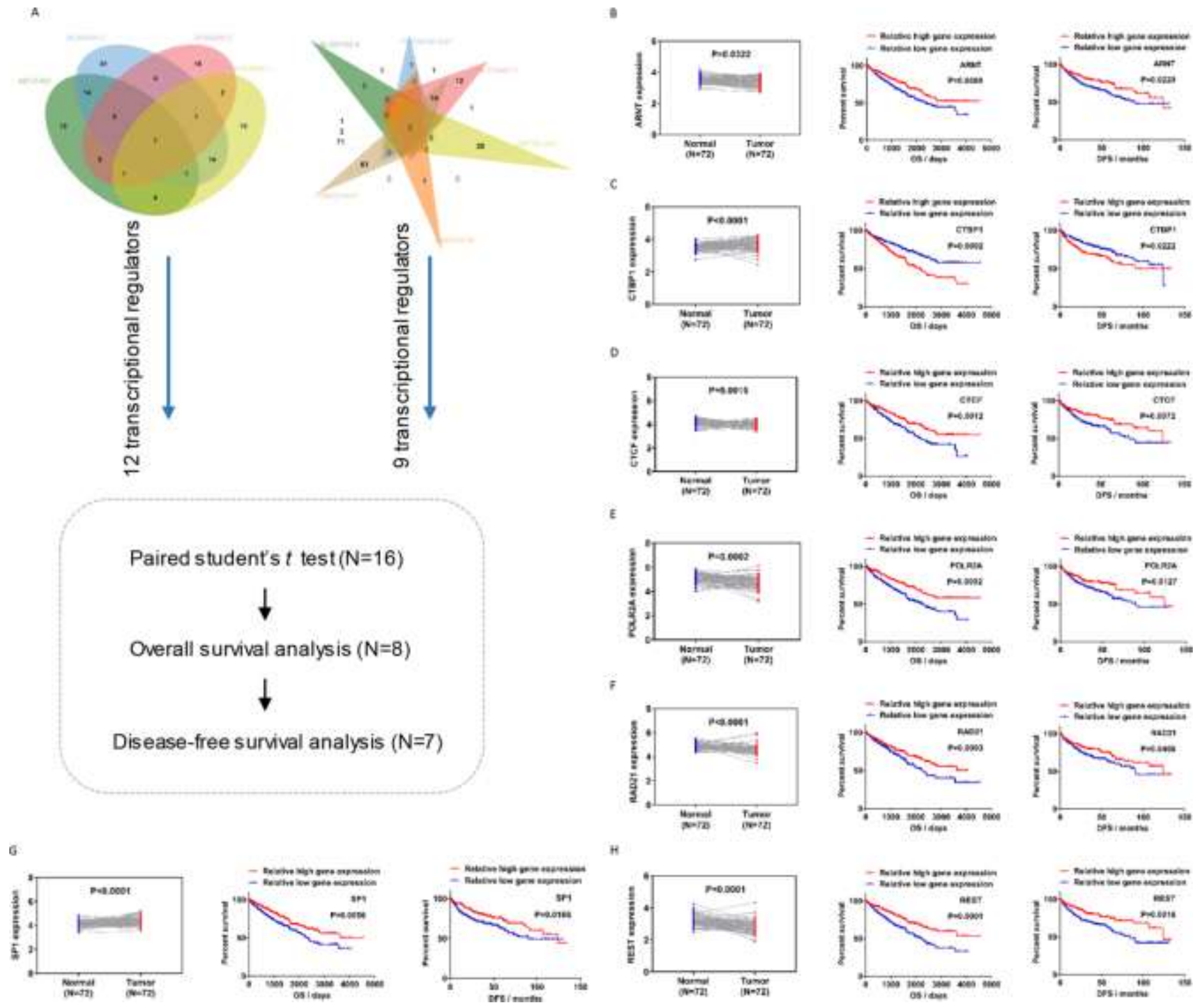

Supplementary Figure 24. Clinical values of transcriptional regulators of candidate lncRNAs. (**A**) Flow chart of transcriptional regulators screening. (**B**–**H**) 7 transcriptional regulators of 72 paired normal and tumor samples exhibited statistical difference after student's *t*-test. Each transcriptional regulator exhibited statistically significant difference between relative high gene expression and low gene expression regarding DFS and OS.
