# Supplementary material for: A cluster of long non-coding RNAs exhibit diagnostic and prognostic values in renal cell carcinoma
Source: Aging (Albany NY). 2019 Nov 14;11(21):9597–615. doi: 10.18632/aging.102407 (PMC6874440; doi:10.18632/aging.102407)
Supplement: Supplementary Table 1 [file aging-11-102407-s002.docx]

Supplementary Table 1. 125 upregulated lncRNAs after differential expression analysis.

| **Gene_symbol** | **Annotation** | **log_2_FC** | **log_2_CPM** | **adjusted P Value** | **FDR** |
| --- | --- | --- | --- | --- | --- |
| ENSG00000237517.4 | DGCR5 | 4.001864 | 10.75858 | 1.68E-53 | 2.43E-51 |
| ENSG00000224490.1 | TTC21B-AS1 | 6.356893 | 11.34752 | 7.25E-48 | 8.72E-46 |
| ENSG00000247095.2 | MIR210HG | 2.731326 | 10.79282 | 7.52E-47 | 7.76E-45 |
| ENSG00000240758.2 | AC010655.2 | 4.209171 | 10.92526 | 4.46E-44 | 3.58E-42 |
| ENSG00000227869.1 | AC073486.1 | 3.757989 | 12.96963 | 2.48E-42 | 1.49E-40 |
| ENSG00000261827.1 | ENSG00000261827 | 2.883259 | 10.52664 | 1.51E-39 | 8.40E-38 |
| ENSG00000214145.2 | LINC00887 | 4.195643 | 10.68383 | 2.52E-38 | 1.30E-36 |
| ENSG00000177410.8 | ZFAS1 | 1.83657 | 14.83188 | 1.44E-37 | 6.13E-36 |
| ENSG00000255774.1 | AP000439.2 | 4.971501 | 10.86061 | 7.15E-34 | 2.46E-32 |
| ENSG00000233610.1 | LINC00462 | 5.110454 | 11.60389 | 7.97E-34 | 2.62E-32 |
| ENSG00000267449.1 | AC005884.2 | 1.173048 | 14.20849 | 4.77E-32 | 1.38E-30 |
| ENSG00000237686.2 | AL109615.3 | 2.653694 | 10.36221 | 5.52E-32 | 1.53E-30 |
| ENSG00000273032.1 | DGCR9 | 3.159145 | 10.13363 | 1.46E-31 | 3.77E-30 |
| ENSG00000256234.1 | AC022509.2 | 4.405419 | 10.55649 | 4.26E-31 | 1.06E-29 |
| ENSG00000224032.2 | EPB41L4A-AS1 | 1.585811 | 12.48949 | 1.04E-28 | 2.09E-27 |
| ENSG00000232271.1 | AL096799.1 | 3.64481 | 10.21739 | 6.49E-28 | 1.23E-26 |
| ENSG00000257702.3 | LBX2-AS1 | 1.570308 | 11.54965 | 1.86E-26 | 3.27E-25 |
| ENSG00000234741.3 | GAS5 | 1.459795 | 12.59754 | 3.20E-26 | 5.05E-25 |
| ENSG00000255750.1 | AC022509.1 | 3.912393 | 10.1408 | 3.22E-26 | 5.05E-25 |
| ENSG00000267207.1 | AC005884.1 | 1.015573 | 12.91253 | 7.24E-26 | 1.09E-24 |
| ENSG00000269985.1 | AL021328.1 | 3.253752 | 10.0009 | 1.15E-25 | 1.70E-24 |
| ENSG00000261175.1 | LINC02188 | 3.221432 | 10.13557 | 1.24E-25 | 1.79E-24 |
| ENSG00000229953.1 | AL590666.2 | 2.794714 | 10.08707 | 2.39E-25 | 3.38E-24 |
| ENSG00000206337.6 | HCP5 | 1.407188 | 12.25465 | 2.49E-25 | 3.46E-24 |
| ENSG00000233627.2 | C4A-AS1 | 2.435237 | 11.22185 | 1.26E-24 | 1.69E-23 |
| ENSG00000241732.1 | ENSG00000241732 | 2.527435 | 10.24833 | 1.30E-24 | 1.71E-23 |
| ENSG00000255462.1 | AC104031.1 | 2.459804 | 10.73627 | 8.74E-24 | 1.07E-22 |
| ENSG00000256694.1 | AC026369.2 | 2.912125 | 9.976271 | 2.72E-23 | 3.27E-22 |
| ENSG00000233456.1 | LINC01077 | 4.250474 | 10.56211 | 8.18E-22 | 9.22E-21 |
| ENSG00000245910.4 | SNHG6 | 1.10155 | 12.98921 | 1.09E-21 | 1.19E-20 |
| ENSG00000248223.1 | AC026785.2 | 1.101877 | 11.80159 | 1.67E-21 | 1.80E-20 |
| ENSG00000230177.1 | AL080317.1 | 2.334528 | 10.4889 | 2.41E-21 | 2.56E-20 |
| ENSG00000230432.1 | AC114803.1 | 3.613338 | 10.4125 | 2.53E-21 | 2.61E-20 |
| ENSG00000272870.1 | AC097534.2 | 2.384609 | 10.09981 | 3.27E-21 | 3.33E-20 |
| ENSG00000222041.6 | CYTOR | 1.650776 | 11.10978 | 5.05E-21 | 5.07E-20 |
| ENSG00000247134.2 | AC090204.1 | 2.172314 | 10.8092 | 9.48E-21 | 9.37E-20 |
| ENSG00000232153.2 | AC073218.1 | 2.268389 | 10.42068 | 1.70E-20 | 1.66E-19 |
| ENSG00000258667.1 | HIF1A-AS2 | 2.977564 | 10.0043 | 2.76E-20 | 2.62E-19 |
| ENSG00000224057.1 | EGFR-AS1 | 4.838806 | 11.43819 | 7.58E-20 | 7.11E-19 |
| ENSG00000271762.1 | ENSG00000271762 | 3.843772 | 10.10936 | 2.02E-19 | 1.87E-18 |
| ENSG00000197989.9 | SNHG12 | 2.186617 | 10.13301 | 7.06E-19 | 6.21E-18 |
| ENSG00000247774.2 | PCED1B-AS1 | 2.478985 | 10.09843 | 8.96E-19 | 7.79E-18 |
| ENSG00000273001.1 | AL731533.2 | 2.010382 | 10.21395 | 1.02E-18 | 8.77E-18 |
| ENSG00000251442.1 | LINC01094 | 2.619557 | 9.977209 | 1.47E-18 | 1.25E-17 |
| ENSG00000245694.4 | CRNDE | 1.573723 | 10.77671 | 1.56E-18 | 1.31E-17 |
| ENSG00000269640.1 | ENSG00000269640 | 1.713777 | 10.37462 | 7.51E-18 | 6.09E-17 |
| ENSG00000224818.1 | AC096677.2 | 1.850479 | 10.46054 | 8.49E-18 | 6.81E-17 |
| ENSG00000235142.3 | LINC02532 | 1.059118 | 11.90804 | 1.06E-17 | 8.36E-17 |
| ENSG00000225969.1 | ABHD11-AS1 | 2.288285 | 10.0848 | 2.87E-17 | 2.23E-16 |
| ENSG00000251169.2 | LINC01843 | 2.043483 | 10.18596 | 3.76E-17 | 2.88E-16 |
| ENSG00000255717.2 | SNHG1 | 1.409792 | 10.82295 | 5.79E-17 | 4.40E-16 |
| ENSG00000203875.6 | SNHG5 | 1.266787 | 13.15438 | 6.20E-17 | 4.67E-16 |
| ENSG00000265743.1 | AC138207.5 | 2.110402 | 10.07073 | 7.44E-17 | 5.54E-16 |
| ENSG00000232956.4 | SNHG15 | 1.592863 | 10.58374 | 8.73E-17 | 6.43E-16 |
| ENSG00000257824.1 | AC068789.1 | 2.243374 | 10.17712 | 1.02E-16 | 7.47E-16 |
| ENSG00000253574.1 | AC021744.1 | 2.884243 | 11.07388 | 1.36E-16 | 9.80E-16 |
| ENSG00000261051.1 | AC107021.2 | 2.433743 | 9.985337 | 1.49E-16 | 1.07E-15 |
| ENSG00000259347.1 | AC087482.1 | 2.008868 | 11.22062 | 5.33E-16 | 3.63E-15 |
| ENSG00000251320.1 | AC011352.3 | 3.700293 | 10.07507 | 1.12E-15 | 7.56E-15 |
| ENSG00000204528.3 | PSORS1C3 | 2.353457 | 10.43137 | 1.15E-15 | 7.70E-15 |
| ENSG00000273445.1 | AC133644.2 | 2.892089 | 10.16898 | 1.26E-15 | 8.33E-15 |
| ENSG00000253859.2 | AC018616.1 | 1.01064 | 11.543 | 1.46E-15 | 9.60E-15 |
| ENSG00000255746.1 | AC007406.2 | 1.765694 | 10.65709 | 6.04E-15 | 3.79E-14 |
| ENSG00000172965.10 | MIR4435-2HG | 1.688768 | 10.2483 | 6.53E-15 | 4.07E-14 |
| ENSG00000237181.1 | AC147651.4 | 2.445184 | 10.09999 | 6.86E-15 | 4.24E-14 |
| ENSG00000236318.1 | AC019117.2 | 4.161333 | 12.11047 | 9.29E-15 | 5.68E-14 |
| ENSG00000231574.1 | LINC02015 | 2.483936 | 9.984582 | 1.47E-14 | 8.95E-14 |
| ENSG00000266976.1 | AC079466.1 | 4.576131 | 10.46797 | 7.39E-14 | 4.34E-13 |
| ENSG00000251301.2 | LINC02384 | 2.598525 | 10.49973 | 1.37E-13 | 7.91E-13 |
| ENSG00000269481.1 | AC010319.4 | 1.591459 | 10.23776 | 1.37E-13 | 7.91E-13 |
| ENSG00000251076.1 | AC104126.1 | 2.702663 | 10.64796 | 1.78E-13 | 1.02E-12 |
| ENSG00000234380.1 | LINC01426 | 2.430904 | 10.45512 | 3.82E-13 | 2.16E-12 |
| ENSG00000225138.3 | SLC9A3-AS1 | 1.843337 | 10.44028 | 4.56E-13 | 2.55E-12 |
| ENSG00000228262.4 | LINC01320 | 1.204218 | 12.25437 | 5.79E-13 | 3.21E-12 |
| ENSG00000230438.5 | SERPINB9P1 | 2.021385 | 10.08619 | 6.67E-13 | 3.68E-12 |
| ENSG00000249776.1 | AC124854.1 | 2.339875 | 10.26943 | 8.46E-13 | 4.63E-12 |
| ENSG00000257989.1 | AC078864.1 | 1.816261 | 10.11018 | 8.58E-13 | 4.66E-12 |
| ENSG00000249166.1 | AC092336.1 | 2.926475 | 11.48529 | 1.69E-12 | 9.03E-12 |
| ENSG00000232527.3 | AC245595.1 | 1.44816 | 11.78032 | 2.15E-12 | 1.11E-11 |
| ENSG00000249396.1 | LINC02212 | 2.896892 | 10.47397 | 2.65E-12 | 1.35E-11 |
| ENSG00000270547.1 | LINC01235 | 2.451752 | 10.22711 | 2.67E-12 | 1.35E-11 |
| ENSG00000231107.1 | LINC01508 | 1.457696 | 10.26649 | 2.88E-12 | 1.45E-11 |
| ENSG00000253374.1 | AC023644.1 | 1.000836 | 10.94194 | 3.78E-12 | 1.86E-11 |
| ENSG00000255026.1 | AC136475.3 | 2.2909 | 10.26478 | 4.22E-12 | 2.06E-11 |
| ENSG00000205885.3 | C1RL-AS1 | 1.304654 | 10.45359 | 6.14E-12 | 2.92E-11 |
| ENSG00000262089.1 | AC040977.1 | 1.253796 | 10.43701 | 2.15E-11 | 1.00E-10 |
| ENSG00000267047.1 | AC040977.2 | 1.402835 | 10.78055 | 3.95E-11 | 1.80E-10 |
| ENSG00000250786.1 | SNHG18 | 1.015076 | 11.15104 | 1.59E-10 | 7.07E-10 |
| ENSG00000203497.2 | PDCD4-AS1 | 1.357545 | 10.22464 | 2.17E-10 | 9.63E-10 |
| ENSG00000261269.1 | AC093278.2 | 1.04431 | 10.79404 | 2.25E-10 | 9.89E-10 |
| ENSG00000267705.1 | AC104365.3 | 1.118822 | 10.65123 | 3.75E-10 | 1.62E-09 |
| ENSG00000269707.1 | AC018730.1 | 1.090289 | 10.5159 | 4.77E-10 | 2.04E-09 |
| ENSG00000270055.1 | AC127502.2 | 1.283512 | 10.25993 | 7.37E-10 | 3.11E-09 |
| ENSG00000261170.1 | AC009053.3 | 1.498185 | 10.24855 | 1.17E-09 | 4.87E-09 |
| ENSG00000273179.1 | AC092535.4 | 1.936024 | 10.04716 | 1.75E-09 | 7.11E-09 |
| ENSG00000232079.2 | LINC01697 | 1.168402 | 10.43085 | 2.54E-09 | 1.03E-08 |
| ENSG00000229380.1 | AC147651.2 | 1.443039 | 11.19863 | 3.19E-09 | 1.27E-08 |
| ENSG00000268621.1 | IGFL2-AS1 | 4.617706 | 10.234 | 4.18E-09 | 1.64E-08 |
| ENSG00000234449.2 | ENSG00000234449 | 1.756165 | 10.55791 | 5.70E-09 | 2.20E-08 |
| ENSG00000272993.1 | ENSG00000272993 | 1.297945 | 10.15102 | 5.89E-09 | 2.25E-08 |
| ENSG00000223799.1 | IL10RB-DT | 1.380288 | 10.05176 | 8.85E-09 | 3.33E-08 |
| ENSG00000254109.1 | RBPMS-AS1 | 1.068147 | 10.59031 | 9.23E-09 | 3.44E-08 |
| ENSG00000261971.2 | MMP25-AS1 | 1.449594 | 9.987822 | 1.03E-08 | 3.81E-08 |
| ENSG00000267010.1 | ENSG00000267010 | 1.053317 | 10.43263 | 2.06E-08 | 7.53E-08 |
| ENSG00000272065.1 | ENSG00000272065 | 1.438558 | 10.00451 | 2.82E-08 | 1.02E-07 |
| ENSG00000270346.1 | ENSG00000270346 | 1.296569 | 10.12795 | 5.38E-08 | 1.92E-07 |
| ENSG00000273102.1 | AP000569.1 | 1.402424 | 10.02818 | 1.03E-07 | 3.61E-07 |
| ENSG00000260552.1 | AC023043.1 | 1.033346 | 10.56394 | 1.04E-07 | 3.65E-07 |
| ENSG00000264868.1 | ENSG00000264868 | 1.379692 | 10.00803 | 1.16E-07 | 4.03E-07 |
| ENSG00000267745.1 | AC060766.7 | 1.007714 | 10.37677 | 1.26E-07 | 4.35E-07 |
| ENSG00000249249.1 | AC010226.1 | 1.174309 | 10.13405 | 1.84E-07 | 6.33E-07 |
| ENSG00000267532.2 | MIR497HG | 1.210176 | 10.16469 | 1.89E-07 | 6.44E-07 |
| ENSG00000272917.1 | AC010186.4 | 1.00259 | 10.30848 | 3.09E-07 | 1.04E-06 |
| ENSG00000269486.1 | ERVK9-11 | 1.322003 | 9.963947 | 3.88E-07 | 1.30E-06 |
| ENSG00000258586.1 | LINC02274 | 1.427653 | 11.61931 | 4.03E-07 | 1.34E-06 |
| ENSG00000270580.1 | AC139256.2 | 1.024782 | 10.30322 | 4.24E-07 | 1.39E-06 |
| ENSG00000261040.2 | WFDC21P | 1.966165 | 10.21748 | 7.26E-07 | 2.33E-06 |
| ENSG00000253930.1 | TNFRSF10A-AS1 | 1.276978 | 9.99111 | 8.25E-07 | 2.63E-06 |
| ENSG00000257410.1 | AC089984.1 | 1.255505 | 9.99758 | 1.04E-06 | 3.28E-06 |
| ENSG00000267547.1 | AC060766.4 | 1.124113 | 10.03138 | 2.43E-06 | 7.31E-06 |
| ENSG00000256802.2 | AC022613.1 | 1.001045 | 10.21686 | 5.04E-06 | 1.47E-05 |
| ENSG00000260196.1 | AC124798.1 | 1.124596 | 10.06428 | 5.09E-06 | 1.47E-05 |
| ENSG00000223914.1 | LINC02471 | 1.328308 | 10.8572 | 1.42E-05 | 3.90E-05 |
| ENSG00000261087.1 | AP003469.4 | 1.085814 | 9.97686 | 1.67E-05 | 4.51E-05 |
| ENSG00000259457.1 | AC100826.1 | 1.16215 | 10.00876 | 0.000535 | 0.001251 |

Note: FC, fold change; CPM, count per million; FDR, false discovery rate.
