# Supplementary material for: A cluster of long non-coding RNAs exhibit diagnostic and prognostic values in renal cell carcinoma
Source: Aging (Albany NY). 2019 Nov 14;11(21):9597–615. doi: 10.18632/aging.102407 (PMC6874440; doi:10.18632/aging.102407)
Supplement: Supplementary Table 2 [file aging-11-102407-s003.docx]

Supplementary Table 2. 99 downregulated lncRNAs after differential expression analysis.

| **Gene_symbol** | **Annotation** | **log_2_FC** | **log_2_CPM** | **adjust P Value** | **FDR** |
| --- | --- | --- | --- | --- | --- |
| ENSG00000226950.2 | DANCR | -1.69479 | 12.26865 | 1.17E-63 | 8.42E-61 |
| ENSG00000254528.3 | AP000757.1 | -3.25814 | 11.07632 | 1.12E-62 | 4.04E-60 |
| ENSG00000258548.1 | LINC00645 | -4.07027 | 10.64885 | 5.46E-56 | 1.31E-53 |
| ENSG00000177133.6 | LINC00982 | -4.95595 | 11.01639 | 1.19E-54 | 2.16E-52 |
| ENSG00000273335.1 | AP005432.2 | -5.07141 | 11.10647 | 1.13E-46 | 1.02E-44 |
| ENSG00000255509.2 | ENSG00000255509 | -3.13379 | 10.47496 | 4.19E-43 | 3.03E-41 |
| ENSG00000248517.1 | LINC02437 | -4.72655 | 10.38232 | 6.67E-43 | 4.38E-41 |
| ENSG00000250529.1 | LINC02121 | -5.51822 | 10.85864 | 1.07E-37 | 5.16E-36 |
| ENSG00000233101.6 | HOXB-AS3 | -2.72658 | 10.64563 | 1.29E-37 | 5.83E-36 |
| ENSG00000232352.1 | SEMA3B-AS1 | -3.43929 | 10.34941 | 9.78E-37 | 3.92E-35 |
| ENSG00000232638.1 | AL390294.1 | -3.98281 | 10.15936 | 1.20E-35 | 4.57E-34 |
| ENSG00000259969.1 | AL049838.1 | -2.94083 | 10.86434 | 2.03E-34 | 7.32E-33 |
| ENSG00000229557.1 | LINC00379 | -3.60072 | 10.49881 | 3.19E-33 | 1.00E-31 |
| ENSG00000236524.1 | ENSG00000236524 | -4.09395 | 10.17496 | 5.23E-33 | 1.57E-31 |
| ENSG00000267259.1 | ERVE-1 | -3.7497 | 10.06646 | 7.42E-32 | 1.98E-30 |
| ENSG00000250742.1 | LINC02381 | -1.87297 | 12.25867 | 1.80E-30 | 4.34E-29 |
| ENSG00000263429.3 | TMEM238L | -4.76767 | 10.54546 | 2.05E-30 | 4.79E-29 |
| ENSG00000237707.1 | AL031726.1 | -3.8098 | 10.04856 | 2.19E-30 | 4.95E-29 |
| ENSG00000233154.1 | LINC01762 | -3.58613 | 10.07316 | 5.51E-30 | 1.20E-28 |
| ENSG00000261399.1 | AL031710.1 | -2.64187 | 10.94784 | 3.49E-29 | 7.41E-28 |
| ENSG00000254789.1 | AC073172.1 | -4.17594 | 10.1462 | 1.00E-28 | 2.07E-27 |
| ENSG00000243350.1 | ENSG00000243350 | -3.66058 | 9.986247 | 3.79E-28 | 7.39E-27 |
| ENSG00000257084.1 | MIR200CHG | -3.56004 | 9.959238 | 2.31E-26 | 3.97E-25 |
| ENSG00000227338.1 | AL139280.1 | -4.68229 | 10.48114 | 2.76E-26 | 4.64E-25 |
| ENSG00000263862.1 | LINC01543 | -4.61252 | 10.37516 | 3.13E-26 | 5.05E-25 |
| ENSG00000228055.2 | FAM245A | -3.42888 | 9.993105 | 5.97E-26 | 9.18E-25 |
| ENSG00000226816.2 | AC005082.1 | -2.8671 | 10.55406 | 1.61E-24 | 2.07E-23 |
| ENSG00000249341.1 | AC124017.1 | -4.22593 | 10.08296 | 1.69E-24 | 2.15E-23 |
| ENSG00000261105.1 | LMO7-AS1 | -2.66477 | 10.423 | 1.77E-24 | 2.20E-23 |
| ENSG00000233237.2 | LINC00472 | -2.11876 | 10.40086 | 5.62E-23 | 6.65E-22 |
| ENSG00000233255.1 | ENSG00000233255 | -2.60965 | 10.6196 | 1.32E-22 | 1.54E-21 |
| ENSG00000231324.1 | AP000696.1 | -3.59582 | 9.988911 | 3.79E-22 | 4.34E-21 |
| ENSG00000235366.1 | LINC01055 | -4.46413 | 10.79602 | 9.31E-22 | 1.03E-20 |
| ENSG00000232835.1 | AC107057.1 | -3.84652 | 9.999279 | 2.45E-21 | 2.56E-20 |
| ENSG00000259104.2 | PTCSC3 | -2.51092 | 10.08804 | 2.38E-20 | 2.29E-19 |
| ENSG00000223414.2 | ENSG00000223414 | -3.02762 | 10.07822 | 2.60E-19 | 2.38E-18 |
| ENSG00000233850.1 | AC103563.7 | -2.6065 | 10.02364 | 2.90E-19 | 2.62E-18 |
| ENSG00000261183.1 | SPINT1-AS1 | -1.29466 | 10.94654 | 6.70E-19 | 5.97E-18 |
| ENSG00000262973.1 | ENSG00000262973 | -3.02804 | 10.55915 | 1.85E-18 | 1.54E-17 |
| ENSG00000266968.1 | AC023421.1 | -5.02817 | 10.98481 | 6.29E-18 | 5.16E-17 |
| ENSG00000166770.6 | ZNF667-AS1 | -1.17226 | 11.21323 | 9.76E-18 | 7.74E-17 |
| ENSG00000230027.1 | AC092813.2 | -3.19765 | 9.970656 | 3.08E-16 | 2.18E-15 |
| ENSG00000261888.1 | AC144831.1 | -2.18215 | 10.09236 | 3.36E-16 | 2.35E-15 |
| ENSG00000235663.1 | SAPCD1-AS1 | -1.15825 | 11.01946 | 4.21E-16 | 2.92E-15 |
| ENSG00000235280.2 | MCF2L-AS1 | -2.05877 | 10.11947 | 4.52E-16 | 3.11E-15 |
| ENSG00000259187.1 | AC122108.1 | -1.65139 | 12.99616 | 2.92E-15 | 1.88E-14 |
| ENSG00000229896.2 | AL157373.2 | -2.05964 | 10.07828 | 3.08E-15 | 1.97E-14 |
| ENSG00000258793.1 | AL355102.4 | -2.20451 | 9.959845 | 3.45E-14 | 2.08E-13 |
| ENSG00000234456.3 | MAGI2-AS3 | -1.3867 | 10.83729 | 3.85E-14 | 2.30E-13 |
| ENSG00000229233.1 | AC011891.1 | -1.62932 | 10.60926 | 6.60E-14 | 3.91E-13 |
| ENSG00000226281.2 | AL031123.1 | -2.25328 | 10.43818 | 1.28E-12 | 6.89E-12 |
| ENSG00000255135.3 | AP002360.1 | -1.0251 | 11.00392 | 1.79E-12 | 9.51E-12 |
| ENSG00000262920.1 | AC129507.4 | -3.20888 | 9.965438 | 1.92E-12 | 1.01E-11 |
| ENSG00000272143.1 | FGF14-AS2 | -1.09825 | 10.79816 | 2.95E-12 | 1.47E-11 |
| ENSG00000270100.1 | AC012065.4 | -1.21089 | 10.60916 | 3.07E-12 | 1.52E-11 |
| ENSG00000223985.1 | LINC01874 | -2.81423 | 10.56561 | 4.44E-12 | 2.15E-11 |
| ENSG00000249042.1 | AC008771.1 | -1.49244 | 10.29107 | 4.52E-12 | 2.18E-11 |
| ENSG00000255021.1 | AC093496.1 | -2.63429 | 10.05426 | 1.90E-11 | 8.89E-11 |
| ENSG00000224281.4 | SLC25A5-AS1 | -1.85441 | 9.996721 | 2.39E-11 | 1.11E-10 |
| ENSG00000258616.1 | LINC02303 | -2.90209 | 9.982947 | 2.66E-11 | 1.22E-10 |
| ENSG00000225376.1 | TMEM246-AS1 | -2.02307 | 10.245 | 4.78E-11 | 2.17E-10 |
| ENSG00000237149.4 | ZNF503-AS2 | -1.54964 | 10.14824 | 1.30E-10 | 5.85E-10 |
| ENSG00000259084.2 | AL133467.3 | -2.30834 | 10.13229 | 1.34E-10 | 6.03E-10 |
| ENSG00000267454.1 | ZNF582-AS1 | -1.52351 | 10.13758 | 3.00E-10 | 1.31E-09 |
| ENSG00000224616.1 | RTCA-AS1 | -1.30999 | 10.29821 | 3.76E-10 | 1.62E-09 |
| ENSG00000272933.1 | AL391121.1 | -1.29256 | 10.35839 | 3.91E-10 | 1.68E-09 |
| ENSG00000266903.1 | AC243964.2 | -1.98258 | 10.05265 | 8.20E-10 | 3.44E-09 |
| ENSG00000260396.1 | ENSG00000260396 | -1.17205 | 10.42225 | 1.53E-09 | 6.30E-09 |
| ENSG00000250885.1 | LINC02061 | -2.07683 | 10.10886 | 1.60E-09 | 6.54E-09 |
| ENSG00000196972.6 | SMIM10L2B | -1.40767 | 10.15334 | 1.60E-09 | 6.54E-09 |
| ENSG00000259134.1 | LINC00924 | -1.35375 | 10.67633 | 3.36E-09 | 1.33E-08 |
| ENSG00000223462.2 | AL353801.1 | -1.45481 | 10.09028 | 3.42E-09 | 1.35E-08 |
| ENSG00000273355.1 | AP000894.4 | -1.39851 | 10.15585 | 4.59E-09 | 1.79E-08 |
| ENSG00000231210.2 | LINC01510 | -1.81793 | 11.01291 | 6.85E-09 | 2.60E-08 |
| ENSG00000234771.2 | SLC25A25-AS1 | -1.52955 | 10.01481 | 9.24E-09 | 3.44E-08 |
| ENSG00000253844.1 | AC064807.2 | -1.21453 | 10.27095 | 1.07E-08 | 3.92E-08 |
| ENSG00000236472.1 | AC002401.1 | -1.95003 | 10.24773 | 3.36E-08 | 1.21E-07 |
| ENSG00000269966.1 | AL136164.2 | -1.33452 | 10.11382 | 4.27E-08 | 1.53E-07 |
| ENSG00000248866.1 | USP46-AS1 | -1.07534 | 10.37557 | 6.88E-08 | 2.45E-07 |
| ENSG00000247400.3 | DNAJC3-DT | -1.16001 | 10.26668 | 1.03E-07 | 3.61E-07 |
| ENSG00000257845.1 | LINC02294 | -1.94713 | 11.00669 | 1.25E-07 | 4.34E-07 |
| ENSG00000243220.1 | AC006159.2 | -1.48925 | 10.2298 | 4.07E-07 | 1.34E-06 |
| ENSG00000263004.1 | AC007114.1 | -1.03902 | 10.33916 | 4.71E-07 | 1.53E-06 |
| ENSG00000237943.2 | PRKCQ-AS1 | -1.2185 | 10.15349 | 5.49E-07 | 1.78E-06 |
| ENSG00000272695.1 | GAS6-DT | -1.28772 | 10.02382 | 8.92E-07 | 2.84E-06 |
| ENSG00000260917.1 | AL158212.3 | -1.14006 | 10.11462 | 9.04E-07 | 2.86E-06 |
| ENSG00000253948.1 | AC104986.2 | -1.04887 | 10.18373 | 2.47E-06 | 7.41E-06 |
| ENSG00000238123.1 | MID1IP1-AS1 | -1.15197 | 10.06045 | 3.56E-06 | 1.06E-05 |
| ENSG00000242048.2 | AC093583.1 | -1.23806 | 10.9028 | 4.22E-06 | 1.23E-05 |
| ENSG00000267280.1 | TBX2-AS1 | -1.11213 | 10.09571 | 5.08E-06 | 1.47E-05 |
| ENSG00000231856.1 | AL162377.1 | -1.19127 | 9.990315 | 6.71E-06 | 1.91E-05 |
| ENSG00000273193.1 | ENSG00000273193 | -1.17871 | 9.969545 | 1.14E-05 | 3.16E-05 |
| ENSG00000257647.1 | AC124312.2 | -1.10181 | 10.00348 | 1.41E-05 | 3.88E-05 |
| ENSG00000259001.2 | *AL355075.4* | *-1.13029* | *10.74918* | *1.56E-05* | *4.26E-05* |
| ENSG00000261801.1 | LOXL1-AS1 | -1.00389 | 10.08601 | 3.06E-05 | 8.15E-05 |
| ENSG00000259359.1 | AC012409.1 | -1.0733 | 10.02399 | 3.31E-05 | 8.72E-05 |
| ENSG00000271347.1 | ENSG00000271347 | -1.0171 | 9.983069 | 5.59E-05 | 0.000144 |
| ENSG00000230487.3 | PSMG3-AS1 | -1.0259 | 10.02186 | 7.43E-05 | 0.000189 |
| ENSG00000226733.1 | AL138826.1 | -1.15972 | 11.38493 | 0.003084 | 0.006344 |
