# Supplementary material for: A cluster of long non-coding RNAs exhibit diagnostic and prognostic values in renal cell carcinoma
Source: Aging (Albany NY). 2019 Nov 14;11(21):9597–615. doi: 10.18632/aging.102407 (PMC6874440; doi:10.18632/aging.102407)
Supplement: Supplementary Table 3 [file aging-11-102407-s004.docx]

Supplementary Table 3. The clinicopathological characteristics of 356 patient samples from Xena TCGA KIRC database regarding DFS.

| **Gene_ID** | **DFS / months** | **EVENT** | **Age** | **Grade** | **M** | **N** | **T** | **Gender** | **Stage** |
| --- | --- | --- | --- | --- | --- | --- | --- | --- | --- |
| KIRC-Tumor-TCGA-A3-3306 | 36.79 | 0 | 67 | G3 | M0 | N0 | T1b | male | stage i |
| KIRC-Tumor-TCGA-A3-3307 | 47.17 | 0 | 66 | G3 | M0 | N0 | T3b | male | stage iii |
| KIRC-Tumor-TCGA-A3-3308 | 0.53 | 0 | 77 | G2 | M0 | N0 | T3b | female | stage iii |
| KIRC-Tumor-TCGA-A3-3316 | 49.05 | 0 | 57 | G3 | M0 | NX | T2 | male | stage ii |
| KIRC-Tumor-TCGA-A3-3317 | 31.27 | 1 | 67 | G2 | M0 | N0 | T2 | male | stage ii |
| KIRC-Tumor-TCGA-A3-3319 | 37.12 | 0 | 70 | G2 | M0 | NX | T1b | male | stage i |
| KIRC-Tumor-TCGA-A3-3320 | 49.54 | 0 | 52 | G1 | M0 | NX | T1b | female | stage i |
| KIRC-Tumor-TCGA-A3-3322 | 48.55 | 0 | 51 | G2 | M0 | NX | T1a | male | stage i |
| KIRC-Tumor-TCGA-A3-3323 | 36.33 | 0 | 53 | G1 | M0 | NX | T1b | male | stage i |
| KIRC-Tumor-TCGA-A3-3326 | 37.35 | 0 | 47 | G1 | M0 | NX | T1a | male | stage i |
| KIRC-Tumor-TCGA-A3-3328 | 45.5 | 0 | 79 | G2 | M0 | N0 | T1b | male | stage i |
| KIRC-Tumor-TCGA-A3-3329 | 53.35 | 0 | 75 | G2 | M0 | N0 | T1b | male | stage i |
| KIRC-Tumor-TCGA-A3-3331 | 48.78 | 0 | 86 | G2 | M0 | N0 | T1 | female | stage i |
| KIRC-Tumor-TCGA-A3-3335 | 36.07 | 1 | 41 | G4 | M0 | N0 | T2a | male | stage ii |
| KIRC-Tumor-TCGA-A3-3343 | 31.04 | 0 | 79 | G3 | M0 | N0 | T2 | male | stage ii |
| KIRC-Tumor-TCGA-A3-3347 | 20.04 | 1 | 76 | G2 | M0 | N1 | T1b | female | stage iii |
| KIRC-Tumor-TCGA-A3-3349 | 45.5 | 0 | 34 | G2 | M0 | N0 | T1b | female | stage i |
| KIRC-Tumor-TCGA-A3-3351 | 29.89 | 0 | 42 | G2 | M0 | N0 | T2a | male | stage ii |
| KIRC-Tumor-TCGA-A3-3357 | 88.3 | 0 | 62 | G3 | M0 | N0 | T2 | male | stage ii |
| KIRC-Tumor-TCGA-A3-3358 | 42.94 | 0 | 57 | G2 | M0 | N0 | T1a | female | stage i |
| KIRC-Tumor-TCGA-A3-3359 | 82.26 | 0 | 82 | G2 | M0 | N0 | T1a | female | stage i |
| KIRC-Tumor-TCGA-A3-3362 | 51.22 | 0 | 60 | G2 | M0 | N0 | T1a | female | stage i |
| KIRC-Tumor-TCGA-A3-3363 | 10.48 | 0 | 50 | G2 | M0 | N0 | T2 | male | stage ii |
| KIRC-Tumor-TCGA-A3-3365 | 28.68 | 0 | 46 | G2 | M0 | NX | T1a | male | stage i |
| KIRC-Tumor-TCGA-A3-3367 | 74.57 | 0 | 72 | G3 | M0 | N0 | T1b | male | stage i |
| KIRC-Tumor-TCGA-A3-3370 | 74.7 | 0 | 48 | G2 | M0 | N0 | T1b | female | stage i |
| KIRC-Tumor-TCGA-A3-3372 | 24.15 | 0 | 64 | G2 | M0 | NX | T3 | male | stage iii |
| KIRC-Tumor-TCGA-A3-3373 | 53.25 | 0 | 54 | G3 | M0 | N0 | T1b | female | stage i |
| KIRC-Tumor-TCGA-A3-3374 | 43.17 | 0 | 51 | G2 | M0 | N0 | T1b | female | stage i |
| KIRC-Tumor-TCGA-A3-3378 | 20.7 | 0 | 60 | G3 | M0 | N0 | T1 | male | stage i |
| KIRC-Tumor-TCGA-A3-3380 | 18.63 | 0 | 54 | G2 | M0 | N0 | T1 | male | stage i |
| KIRC-Tumor-TCGA-A3-3382 | 16.89 | 1 | 69 | G3 | M0 | NX | T1b | male | stage i |
| KIRC-Tumor-TCGA-A3-3385 | 65.47 | 0 | 46 | G2 | M0 | N0 | T1a | female | stage i |
| KIRC-Tumor-TCGA-A3-3387 | 20.27 | 0 | 49 | G2 | M0 | N0 | T1a | male | stage i |
| KIRC-Tumor-TCGA-AK-3426 | 8.41 | 1 | 37 | G3 | M0 | N1 | T3a | male | stage iii |
| KIRC-Tumor-TCGA-AK-3427 | 117.71 | 0 | 65 | GX | M0 | N0 | T1a | male | stage i |
| KIRC-Tumor-TCGA-AK-3428 | 122.47 | 0 | 62 | G2 | M0 | N0 | T3b | male | stage iii |
| KIRC-Tumor-TCGA-AK-3429 | 109.33 | 0 | 54 | G2 | M0 | N0 | T2 | female | stage ii |
| KIRC-Tumor-TCGA-AK-3433 | 111.99 | 0 | 48 | GX | M0 | N0 | T2 | female | stage ii |
| KIRC-Tumor-TCGA-AK-3434 | 53.88 | 1 | 72 | G2 | M0 | NX | T1b | male | stage i |
| KIRC-Tumor-TCGA-AK-3436 | 39.06 | 1 | 40 | G2 | M1 | N0 | T2 | male | stage iv |
| KIRC-Tumor-TCGA-AK-3440 | 94.12 | 0 | 58 | G3 | M0 | NX | T1a | male | stage i |
| KIRC-Tumor-TCGA-AK-3443 | 46.75 | 0 | 45 | GX | M0 | N0 | T2 | male | stage ii |
| KIRC-Tumor-TCGA-AK-3444 | 48.32 | 0 | 80 | G2 | M0 | NX | T1b | female | stage i |
| KIRC-Tumor-TCGA-AK-3445 | 78.58 | 0 | 69 | G3 | M0 | NX | T3a | male | stage iii |
| KIRC-Tumor-TCGA-AK-3450 | 58.44 | 0 | 85 | G2 | M0 | N0 | T1a | female | stage i |
| KIRC-Tumor-TCGA-AK-3451 | 94.22 | 0 | 48 | G3 | M0 | N0 | T2 | male | stage ii |
| KIRC-Tumor-TCGA-AK-3454 | 28.71 | 0 | 84 | G3 | M0 | NX | T1b | male | stage i |
| KIRC-Tumor-TCGA-AK-3456 | 37.55 | 0 | 48 | G3 | M0 | N0 | T2 | male | stage ii |
| KIRC-Tumor-TCGA-AK-3458 | 38.37 | 0 | 48 | G3 | M0 | NX | T1b | male | stage i |
| KIRC-Tumor-TCGA-AK-3460 | 82.39 | 0 | 58 | G2 | M0 | NX | T1a | male | stage i |
| KIRC-Tumor-TCGA-AK-3461 | 72.83 | 0 | 72 | G2 | M0 | NX | T1a | male | stage i |
| KIRC-Tumor-TCGA-AK-3465 | 12.12 | 0 | 71 | GX | M0 | NX | T1b | female | stage i |
| KIRC-Tumor-TCGA-B0-4696 | 26.08 | 1 | 58 | G3 | M0 | N0 | T3a | male | stage iii |
| KIRC-Tumor-TCGA-B0-4718 | 25.23 | 1 | 57 | G2 | M0 | NX | T3a | male | stage iii |
| KIRC-Tumor-TCGA-B0-4810 | 6.73 | 1 | 47 | G3 | M0 | N1 | T3a | male | stage iii |
| KIRC-Tumor-TCGA-B0-4811 | 15.05 | 1 | 48 | G3 | M0 | N0 | T3a | male | stage iii |
| KIRC-Tumor-TCGA-B0-4818 | 4.17 | 1 | 68 | G3 | M0 | NX | T2 | female | stage ii |
| KIRC-Tumor-TCGA-B0-4827 | 21.94 | 1 | 77 | G4 | M0 | N0 | T3b | female | stage iii |
| KIRC-Tumor-TCGA-B0-4837 | 39.68 | 1 | 63 | G3 | M0 | N0 | T1b | male | stage i |
| KIRC-Tumor-TCGA-B0-4841 | 3.55 | 1 | 63 | G3 | M1 | NX | T2 | male | stage iv |
| KIRC-Tumor-TCGA-B0-4842 | 8.21 | 1 | 73 | G4 | M0 | N0 | T3a | female | stage iii |
| KIRC-Tumor-TCGA-B0-4844 | 2.04 | 1 | 60 | G3 | M1 | NX | T3a | male | stage iv |
| KIRC-Tumor-TCGA-B0-4845 | 24.57 | 1 | 70 | G2 | M1 | NX | T3a | male | stage iv |
| KIRC-Tumor-TCGA-B0-4846 | 5.35 | 1 | 52 | G2 | M1 | N0 | T3a | male | stage iv |
| KIRC-Tumor-TCGA-B0-4848 | 16.23 | 1 | 54 | G3 | M0 | NX | T3b | male | stage iii |
| KIRC-Tumor-TCGA-B0-4849 | 0.13 | 1 | 51 | G3 | M0 | NX | T3a | male | stage iii |
| KIRC-Tumor-TCGA-B0-4852 | 28.25 | 1 | 78 | G2 | M0 | N0 | T2 | female | stage ii |
| KIRC-Tumor-TCGA-B0-5080 | 3.38 | 1 | 63 | G3 | M1 | N0 | T3a | male | stage iv |
| KIRC-Tumor-TCGA-B0-5081 | 7 | 1 | 79 | G2 | M0 | N0 | T3b | female | stage iii |
| KIRC-Tumor-TCGA-B0-5094 | 2.46 | 1 | 62 | G2 | M1 | N0 | T3b | male | stage iv |
| KIRC-Tumor-TCGA-B0-5097 | 11.79 | 1 | 59 | G2 | M0 | N0 | T3b | female | stage iii |
| KIRC-Tumor-TCGA-B0-5100 | 46.85 | 1 | 72 | G3 | M0 | NX | T3a | male | stage iii |
| KIRC-Tumor-TCGA-B0-5102 | 89.82 | 1 | 74 | G3 | M0 | NX | T1 | female | stage i |
| KIRC-Tumor-TCGA-B0-5107 | 18.04 | 1 | 65 | G4 | M1 | N0 | T2 | female | stage iv |
| KIRC-Tumor-TCGA-B0-5108 | 58.54 | 0 | 54 | G2 | M0 | N0 | T3a | male | stage iii |
| KIRC-Tumor-TCGA-B0-5109 | 2.3 | 1 | 69 | G4 | M0 | N1 | T3b | male | stage iii |
| KIRC-Tumor-TCGA-B0-5110 | 66 | 0 | 71 | G2 | M0 | N0 | T1a | female | stage i |
| KIRC-Tumor-TCGA-B0-5113 | 38.6 | 0 | 69 | G2 | M0 | N0 | T3a | female | stage iii |
| KIRC-Tumor-TCGA-B0-5115 | 2.46 | 1 | 43 | G3 | M1 | N0 | T2 | male | stage iv |
| KIRC-Tumor-TCGA-B0-5116 | 37.09 | 1 | 52 | G3 | M0 | N0 | T3b | male | stage iii |
| KIRC-Tumor-TCGA-B0-5117 | 52.83 | 0 | 40 | G2 | M0 | NX | T1b | male | stage i |
| KIRC-Tumor-TCGA-B0-5119 | 50.99 | 0 | 61 | G2 | M0 | N0 | T1b | female | stage i |
| KIRC-Tumor-TCGA-B0-5120 | 38.4 | 0 | 72 | G2 | M0 | N0 | T1a | female | stage i |
| KIRC-Tumor-TCGA-B0-5121 | 48.78 | 0 | 56 | G2 | M0 | N0 | T1b | male | stage i |
| KIRC-Tumor-TCGA-B0-5399 | 46.35 | 0 | 46 | G2 | M0 | N0 | T1b | male | stage i |
| KIRC-Tumor-TCGA-B0-5400 | 56.93 | 0 | 59 | G4 | M0 | N0 | T3b | female | stage iii |
| KIRC-Tumor-TCGA-B0-5402 | 14.75 | 1 | 64 | G4 | M0 | NX | T4 | male | stage iv |
| KIRC-Tumor-TCGA-B0-5691 | 106.77 | 1 | 66 | G3 | M0 | N0 | T1a | female | stage i |
| KIRC-Tumor-TCGA-B0-5692 | 129.57 | 0 | 66 | G3 | M0 | N0 | T3b | female | stage iii |
| KIRC-Tumor-TCGA-B0-5693 | 133.84 | 0 | 47 | G2 | M0 | NX | T1b | female | stage i |
| KIRC-Tumor-TCGA-B0-5694 | 9.46 | 1 | 71 | G3 | M0 | N0 | T3a | male | stage iii |
| KIRC-Tumor-TCGA-B0-5695 | 70.63 | 0 | 61 | G2 | M0 | N0 | T1b | female | stage i |
| KIRC-Tumor-TCGA-B0-5696 | 56.73 | 1 | 69 | G4 | M0 | N0 | T3a | male | stage iii |
| KIRC-Tumor-TCGA-B0-5697 | 86.4 | 0 | 50 | G2 | M0 | N0 | T1a | male | stage i |
| KIRC-Tumor-TCGA-B0-5698 | 119.28 | 0 | 77 | G3 | M0 | N0 | T1b | male | stage i |
| KIRC-Tumor-TCGA-B0-5700 | 58.8 | 0 | 77 | G2 | M0 | N0 | T1a | male | stage i |
| KIRC-Tumor-TCGA-B0-5703 | 73.78 | 0 | 73 | G3 | M0 | N0 | T1b | male | stage i |
| KIRC-Tumor-TCGA-B0-5705 | 27.66 | 1 | 65 | G2 | M0 | N0 | T1 | female | stage i |
| KIRC-Tumor-TCGA-B0-5709 | 130.55 | 0 | 62 | G3 | M0 | NX | T3a | female | stage iii |
| KIRC-Tumor-TCGA-B0-5710 | 47.54 | 1 | 57 | G2 | M0 | N0 | T1b | male | stage i |
| KIRC-Tumor-TCGA-B0-5711 | 123.72 | 1 | 50 | G3 | M0 | NX | T3b | male | stage iii |
| KIRC-Tumor-TCGA-B0-5712 | 23.82 | 1 | 68 | G3 | M1 | N0 | T2 | female | stage iv |
| KIRC-Tumor-TCGA-B0-5713 | 91.39 | 0 | 75 | G3 | M0 | N0 | T3b | female | stage iii |
| KIRC-Tumor-TCGA-B0-5812 | 125.95 | 0 | 53 | G3 | M0 | NX | T1b | male | stage i |
| KIRC-Tumor-TCGA-B2-3923 | 32.59 | 0 | 59 | G2 | M0 | NX | T2 | male | stage ii |
| KIRC-Tumor-TCGA-B2-3924 | 35.87 | 0 | 73 | G2 | M0 | NX | T1b | male | stage i |
| KIRC-Tumor-TCGA-B2-4099 | 31.93 | 0 | 83 | G3 | M0 | NX | T1a | male | stage i |
| KIRC-Tumor-TCGA-B2-4101 | 21.29 | 0 | 52 | G3 | M0 | NX | T2a | male | stage ii |
| KIRC-Tumor-TCGA-B2-5633 | 31.64 | 0 | 56 | G2 | M0 | N0 | T1b | male | stage i |
| KIRC-Tumor-TCGA-B2-5636 | 30.19 | 0 | 79 | G2 | M0 | NX | T1a | male | stage i |
| KIRC-Tumor-TCGA-B2-5639 | 27.96 | 1 | 46 | G3 | M1 | NX | T3 | male | stage iv |
| KIRC-Tumor-TCGA-B4-5377 | 11.99 | 0 | 68 | G3 | M1 | N0 | T3 | female | stage iv |
| KIRC-Tumor-TCGA-B4-5378 | 5.75 | 0 | 62 | G2 | M0 | N0 | T1 | male | stage i |
| KIRC-Tumor-TCGA-B4-5832 | 5.09 | 0 | 65 | G2 | M0 | N0 | T3b | male | stage iii |
| KIRC-Tumor-TCGA-B4-5834 | 1.25 | 0 | 59 | G1 | M0 | N0 | T1 | male | stage i |
| KIRC-Tumor-TCGA-B4-5835 | 0.53 | 0 | 64 | G2 | M0 | N0 | T1 | female | stage i |
| KIRC-Tumor-TCGA-B4-5836 | 4.63 | 0 | 61 | G2 | M0 | N0 | T1b | female | stage i |
| KIRC-Tumor-TCGA-B4-5843 | 0.36 | 0 | 45 | G2 | M0 | N0 | T1 | male | stage i |
| KIRC-Tumor-TCGA-B4-5844 | 0.23 | 0 | 61 | G1 | M0 | N0 | T2 | female | stage ii |
| KIRC-Tumor-TCGA-B8-4146 | 16.79 | 0 | 41 | G2 | M0 | NX | T1b | female | stage i |
| KIRC-Tumor-TCGA-B8-4148 | 49.93 | 0 | 63 | G3 | M0 | N0 | T1a | female | stage i |
| KIRC-Tumor-TCGA-B8-4151 | 42.67 | 0 | 51 | G2 | M0 | N0 | T3a | female | stage iii |
| KIRC-Tumor-TCGA-B8-4153 | 7.33 | 1 | 74 | G3 | M0 | NX | T3a | male | stage iii |
| KIRC-Tumor-TCGA-B8-4154 | 45.34 | 0 | 73 | G2 | M0 | N0 | T1a | female | stage i |
| KIRC-Tumor-TCGA-B8-4619 | 17.18 | 0 | 58 | G2 | M0 | N0 | T1a | male | stage i |
| KIRC-Tumor-TCGA-B8-4620 | 19.84 | 1 | 70 | G2 | M0 | N0 | T3a | female | stage iii |
| KIRC-Tumor-TCGA-B8-4621 | 25.89 | 0 | 63 | G3 | M0 | N0 | T1b | male | stage i |
| KIRC-Tumor-TCGA-B8-4622 | 40.31 | 1 | 57 | G3 | M1 | N0 | T3a | male | stage iv |
| KIRC-Tumor-TCGA-B8-5158 | 40.01 | 0 | 56 | G4 | M0 | N1 | T3a | male | stage iii |
| KIRC-Tumor-TCGA-B8-5159 | 23.72 | 0 | 61 | G3 | M0 | N0 | T1a | female | stage i |
| KIRC-Tumor-TCGA-B8-5162 | 1.18 | 0 | 62 | G2 | M0 | NX | T2a | male | stage ii |
| KIRC-Tumor-TCGA-B8-5163 | 27 | 0 | 63 | G3 | M0 | N0 | T3a | female | stage iii |
| KIRC-Tumor-TCGA-B8-5164 | 0.85 | 0 | 65 | G3 | M0 | N0 | T3a | male | stage iii |
| KIRC-Tumor-TCGA-B8-5165 | 24.21 | 0 | 43 | G2 | M0 | N0 | T1a | male | stage i |
| KIRC-Tumor-TCGA-B8-5545 | 50.1 | 0 | 42 | G2 | M0 | N0 | T1a | male | stage i |
| KIRC-Tumor-TCGA-B8-5546 | 16.59 | 0 | 38 | G2 | M0 | N0 | T1b | female | stage i |
| KIRC-Tumor-TCGA-B8-5549 | 6.37 | 0 | 53 | G3 | M0 | N0 | T1b | male | stage i |
| KIRC-Tumor-TCGA-B8-5551 | 0.53 | 0 | 65 | G3 | M0 | N0 | T1b | female | stage i |
| KIRC-Tumor-TCGA-B8-5552 | 34.36 | 0 | 41 | G2 | M0 | NX | T1b | female | stage i |
| KIRC-Tumor-TCGA-BP-4158 | 110.94 | 0 | 69 | G2 | M0 | N0 | T1b | male | stage i |
| KIRC-Tumor-TCGA-BP-4159 | 77.27 | 1 | 70 | G2 | M0 | N0 | T1b | male | stage i |
| KIRC-Tumor-TCGA-BP-4160 | 94.65 | 0 | 67 | G2 | M0 | N0 | T3a | male | stage iii |
| KIRC-Tumor-TCGA-BP-4161 | 88.21 | 1 | 74 | G3 | M0 | NX | T1b | male | stage i |
| KIRC-Tumor-TCGA-BP-4162 | 100.99 | 0 | 65 | G2 | M0 | N0 | T1b | female | stage i |
| KIRC-Tumor-TCGA-BP-4163 | 93.27 | 0 | 60 | G3 | M0 | N0 | T3a | female | stage iii |
| KIRC-Tumor-TCGA-BP-4165 | 60.38 | 1 | 64 | G1 | M0 | N0 | T1b | female | stage i |
| KIRC-Tumor-TCGA-BP-4166 | 0.43 | 0 | 69 | G3 | M0 | N0 | T3a | male | stage iii |
| KIRC-Tumor-TCGA-BP-4167 | 89.29 | 0 | 59 | G2 | M0 | NX | T3a | male | stage iii |
| KIRC-Tumor-TCGA-BP-4169 | 2.56 | 1 | 76 | G2 | M0 | N0 | T2 | female | stage ii |
| KIRC-Tumor-TCGA-BP-4173 | 62.19 | 0 | 47 | G3 | M0 | N0 | T2 | male | stage ii |
| KIRC-Tumor-TCGA-BP-4174 | 61.73 | 0 | 49 | G3 | M0 | N0 | T2 | male | stage ii |
| KIRC-Tumor-TCGA-BP-4176 | 64.22 | 0 | 64 | G2 | M0 | NX | T1b | male | stage i |
| KIRC-Tumor-TCGA-BP-4177 | 54.86 | 0 | 65 | G2 | M0 | NX | T1a | male | stage i |
| KIRC-Tumor-TCGA-BP-4325 | 97.37 | 0 | 64 | G2 | M0 | N0 | T1b | female | stage i |
| KIRC-Tumor-TCGA-BP-4326 | 31.47 | 1 | 53 | G2 | M0 | N0 | T1b | female | stage i |
| KIRC-Tumor-TCGA-BP-4329 | 23 | 1 | 75 | G2 | M0 | N0 | T3a | male | stage iii |
| KIRC-Tumor-TCGA-BP-4330 | 62.02 | 0 | 60 | G2 | M0 | N0 | T3a | female | stage iii |
| KIRC-Tumor-TCGA-BP-4332 | 37.22 | 0 | 36 | G2 | M0 | N0 | T3a | male | stage iii |
| KIRC-Tumor-TCGA-BP-4334 | 14.52 | 1 | 56 | G3 | M0 | N0 | T3a | male | stage iii |
| KIRC-Tumor-TCGA-BP-4335 | 14.91 | 1 | 65 | G3 | M1 | N0 | T3a | female | stage iv |
| KIRC-Tumor-TCGA-BP-4338 | 84.49 | 1 | 43 | G3 | M0 | N0 | T1b | male | stage i |
| KIRC-Tumor-TCGA-BP-4342 | 7.88 | 1 | 79 | G3 | M0 | N0 | T2 | male | stage ii |
| KIRC-Tumor-TCGA-BP-4343 | 61.63 | 1 | 64 | G3 | M0 | N0 | T3a | male | stage iii |
| KIRC-Tumor-TCGA-BP-4344 | 54.73 | 0 | 75 | G2 | M0 | NX | T1a | female | stage i |
| KIRC-Tumor-TCGA-BP-4345 | 49.8 | 0 | 62 | G3 | M0 | N0 | T3b | male | stage iii |
| KIRC-Tumor-TCGA-BP-4347 | 44.91 | 0 | 74 | G2 | M0 | NX | T3b | male | stage iii |
| KIRC-Tumor-TCGA-BP-4349 | 12.22 | 0 | 68 | G2 | M0 | NX | T1a | female | stage i |
| KIRC-Tumor-TCGA-BP-4351 | 28.68 | 1 | 51 | G2 | M0 | N0 | T3a | female | stage iii |
| KIRC-Tumor-TCGA-BP-4352 | 8.71 | 1 | 74 | G4 | M1 | N0 | T3b | female | stage iv |
| KIRC-Tumor-TCGA-BP-4354 | 22.5 | 1 | 40 | G4 | M1 | N1 | T4 | male | stage iv |
| KIRC-Tumor-TCGA-BP-4756 | 12.29 | 0 | 62 | G2 | M0 | N0 | T1b | female | stage i |
| KIRC-Tumor-TCGA-BP-4758 | 72.54 | 0 | 40 | G2 | M0 | NX | T1a | male | stage i |
| KIRC-Tumor-TCGA-BP-4759 | 77.92 | 0 | 50 | G2 | M0 | NX | T1a | male | stage i |
| KIRC-Tumor-TCGA-BP-4760 | 47.86 | 1 | 69 | G2 | M0 | NX | T1a | male | stage i |
| KIRC-Tumor-TCGA-BP-4761 | 5.98 | 0 | 57 | G4 | M0 | N1 | T3a | male | stage iii |
| KIRC-Tumor-TCGA-BP-4765 | 71.75 | 0 | 43 | G2 | M0 | NX | T1a | male | stage i |
| KIRC-Tumor-TCGA-BP-4766 | 48.03 | 0 | 43 | G3 | M0 | NX | T1a | female | stage i |
| KIRC-Tumor-TCGA-BP-4768 | 13.14 | 0 | 72 | G2 | M0 | N0 | T1a | female | stage i |
| KIRC-Tumor-TCGA-BP-4769 | 61.63 | 0 | 63 | G2 | M0 | NX | T1a | male | stage i |
| KIRC-Tumor-TCGA-BP-4770 | 6.83 | 1 | 73 | G4 | M0 | N0 | T4 | female | stage iv |
| KIRC-Tumor-TCGA-BP-4774 | 61.93 | 0 | 57 | G2 | M0 | NX | T1a | female | stage i |
| KIRC-Tumor-TCGA-BP-4775 | 60.55 | 0 | 55 | G2 | M0 | NX | T1a | female | stage i |
| KIRC-Tumor-TCGA-BP-4777 | 56.87 | 0 | 46 | G3 | M0 | NX | T1a | male | stage i |
| KIRC-Tumor-TCGA-BP-4781 | 68.33 | 0 | 78 | G3 | M0 | NX | T1a | male | stage i |
| KIRC-Tumor-TCGA-BP-4782 | 11.63 | 0 | 55 | G2 | M0 | NX | T1a | female | stage i |
| KIRC-Tumor-TCGA-BP-4784 | 60.91 | 0 | 67 | G2 | M0 | NX | T1a | female | stage i |
| KIRC-Tumor-TCGA-BP-4787 | 14.49 | 1 | 59 | G4 | M1 | N0 | T3a | female | stage iv |
| KIRC-Tumor-TCGA-BP-4789 | 48.92 | 0 | 48 | G2 | M0 | NX | T1a | male | stage i |
| KIRC-Tumor-TCGA-BP-4795 | 20.37 | 0 | 74 | G2 | M0 | N0 | T1a | female | stage i |
| KIRC-Tumor-TCGA-BP-4797 | 36.37 | 0 | 34 | G3 | M0 | N0 | T3b | male | stage iii |
| KIRC-Tumor-TCGA-BP-4799 | 11.56 | 1 | 70 | G3 | M0 | N0 | T3b | male | stage iii |
| KIRC-Tumor-TCGA-BP-4801 | 36.93 | 0 | 57 | G2 | M0 | NX | T1a | male | stage i |
| KIRC-Tumor-TCGA-BP-4803 | 6.7 | 0 | 79 | G3 | M0 | NX | T3a | male | stage iii |
| KIRC-Tumor-TCGA-BP-4804 | 41.72 | 1 | 59 | G2 | M0 | NX | T1b | male | stage i |
| KIRC-Tumor-TCGA-BP-4807 | 6.93 | 0 | 42 | G3 | M0 | NX | T1a | male | stage i |
| KIRC-Tumor-TCGA-BP-4959 | 87.39 | 0 | 49 | G3 | M0 | NX | T1b | male | stage i |
| KIRC-Tumor-TCGA-BP-4960 | 71.35 | 0 | 46 | G3 | M0 | N0 | T2 | male | stage ii |
| KIRC-Tumor-TCGA-BP-4961 | 63.57 | 0 | 47 | G2 | M0 | NX | T1a | male | stage i |
| KIRC-Tumor-TCGA-BP-4962 | 58.64 | 0 | 58 | G2 | M0 | NX | T2 | male | stage ii |
| KIRC-Tumor-TCGA-BP-4963 | 60.25 | 0 | 63 | G3 | M0 | NX | T1b | male | stage i |
| KIRC-Tumor-TCGA-BP-4964 | 61.17 | 0 | 54 | G2 | M0 | N0 | T1a | female | stage i |
| KIRC-Tumor-TCGA-BP-4967 | 6.73 | 0 | 76 | G2 | M0 | N0 | T3a | male | stage iii |
| KIRC-Tumor-TCGA-BP-4968 | 57.36 | 0 | 40 | G3 | M0 | N0 | T1b | male | stage i |
| KIRC-Tumor-TCGA-BP-4969 | 58.94 | 0 | 63 | G2 | M0 | NX | T1a | female | stage i |
| KIRC-Tumor-TCGA-BP-4970 | 14.22 | 0 | 44 | G3 | M0 | N1 | T1a | male | stage iii |
| KIRC-Tumor-TCGA-BP-4971 | 48.85 | 0 | 40 | G3 | M0 | N0 | T3a | male | stage iii |
| KIRC-Tumor-TCGA-BP-4972 | 49.34 | 0 | 43 | G3 | M0 | NX | T3a | female | stage iii |
| KIRC-Tumor-TCGA-BP-4973 | 45.47 | 0 | 47 | G3 | M0 | NX | T3a | male | stage iii |
| KIRC-Tumor-TCGA-BP-4974 | 1.15 | 1 | 58 | G4 | M1 | N0 | T3a | male | stage iv |
| KIRC-Tumor-TCGA-BP-4975 | 47.08 | 0 | 40 | G3 | M0 | NX | T1b | male | stage i |
| KIRC-Tumor-TCGA-BP-4976 | 53.61 | 0 | 77 | G3 | M0 | NX | T1a | male | stage i |
| KIRC-Tumor-TCGA-BP-4982 | 33.31 | 0 | 42 | G3 | M0 | NX | T1b | male | stage i |
| KIRC-Tumor-TCGA-BP-4983 | 46.42 | 0 | 67 | G4 | M0 | NX | T3a | female | stage iii |
| KIRC-Tumor-TCGA-BP-4985 | 21.58 | 1 | 72 | G4 | M0 | N0 | T3a | male | stage iii |
| KIRC-Tumor-TCGA-BP-4986 | 25.79 | 0 | 75 | G3 | M0 | N0 | T1a | male | stage i |
| KIRC-Tumor-TCGA-BP-4989 | 3.88 | 0 | 58 | G3 | M0 | N0 | T3a | male | stage iii |
| KIRC-Tumor-TCGA-BP-4991 | 46.42 | 0 | 54 | G2 | M0 | NX | T1a | male | stage i |
| KIRC-Tumor-TCGA-BP-4992 | 16.46 | 0 | 66 | G4 | M0 | NX | T1b | male | stage i |
| KIRC-Tumor-TCGA-BP-4993 | 5.81 | 0 | 58 | G3 | M0 | NX | T1a | male | stage i |
| KIRC-Tumor-TCGA-BP-4994 | 42.97 | 0 | 54 | G3 | M0 | NX | T1a | male | stage i |
| KIRC-Tumor-TCGA-BP-4995 | 45.04 | 0 | 68 | G3 | M0 | N0 | T1b | male | stage i |
| KIRC-Tumor-TCGA-BP-4998 | 30.62 | 0 | 49 | G3 | M0 | NX | T1a | male | stage i |
| KIRC-Tumor-TCGA-BP-4999 | 41.59 | 0 | 56 | G2 | M0 | NX | T1a | male | stage i |
| KIRC-Tumor-TCGA-BP-5000 | 18.5 | 0 | 40 | G3 | M0 | NX | T1b | male | stage i |
| KIRC-Tumor-TCGA-BP-5001 | 38.67 | 0 | 43 | G2 | M0 | NX | T1b | female | stage i |
| KIRC-Tumor-TCGA-BP-5004 | 36.99 | 0 | 53 | G3 | M0 | NX | T1a | male | stage i |
| KIRC-Tumor-TCGA-BP-5006 | 27.6 | 0 | 61 | G2 | M0 | N0 | T1a | male | stage i |
| KIRC-Tumor-TCGA-BP-5007 | 37.45 | 0 | 45 | G2 | M0 | N0 | T2 | male | stage ii |
| KIRC-Tumor-TCGA-BP-5008 | 35.18 | 0 | 46 | G2 | M0 | NX | T1a | male | stage i |
| KIRC-Tumor-TCGA-BP-5009 | 16.16 | 1 | 52 | G3 | M0 | NX | T1b | male | stage i |
| KIRC-Tumor-TCGA-BP-5010 | 25.66 | 1 | 63 | G4 | M0 | N0 | T3a | male | stage iii |
| KIRC-Tumor-TCGA-BP-5169 | 6.34 | 0 | 70 | G4 | M0 | N0 | T1b | male | stage i |
| KIRC-Tumor-TCGA-BP-5170 | 79.24 | 0 | 55 | G2 | M0 | NX | T1a | male | stage i |
| KIRC-Tumor-TCGA-BP-5174 | 74.15 | 0 | 45 | G2 | M0 | NX | T1a | female | stage i |
| KIRC-Tumor-TCGA-BP-5175 | 30.62 | 0 | 60 | G3 | M0 | NX | T1a | male | stage i |
| KIRC-Tumor-TCGA-BP-5177 | 9.63 | 0 | 46 | G3 | M0 | NX | T1a | female | stage i |
| KIRC-Tumor-TCGA-BP-5178 | 56.14 | 1 | 71 | G4 | M1 | NX | T3a | male | stage iv |
| KIRC-Tumor-TCGA-BP-5180 | 74.34 | 0 | 53 | G2 | M0 | NX | T1a | male | stage i |
| KIRC-Tumor-TCGA-BP-5181 | 49.11 | 0 | 58 | G2 | M0 | NX | T1b | female | stage i |
| KIRC-Tumor-TCGA-BP-5182 | 38.27 | 0 | 56 | G3 | M0 | N0 | T1a | male | stage i |
| KIRC-Tumor-TCGA-BP-5183 | 15.93 | 1 | 57 | G3 | M0 | NX | T3a | male | stage iii |
| KIRC-Tumor-TCGA-BP-5184 | 37.22 | 0 | 54 | G3 | M0 | NX | T1a | male | stage i |
| KIRC-Tumor-TCGA-BP-5186 | 22.77 | 0 | 50 | G2 | M0 | N0 | T1a | female | stage i |
| KIRC-Tumor-TCGA-BP-5187 | 13.34 | 0 | 54 | G2 | M0 | NX | T1a | male | stage i |
| KIRC-Tumor-TCGA-BP-5189 | 25.66 | 1 | 60 | G4 | M0 | NX | T1b | male | stage i |
| KIRC-Tumor-TCGA-BP-5190 | 33.21 | 0 | 61 | G3 | M0 | NX | T1a | male | stage i |
| KIRC-Tumor-TCGA-BP-5191 | 31.77 | 0 | 79 | G2 | M0 | N0 | T3a | male | stage iii |
| KIRC-Tumor-TCGA-BP-5192 | 23.46 | 0 | 59 | G2 | M0 | NX | T1a | male | stage i |
| KIRC-Tumor-TCGA-BP-5194 | 13.4 | 0 | 39 | G2 | M0 | NX | T1a | male | stage i |
| KIRC-Tumor-TCGA-BP-5195 | 24.61 | 0 | 75 | G2 | M0 | NX | T1a | male | stage i |
| KIRC-Tumor-TCGA-BP-5196 | 33.44 | 0 | 53 | G2 | M0 | NX | T1a | male | stage i |
| KIRC-Tumor-TCGA-BP-5198 | 19.81 | 0 | 72 | G3 | M0 | N0 | T3b | male | stage iii |
| KIRC-Tumor-TCGA-BP-5199 | 44.51 | 0 | 58 | G4 | M0 | N0 | T2 | male | stage ii |
| KIRC-Tumor-TCGA-BP-5201 | -0.62 | 1 | 63 | G4 | M1 | N0 | T3b | male | stage iv |
| KIRC-Tumor-TCGA-BP-5202 | 0.95 | 0 | 75 | G2 | M0 | NX | T3a | male | stage iii |
| KIRC-Tumor-TCGA-CJ-4634 | 114.91 | 0 | 60 | G2 | M0 | NX | T1b | female | stage i |
| KIRC-Tumor-TCGA-CJ-4635 | 46.52 | 0 | 48 | G3 | M0 | NX | T1b | male | stage i |
| KIRC-Tumor-TCGA-CJ-4636 | 63.21 | 0 | 51 | G3 | M0 | N0 | T3a | male | stage iii |
| KIRC-Tumor-TCGA-CJ-4637 | 59.82 | 1 | 52 | G4 | M1 | NX | T2b | female | stage iv |
| KIRC-Tumor-TCGA-CJ-4638 | 2.3 | 1 | 46 | G4 | M1 | N1 | T3a | female | stage iv |
| KIRC-Tumor-TCGA-CJ-4639 | 106.08 | 0 | 49 | G2 | M0 | N0 | T2 | female | stage ii |
| KIRC-Tumor-TCGA-CJ-4640 | 114.32 | 0 | 49 | G4 | M0 | N0 | T3a | male | stage iii |
| KIRC-Tumor-TCGA-CJ-4643 | 58.9 | 0 | 67 | G3 | M0 | N0 | T2b | female | stage ii |
| KIRC-Tumor-TCGA-CJ-4644 | 3.06 | 1 | 48 | G3 | M1 | N0 | T3a | female | stage iv |
| KIRC-Tumor-TCGA-CJ-4868 | 1.22 | 1 | 42 | G3 | M1 | N0 | T3a | male | stage iv |
| KIRC-Tumor-TCGA-CJ-4869 | 57.62 | 1 | 49 | G2 | M0 | N1 | T2 | male | stage iii |
| KIRC-Tumor-TCGA-CJ-4871 | 5.55 | 1 | 63 | G4 | M1 | NX | T3a | male | stage iv |
| KIRC-Tumor-TCGA-CJ-4872 | 47.14 | 0 | 51 | G4 | M0 | N0 | T1b | male | stage i |
| KIRC-Tumor-TCGA-CJ-4873 | 74.21 | 0 | 85 | G3 | M0 | N0 | T3a | female | stage iii |
| KIRC-Tumor-TCGA-CJ-4874 | 75 | 0 | 73 | G3 | M0 | N0 | T1b | female | stage i |
| KIRC-Tumor-TCGA-CJ-4876 | 64.22 | 0 | 57 | G3 | M0 | N0 | T2b | male | stage ii |
| KIRC-Tumor-TCGA-CJ-4878 | 71.81 | 0 | 71 | G2 | M0 | NX | T3a | female | stage iii |
| KIRC-Tumor-TCGA-CJ-4881 | 61.79 | 1 | 41 | G3 | M0 | NX | T3a | male | stage iii |
| KIRC-Tumor-TCGA-CJ-4882 | 61.86 | 0 | 57 | G3 | M0 | NX | T3a | male | stage iii |
| KIRC-Tumor-TCGA-CJ-4884 | 57.79 | 0 | 72 | G3 | M0 | NX | T3a | female | stage iii |
| KIRC-Tumor-TCGA-CJ-4885 | 67.05 | 1 | 64 | G3 | M1 | NX | T3a | male | stage iv |
| KIRC-Tumor-TCGA-CJ-4886 | 64.13 | 0 | 42 | G3 | M0 | NX | T1a | female | stage i |
| KIRC-Tumor-TCGA-CJ-4887 | 10.58 | 1 | 48 | G3 | M1 | NX | T3a | male | stage iv |
| KIRC-Tumor-TCGA-CJ-4888 | 19.97 | 1 | 59 | G4 | M1 | NX | T3a | male | stage iv |
| KIRC-Tumor-TCGA-CJ-4889 | 63.93 | 0 | 63 | G4 | M0 | NX | T1a | female | stage i |
| KIRC-Tumor-TCGA-CJ-4890 | 48.52 | 1 | 72 | G4 | M1 | N0 | T3a | male | stage iv |
| KIRC-Tumor-TCGA-CJ-4892 | 49.97 | 0 | 65 | G2 | M0 | N0 | T1b | female | stage i |
| KIRC-Tumor-TCGA-CJ-4893 | 24.64 | 0 | 76 | G3 | M0 | NX | T1b | female | stage i |
| KIRC-Tumor-TCGA-CJ-4894 | 5.19 | 1 | 58 | G3 | M0 | N0 | T3a | male | stage iii |
| KIRC-Tumor-TCGA-CJ-4895 | 2 | 1 | 62 | G4 | M1 | NX | T3a | male | stage iv |
| KIRC-Tumor-TCGA-CJ-4897 | 46.75 | 1 | 79 | G3 | M0 | NX | T3a | female | stage iii |
| KIRC-Tumor-TCGA-CJ-4899 | 50.2 | 0 | 42 | G2 | M0 | NX | T1b | male | stage i |
| KIRC-Tumor-TCGA-CJ-4902 | 49.93 | 0 | 61 | G3 | M0 | NX | T3a | male | stage iii |
| KIRC-Tumor-TCGA-CJ-4903 | 51.25 | 0 | 50 | G3 | M0 | NX | T1b | male | stage i |
| KIRC-Tumor-TCGA-CJ-4904 | 9.13 | 1 | 60 | G3 | M1 | N0 | T3a | female | stage iv |
| KIRC-Tumor-TCGA-CJ-4905 | 49.15 | 0 | 62 | G2 | M0 | NX | T1a | female | stage i |
| KIRC-Tumor-TCGA-CJ-4907 | 49.24 | 0 | 58 | G3 | M0 | NX | T3b | male | stage iii |
| KIRC-Tumor-TCGA-CJ-4908 | 50.3 | 0 | 38 | G2 | M0 | NX | T1a | male | stage i |
| KIRC-Tumor-TCGA-CJ-4912 | 54.43 | 0 | 61 | G3 | M0 | NX | T2 | male | stage ii |
| KIRC-Tumor-TCGA-CJ-4916 | 45.11 | 0 | 69 | G3 | M0 | NX | T3a | female | stage iii |
| KIRC-Tumor-TCGA-CJ-4918 | 1.08 | 1 | 64 | G4 | M1 | N0 | T3a | male | stage iv |
| KIRC-Tumor-TCGA-CJ-4923 | 0.82 | 1 | 63 | G4 | M1 | NX | T3a | female | stage iv |
| KIRC-Tumor-TCGA-CJ-5671 | 130.98 | 0 | 51 | G3 | M0 | NX | T1a | male | stage i |
| KIRC-Tumor-TCGA-CJ-5676 | 72.9 | 1 | 47 | G3 | M0 | NX | T3b | male | stage iii |
| KIRC-Tumor-TCGA-CJ-5677 | 9.46 | 1 | 54 | G4 | M1 | NX | T3a | female | stage iv |
| KIRC-Tumor-TCGA-CJ-5678 | 13.11 | 1 | 62 | G3 | M1 | N0 | T2b | male | stage iv |
| KIRC-Tumor-TCGA-CJ-5680 | 5.03 | 1 | 65 | G4 | M1 | NX | T3a | female | stage iv |
| KIRC-Tumor-TCGA-CJ-5681 | 0.56 | 1 | 44 | G3 | M1 | NX | T3a | female | stage iv |
| KIRC-Tumor-TCGA-CJ-5684 | 73.29 | 0 | 61 | G2 | M0 | NX | T3a | male | stage iii |
| KIRC-Tumor-TCGA-CJ-5686 | 66.95 | 0 | 59 | G3 | M0 | NX | T1b | female | stage i |
| KIRC-Tumor-TCGA-CJ-6028 | 4.5 | 1 | 58 | G4 | M1 | NX | T3a | male | stage iv |
| KIRC-Tumor-TCGA-CJ-6031 | 62.61 | 0 | 54 | G3 | M0 | NX | T1b | male | stage i |
| KIRC-Tumor-TCGA-CJ-6032 | 119.55 | 0 | 63 | G3 | M0 | NX | T2 | female | stage ii |
| KIRC-Tumor-TCGA-CJ-6033 | 4.73 | 1 | 54 | G4 | M1 | N0 | T3a | female | stage iv |
| KIRC-Tumor-TCGA-CW-5581 | 91.95 | 0 | 44 | G3 | M0 | NX | T1b | male | stage i |
| KIRC-Tumor-TCGA-CW-5583 | 81.77 | 0 | 51 | G2 | M0 | NX | T1a | female | stage i |
| KIRC-Tumor-TCGA-CW-5584 | 3.35 | 1 | 74 | G3 | M0 | N1 | T3b | male | stage iii |
| KIRC-Tumor-TCGA-CW-5587 | 65.8 | 1 | 62 | G2 | M0 | N0 | T3b | female | stage iii |
| KIRC-Tumor-TCGA-CW-5588 | 66.26 | 0 | 78 | G2 | M0 | NX | T1a | female | stage i |
| KIRC-Tumor-TCGA-CW-5590 | 22.08 | 1 | 51 | G3 | M1 | NX | T3a | male | stage iv |
| KIRC-Tumor-TCGA-CW-5591 | 74.61 | 0 | 56 | G2 | M1 | N0 | T3a | male | stage iv |
| KIRC-Tumor-TCGA-CW-6090 | 91.33 | 1 | 68 | G3 | M0 | NX | T1b | male | stage i |
| KIRC-Tumor-TCGA-CW-6093 | 103.35 | 0 | 73 | G1 | M0 | NX | T1a | male | stage i |
| KIRC-Tumor-TCGA-CW-6097 | 15.01 | 1 | 32 | G4 | M0 | NX | T3a | male | stage iii |
| KIRC-Tumor-TCGA-CZ-4853 | 25.43 | 0 | 82 | G2 | M0 | NX | T1a | male | stage i |
| KIRC-Tumor-TCGA-CZ-4856 | 0.59 | 0 | 62 | G2 | M0 | N0 | T1b | female | stage i |
| KIRC-Tumor-TCGA-CZ-4857 | 32.52 | 1 | 56 | G3 | M1 | N0 | T3a | male | stage iv |
| KIRC-Tumor-TCGA-CZ-4858 | 62.81 | 1 | 39 | G4 | M0 | NX | T2 | male | stage ii |
| KIRC-Tumor-TCGA-CZ-4859 | 58.71 | 0 | 59 | G2 | M0 | N0 | T1 | female | stage i |
| KIRC-Tumor-TCGA-CZ-4862 | 107.46 | 0 | 46 | G2 | M0 | NX | T1b | male | stage i |
| KIRC-Tumor-TCGA-CZ-4863 | 63.34 | 0 | 51 | G3 | M0 | N0 | T3b | female | stage iii |
| KIRC-Tumor-TCGA-CZ-4866 | 107.33 | 0 | 79 | G3 | M0 | NX | T1 | female | stage i |
| KIRC-Tumor-TCGA-CZ-5452 | 58.77 | 0 | 69 | G2 | M0 | N0 | T2 | male | stage ii |
| KIRC-Tumor-TCGA-CZ-5454 | 13.9 | 1 | 63 | G2 | M1 | N0 | T2 | male | stage iv |
| KIRC-Tumor-TCGA-CZ-5456 | 31.24 | 1 | 57 | G3 | M0 | N0 | T2 | male | stage ii |
| KIRC-Tumor-TCGA-CZ-5457 | 5.72 | 1 | 62 | G4 | M0 | NX | T3a | male | stage iii |
| KIRC-Tumor-TCGA-CZ-5458 | 91.62 | 0 | 43 | G3 | M0 | NX | T3a | male | stage iii |
| KIRC-Tumor-TCGA-CZ-5460 | 94.38 | 0 | 55 | G2 | M1 | NX | T3b | male | stage iv |
| KIRC-Tumor-TCGA-CZ-5461 | 1.58 | 1 | 52 | G4 | M1 | NX | T1b | male | stage iv |
| KIRC-Tumor-TCGA-CZ-5463 | 21.75 | 0 | 76 | G2 | M0 | NX | T2 | male | stage ii |
| KIRC-Tumor-TCGA-CZ-5464 | 69.91 | 0 | 69 | G2 | M1 | NX | T3b | male | stage iv |
| KIRC-Tumor-TCGA-CZ-5467 | 2.04 | 1 | 86 | G4 | M0 | N0 | T3a | female | stage iii |
| KIRC-Tumor-TCGA-CZ-5469 | 3.61 | 1 | 41 | G2 | M0 | N0 | T2 | male | stage ii |
| KIRC-Tumor-TCGA-CZ-5470 | 12.68 | 0 | 72 | G3 | M0 | N0 | T2 | female | stage ii |
| KIRC-Tumor-TCGA-CZ-5982 | 80.12 | 0 | 59 | G2 | M0 | NX | T1a | female | stage i |
| KIRC-Tumor-TCGA-CZ-5984 | 67.9 | 0 | 51 | G3 | M0 | N0 | T1b | male | stage i |
| KIRC-Tumor-TCGA-CZ-5985 | 65.6 | 0 | 58 | G2 | M0 | N0 | T2 | male | stage ii |
| KIRC-Tumor-TCGA-CZ-5986 | 12.25 | 0 | 61 | G3 | M0 | N0 | T1 | male | stage i |
| KIRC-Tumor-TCGA-CZ-5987 | 12.39 | 1 | 60 | G2 | M1 | NX | T3b | male | stage iv |
| KIRC-Tumor-TCGA-CZ-5988 | 22.77 | 0 | 38 | G2 | M0 | N0 | T1b | male | stage i |
| KIRC-Tumor-TCGA-CZ-5989 | 62.58 | 0 | 60 | G2 | M0 | N0 | T2 | male | stage ii |
| KIRC-Tumor-TCGA-DV-5566 | 45.93 | 0 | 67 | G2 | M0 | NX | T1a | female | stage i |
| KIRC-Tumor-TCGA-DV-5567 | 65.83 | 1 | 40 | G2 | M0 | NX | T1a | female | stage i |
| KIRC-Tumor-TCGA-DV-5568 | 12.16 | 0 | 26 | G2 | M0 | NX | T1a | male | stage i |
| KIRC-Tumor-TCGA-DV-5569 | 11.66 | 0 | 29 | G2 | M0 | NX | T1a | female | stage i |
| KIRC-Tumor-TCGA-DV-5573 | 37.12 | 0 | 41 | G2 | M0 | NX | T1a | male | stage i |
| KIRC-Tumor-TCGA-DV-5574 | 11.73 | 1 | 37 | G2 | M0 | NX | T1a | male | stage i |
| KIRC-Tumor-TCGA-DV-5575 | 56.8 | 0 | 52 | G2 | M0 | NX | T1a | female | stage i |
| KIRC-Tumor-TCGA-EU-5904 | 18.1 | 0 | 47 | G1 | M0 | NX | T1 | female | stage i |
| KIRC-Tumor-TCGA-EU-5906 | 6.77 | 0 | 55 | G2 | M0 | NX | T1b | male | stage i |

Note: DFS, disease-free survival rate.
