# Supplementary material for: A cluster of long non-coding RNAs exhibit diagnostic and prognostic values in renal cell carcinoma
Source: Aging (Albany NY). 2019 Nov 14;11(21):9597–615. doi: 10.18632/aging.102407 (PMC6874440; doi:10.18632/aging.102407)
Supplement: Supplementary Table 4 [file aging-11-102407-s001.docx]

Supplementary Table 4. The clinicopathological characteristics of 445 patient samples from Xena TCGA KIRC database regarding OS.

| **Gene_ID** | **OS / days** | **EVENT** | **Age** | **Grade** | **M** | **N** | **T** | **Gender** | **Stage** |
| --- | --- | --- | --- | --- | --- | --- | --- | --- | --- |
| KIRC-Tumor-TCGA-A3-3306 | 1120 | 0 | 67 | G3 | M0 | N0 | T1b | male | stage i |
| KIRC-Tumor-TCGA-A3-3307 | 1436 | 0 | 66 | G3 | M0 | N0 | T3b | male | stage iii |
| KIRC-Tumor-TCGA-A3-3308 | 16 | 0 | 77 | G2 | M0 | N0 | T3b | female | stage iii |
| KIRC-Tumor-TCGA-A3-3311 | 1191 | 1 | 57 | G2 | M0 | NX | T1 | male | stage i |
| KIRC-Tumor-TCGA-A3-3313 | 735 | 1 | 59 | G3 | M0 | N0 | T1b | male | stage i |
| KIRC-Tumor-TCGA-A3-3316 | 1493 | 0 | 57 | G3 | M0 | NX | T2 | male | stage ii |
| KIRC-Tumor-TCGA-A3-3317 | 1491 | 0 | 67 | G2 | M0 | N0 | T2 | male | stage ii |
| KIRC-Tumor-TCGA-A3-3319 | 1130 | 0 | 70 | G2 | M0 | NX | T1b | male | stage i |
| KIRC-Tumor-TCGA-A3-3320 | 1508 | 0 | 52 | G1 | M0 | NX | T1b | female | stage i |
| KIRC-Tumor-TCGA-A3-3322 | 1478 | 0 | 51 | G2 | M0 | NX | T1a | male | stage i |
| KIRC-Tumor-TCGA-A3-3323 | 1106 | 0 | 53 | G1 | M0 | NX | T1b | male | stage i |
| KIRC-Tumor-TCGA-A3-3325 | 1170 | 1 | 52 | G2 | M0 | NX | T1a | male | stage i |
| KIRC-Tumor-TCGA-A3-3326 | 1137 | 0 | 47 | G1 | M0 | NX | T1a | male | stage i |
| KIRC-Tumor-TCGA-A3-3328 | 1385 | 0 | 79 | G2 | M0 | N0 | T1b | male | stage i |
| KIRC-Tumor-TCGA-A3-3329 | 1624 | 0 | 75 | G2 | M0 | N0 | T1b | male | stage i |
| KIRC-Tumor-TCGA-A3-3331 | 1485 | 0 | 86 | G2 | M0 | N0 | T1 | female | stage i |
| KIRC-Tumor-TCGA-A3-3335 | 1886 | 0 | 41 | G4 | M0 | N0 | T2a | male | stage ii |
| KIRC-Tumor-TCGA-A3-3343 | 945 | 0 | 79 | G3 | M0 | N0 | T2 | male | stage ii |
| KIRC-Tumor-TCGA-A3-3347 | 1610 | 1 | 76 | G2 | M0 | N1 | T1b | female | stage iii |
| KIRC-Tumor-TCGA-A3-3349 | 1385 | 0 | 34 | G2 | M0 | N0 | T1b | female | stage i |
| KIRC-Tumor-TCGA-A3-3351 | 910 | 0 | 42 | G2 | M0 | N0 | T2a | male | stage ii |
| KIRC-Tumor-TCGA-A3-3352 | 561 | 1 | 74 | G3 | M0 | N0 | T3a | male | stage iii |
| KIRC-Tumor-TCGA-A3-3357 | 2688 | 0 | 62 | G3 | M0 | N0 | T2 | male | stage ii |
| KIRC-Tumor-TCGA-A3-3358 | 1307 | 0 | 57 | G2 | M0 | N0 | T1a | female | stage i |
| KIRC-Tumor-TCGA-A3-3359 | 2504 | 0 | 82 | G2 | M0 | N0 | T1a | female | stage i |
| KIRC-Tumor-TCGA-A3-3362 | 1559 | 0 | 60 | G2 | M0 | N0 | T1a | female | stage i |
| KIRC-Tumor-TCGA-A3-3363 | 319 | 0 | 50 | G2 | M0 | N0 | T2 | male | stage ii |
| KIRC-Tumor-TCGA-A3-3365 | 873 | 0 | 46 | G2 | M0 | NX | T1a | male | stage i |
| KIRC-Tumor-TCGA-A3-3367 | 2270 | 0 | 72 | G3 | M0 | N0 | T1b | male | stage i |
| KIRC-Tumor-TCGA-A3-3370 | 2274 | 0 | 48 | G2 | M0 | N0 | T1b | female | stage i |
| KIRC-Tumor-TCGA-A3-3372 | 735 | 0 | 64 | G2 | M0 | NX | T3 | male | stage iii |
| KIRC-Tumor-TCGA-A3-3373 | 1621 | 0 | 54 | G3 | M0 | N0 | T1b | female | stage i |
| KIRC-Tumor-TCGA-A3-3374 | 1314 | 0 | 51 | G2 | M0 | N0 | T1b | female | stage i |
| KIRC-Tumor-TCGA-A3-3376 | 1696 | 1 | 51 | G2 | M0 | N0 | T1a | male | stage i |
| KIRC-Tumor-TCGA-A3-3378 | 630 | 0 | 60 | G3 | M0 | N0 | T1 | male | stage i |
| KIRC-Tumor-TCGA-A3-3380 | 567 | 0 | 54 | G2 | M0 | N0 | T1 | male | stage i |
| KIRC-Tumor-TCGA-A3-3382 | 574 | 0 | 69 | G3 | M0 | NX | T1b | male | stage i |
| KIRC-Tumor-TCGA-A3-3385 | 1993 | 0 | 46 | G2 | M0 | N0 | T1a | female | stage i |
| KIRC-Tumor-TCGA-A3-3387 | 617 | 0 | 49 | G2 | M0 | N0 | T1a | male | stage i |
| KIRC-Tumor-TCGA-AK-3426 | 885 | 1 | 37 | G3 | M0 | N1 | T3a | male | stage iii |
| KIRC-Tumor-TCGA-AK-3427 | 3583 | 0 | 65 | GX | M0 | N0 | T1a | male | stage i |
| KIRC-Tumor-TCGA-AK-3428 | 3728 | 0 | 62 | G2 | M0 | N0 | T3b | male | stage iii |
| KIRC-Tumor-TCGA-AK-3429 | 3328 | 0 | 54 | G2 | M0 | N0 | T2 | female | stage ii |
| KIRC-Tumor-TCGA-AK-3431 | 2241 | 1 | 62 | G3 | M0 | NX | T2 | female | stage ii |
| KIRC-Tumor-TCGA-AK-3433 | 3409 | 0 | 48 | GX | M0 | N0 | T2 | female | stage ii |
| KIRC-Tumor-TCGA-AK-3434 | 2087 | 0 | 72 | G2 | M0 | NX | T1b | male | stage i |
| KIRC-Tumor-TCGA-AK-3436 | 3331 | 0 | 40 | G2 | M1 | N0 | T2 | male | stage iv |
| KIRC-Tumor-TCGA-AK-3440 | 2865 | 0 | 58 | G3 | M0 | NX | T1a | male | stage i |
| KIRC-Tumor-TCGA-AK-3443 | 1423 | 0 | 45 | GX | M0 | N0 | T2 | male | stage ii |
| KIRC-Tumor-TCGA-AK-3444 | 1471 | 0 | 80 | G2 | M0 | NX | T1b | female | stage i |
| KIRC-Tumor-TCGA-AK-3445 | 2392 | 0 | 69 | G3 | M0 | NX | T3a | male | stage iii |
| KIRC-Tumor-TCGA-AK-3450 | 1779 | 0 | 85 | G2 | M0 | N0 | T1a | female | stage i |
| KIRC-Tumor-TCGA-AK-3451 | 2868 | 0 | 48 | G3 | M0 | N0 | T2 | male | stage ii |
| KIRC-Tumor-TCGA-AK-3454 | 874 | 0 | 84 | G3 | M0 | NX | T1b | male | stage i |
| KIRC-Tumor-TCGA-AK-3455 | 683 | 1 | 71 | G3 | M0 | NX | T3b | female | stage iii |
| KIRC-Tumor-TCGA-AK-3456 | 1143 | 0 | 48 | G3 | M0 | N0 | T2 | male | stage ii |
| KIRC-Tumor-TCGA-AK-3458 | 1168 | 0 | 48 | G3 | M0 | NX | T1b | male | stage i |
| KIRC-Tumor-TCGA-AK-3460 | 2508 | 0 | 58 | G2 | M0 | NX | T1a | male | stage i |
| KIRC-Tumor-TCGA-AK-3461 | 2217 | 0 | 72 | G2 | M0 | NX | T1a | male | stage i |
| KIRC-Tumor-TCGA-AK-3465 | 369 | 0 | 71 | GX | M0 | NX | T1b | female | stage i |
| KIRC-Tumor-TCGA-B0-4688 | 101 | 1 | 46 | G4 | M1 | N0 | T4 | male | stage iv |
| KIRC-Tumor-TCGA-B0-4690 | 43 | 1 | 65 | G3 | M1 | N0 | T4 | male | stage iv |
| KIRC-Tumor-TCGA-B0-4691 | 139 | 1 | 55 | G3 | M1 | N0 | T2 | male | stage iv |
| KIRC-Tumor-TCGA-B0-4693 | 77 | 1 | 72 | G4 | M0 | N0 | T3a | female | stage iii |
| KIRC-Tumor-TCGA-B0-4694 | 106 | 1 | 72 | G4 | M0 | NX | T3b | male | stage iii |
| KIRC-Tumor-TCGA-B0-4696 | 866 | 1 | 58 | G3 | M0 | N0 | T3a | male | stage iii |
| KIRC-Tumor-TCGA-B0-4697 | 578 | 1 | 46 | G4 | M1 | NX | T3b | female | stage iv |
| KIRC-Tumor-TCGA-B0-4698 | 42 | 1 | 75 | G4 | M0 | NX | T4 | male | stage iv |
| KIRC-Tumor-TCGA-B0-4699 | 110 | 1 | 74 | G4 | M1 | N0 | T4 | male | stage iv |
| KIRC-Tumor-TCGA-B0-4700 | 1980 | 1 | 60 | G4 | M1 | NX | T4 | male | stage iv |
| KIRC-Tumor-TCGA-B0-4701 | 238 | 1 | 66 | G3 | M1 | N0 | T3a | female | stage iv |
| KIRC-Tumor-TCGA-B0-4703 | 182 | 1 | 51 | G4 | M1 | N0 | T3a | male | stage iv |
| KIRC-Tumor-TCGA-B0-4706 | 65 | 1 | 61 | G4 | M0 | NX | T3a | male | stage iii |
| KIRC-Tumor-TCGA-B0-4707 | 600 | 1 | 63 | G4 | M0 | NX | T3a | male | stage iii |
| KIRC-Tumor-TCGA-B0-4712 | 1337 | 1 | 76 | G3 | M1 | NX | T3a | male | stage iv |
| KIRC-Tumor-TCGA-B0-4713 | 202 | 1 | 76 | G2 | M0 | NX | T3b | female | stage iii |
| KIRC-Tumor-TCGA-B0-4714 | 99 | 1 | 81 | G3 | M1 | NX | T3b | male | stage iv |
| KIRC-Tumor-TCGA-B0-4718 | 1778 | 0 | 57 | G2 | M0 | NX | T3a | male | stage iii |
| KIRC-Tumor-TCGA-B0-4810 | 478 | 1 | 47 | G3 | M0 | N1 | T3a | male | stage iii |
| KIRC-Tumor-TCGA-B0-4811 | 1417 | 1 | 48 | G3 | M0 | N0 | T3a | male | stage iii |
| KIRC-Tumor-TCGA-B0-4813 | 18 | 1 | 68 | G3 | M0 | NX | T3b | male | stage iii |
| KIRC-Tumor-TCGA-B0-4814 | 168 | 1 | 58 | G3 | M1 | N0 | T4 | male | stage iv |
| KIRC-Tumor-TCGA-B0-4815 | 1588 | 1 | 65 | G4 | M0 | NX | T3a | male | stage iii |
| KIRC-Tumor-TCGA-B0-4816 | 1371 | 1 | 49 | G3 | M0 | N0 | T2 | male | stage ii |
| KIRC-Tumor-TCGA-B0-4817 | 1019 | 1 | 81 | G3 | M0 | N0 | T3c | male | stage iii |
| KIRC-Tumor-TCGA-B0-4818 | 510 | 1 | 68 | G3 | M0 | NX | T2 | female | stage ii |
| KIRC-Tumor-TCGA-B0-4819 | 183 | 1 | 60 | G4 | M1 | NX | T3b | female | stage iv |
| KIRC-Tumor-TCGA-B0-4821 | 1230 | 1 | 68 | G3 | M0 | N0 | T3b | female | stage iii |
| KIRC-Tumor-TCGA-B0-4823 | 454 | 1 | 88 | G2 | M0 | N0 | T1a | male | stage i |
| KIRC-Tumor-TCGA-B0-4824 | 1657 | 1 | 49 | G3 | M0 | N0 | T1a | female | stage i |
| KIRC-Tumor-TCGA-B0-4827 | 885 | 1 | 77 | G4 | M0 | N0 | T3b | female | stage iii |
| KIRC-Tumor-TCGA-B0-4828 | 307 | 1 | 79 | G3 | M1 | NX | T2 | male | stage iv |
| KIRC-Tumor-TCGA-B0-4833 | 2386 | 1 | 82 | G2 | M0 | N0 | T1b | female | stage i |
| KIRC-Tumor-TCGA-B0-4834 | 2090 | 1 | 49 | G3 | M0 | N0 | T1a | male | stage i |
| KIRC-Tumor-TCGA-B0-4836 | 1238 | 1 | 61 | G3 | M1 | NX | T3b | male | stage iv |
| KIRC-Tumor-TCGA-B0-4837 | 1378 | 1 | 63 | G3 | M0 | N0 | T1b | male | stage i |
| KIRC-Tumor-TCGA-B0-4838 | 834 | 1 | 69 | G3 | M0 | N0 | T1b | female | stage i |
| KIRC-Tumor-TCGA-B0-4839 | 1639 | 1 | 80 | G2 | M0 | N0 | T1b | female | stage i |
| KIRC-Tumor-TCGA-B0-4841 | 204 | 1 | 63 | G3 | M1 | NX | T2 | male | stage iv |
| KIRC-Tumor-TCGA-B0-4842 | 1724 | 1 | 73 | G4 | M0 | N0 | T3a | female | stage iii |
| KIRC-Tumor-TCGA-B0-4843 | 320 | 1 | 57 | G3 | M0 | N0 | T3a | male | stage iii |
| KIRC-Tumor-TCGA-B0-4844 | 313 | 1 | 60 | G3 | M1 | NX | T3a | male | stage iv |
| KIRC-Tumor-TCGA-B0-4845 | 1986 | 1 | 70 | G2 | M1 | NX | T3a | male | stage iv |
| KIRC-Tumor-TCGA-B0-4846 | 1200 | 1 | 52 | G2 | M1 | N0 | T3a | male | stage iv |
| KIRC-Tumor-TCGA-B0-4847 | 793 | 1 | 60 | G3 | M1 | NX | T3a | male | stage iv |
| KIRC-Tumor-TCGA-B0-4848 | 883 | 1 | 54 | G3 | M0 | NX | T3b | male | stage iii |
| KIRC-Tumor-TCGA-B0-4849 | 69 | 1 | 51 | G3 | M0 | NX | T3a | male | stage iii |
| KIRC-Tumor-TCGA-B0-4852 | 1121 | 1 | 78 | G2 | M0 | N0 | T2 | female | stage ii |
| KIRC-Tumor-TCGA-B0-4945 | 2145 | 1 | 75 | G2 | M0 | N0 | T1a | female | stage i |
| KIRC-Tumor-TCGA-B0-5075 | 637 | 1 | 77 | G2 | M0 | N0 | T3a | female | stage iii |
| KIRC-Tumor-TCGA-B0-5077 | 1317 | 1 | 77 | G3 | M0 | N0 | T1a | male | stage i |
| KIRC-Tumor-TCGA-B0-5080 | 342 | 1 | 63 | G3 | M1 | N0 | T3a | male | stage iv |
| KIRC-Tumor-TCGA-B0-5081 | 362 | 1 | 79 | G2 | M0 | N0 | T3b | female | stage iii |
| KIRC-Tumor-TCGA-B0-5083 | 1045 | 1 | 63 | G3 | M0 | N0 | T1a | male | stage i |
| KIRC-Tumor-TCGA-B0-5085 | 770 | 1 | 76 | G3 | M0 | N0 | T3a | female | stage iii |
| KIRC-Tumor-TCGA-B0-5088 | 563 | 1 | 53 | G3 | M0 | N0 | T1b | male | stage i |
| KIRC-Tumor-TCGA-B0-5092 | 459 | 1 | 53 | G3 | M1 | N0 | T1a | female | stage iv |
| KIRC-Tumor-TCGA-B0-5094 | 333 | 1 | 62 | G2 | M1 | N0 | T3b | male | stage iv |
| KIRC-Tumor-TCGA-B0-5095 | 245 | 1 | 81 | G3 | M0 | N0 | T3a | male | stage iii |
| KIRC-Tumor-TCGA-B0-5096 | 68 | 1 | 72 | GX | M0 | N1 | T3a | female | stage iii |
| KIRC-Tumor-TCGA-B0-5097 | 665 | 0 | 59 | G2 | M0 | N0 | T3b | female | stage iii |
| KIRC-Tumor-TCGA-B0-5098 | 1584 | 1 | 53 | G3 | M0 | NX | T1 | female | stage i |
| KIRC-Tumor-TCGA-B0-5099 | 485 | 1 | 88 | G3 | M0 | NX | T3b | female | stage iii |
| KIRC-Tumor-TCGA-B0-5100 | 1913 | 1 | 72 | G3 | M0 | NX | T3a | male | stage iii |
| KIRC-Tumor-TCGA-B0-5102 | 2764 | 1 | 74 | G3 | M0 | NX | T1 | female | stage i |
| KIRC-Tumor-TCGA-B0-5106 | 1598 | 1 | 64 | G2 | M0 | N0 | T1a | male | stage i |
| KIRC-Tumor-TCGA-B0-5107 | 927 | 1 | 65 | G4 | M1 | N0 | T2 | female | stage iv |
| KIRC-Tumor-TCGA-B0-5108 | 1782 | 0 | 54 | G2 | M0 | N0 | T3a | male | stage iii |
| KIRC-Tumor-TCGA-B0-5109 | 587 | 1 | 69 | G4 | M0 | N1 | T3b | male | stage iii |
| KIRC-Tumor-TCGA-B0-5110 | 2009 | 0 | 71 | G2 | M0 | N0 | T1a | female | stage i |
| KIRC-Tumor-TCGA-B0-5113 | 1175 | 0 | 69 | G2 | M0 | N0 | T3a | female | stage iii |
| KIRC-Tumor-TCGA-B0-5115 | 1604 | 0 | 43 | G3 | M1 | N0 | T2 | male | stage iv |
| KIRC-Tumor-TCGA-B0-5116 | 1274 | 0 | 52 | G3 | M0 | N0 | T3b | male | stage iii |
| KIRC-Tumor-TCGA-B0-5117 | 1608 | 0 | 40 | G2 | M0 | NX | T1b | male | stage i |
| KIRC-Tumor-TCGA-B0-5119 | 1552 | 0 | 61 | G2 | M0 | N0 | T1b | female | stage i |
| KIRC-Tumor-TCGA-B0-5120 | 1169 | 0 | 72 | G2 | M0 | N0 | T1a | female | stage i |
| KIRC-Tumor-TCGA-B0-5121 | 1485 | 0 | 56 | G2 | M0 | N0 | T1b | male | stage i |
| KIRC-Tumor-TCGA-B0-5399 | 1411 | 0 | 46 | G2 | M0 | N0 | T1b | male | stage i |
| KIRC-Tumor-TCGA-B0-5400 | 1733 | 0 | 59 | G4 | M0 | N0 | T3b | female | stage iii |
| KIRC-Tumor-TCGA-B0-5402 | 1290 | 0 | 64 | G4 | M0 | NX | T4 | male | stage iv |
| KIRC-Tumor-TCGA-B0-5691 | 3431 | 0 | 66 | G3 | M0 | N0 | T1a | female | stage i |
| KIRC-Tumor-TCGA-B0-5692 | 3944 | 0 | 66 | G3 | M0 | N0 | T3b | female | stage iii |
| KIRC-Tumor-TCGA-B0-5693 | 4074 | 0 | 47 | G2 | M0 | NX | T1b | female | stage i |
| KIRC-Tumor-TCGA-B0-5694 | 480 | 1 | 71 | G3 | M0 | N0 | T3a | male | stage iii |
| KIRC-Tumor-TCGA-B0-5695 | 2150 | 0 | 61 | G2 | M0 | N0 | T1b | female | stage i |
| KIRC-Tumor-TCGA-B0-5696 | 2609 | 0 | 69 | G4 | M0 | N0 | T3a | male | stage iii |
| KIRC-Tumor-TCGA-B0-5697 | 2630 | 0 | 50 | G2 | M0 | N0 | T1a | male | stage i |
| KIRC-Tumor-TCGA-B0-5698 | 3631 | 0 | 77 | G3 | M0 | N0 | T1b | male | stage i |
| KIRC-Tumor-TCGA-B0-5700 | 1790 | 0 | 77 | G2 | M0 | N0 | T1a | male | stage i |
| KIRC-Tumor-TCGA-B0-5703 | 2246 | 0 | 73 | G3 | M0 | N0 | T1b | male | stage i |
| KIRC-Tumor-TCGA-B0-5705 | 4537 | 0 | 65 | G2 | M0 | N0 | T1 | female | stage i |
| KIRC-Tumor-TCGA-B0-5709 | 3974 | 0 | 62 | G3 | M0 | NX | T3a | female | stage iii |
| KIRC-Tumor-TCGA-B0-5710 | 2430 | 0 | 57 | G2 | M0 | N0 | T1b | male | stage i |
| KIRC-Tumor-TCGA-B0-5711 | 3989 | 0 | 50 | G3 | M0 | NX | T3b | male | stage iii |
| KIRC-Tumor-TCGA-B0-5712 | 2722 | 0 | 68 | G3 | M1 | N0 | T2 | female | stage iv |
| KIRC-Tumor-TCGA-B0-5713 | 2782 | 0 | 75 | G3 | M0 | N0 | T3b | female | stage iii |
| KIRC-Tumor-TCGA-B0-5812 | 3834 | 0 | 53 | G3 | M0 | NX | T1b | male | stage i |
| KIRC-Tumor-TCGA-B2-3923 | 992 | 0 | 59 | G2 | M0 | NX | T2 | male | stage ii |
| KIRC-Tumor-TCGA-B2-3924 | 1092 | 0 | 73 | G2 | M0 | NX | T1b | male | stage i |
| KIRC-Tumor-TCGA-B2-4098 | 51 | 1 | 72 | G2 | M0 | NX | T1b | female | stage i |
| KIRC-Tumor-TCGA-B2-4099 | 972 | 0 | 83 | G3 | M0 | NX | T1a | male | stage i |
| KIRC-Tumor-TCGA-B2-4101 | 648 | 0 | 52 | G3 | M0 | NX | T2a | male | stage ii |
| KIRC-Tumor-TCGA-B2-5633 | 963 | 0 | 56 | G2 | M0 | N0 | T1b | male | stage i |
| KIRC-Tumor-TCGA-B2-5636 | 919 | 0 | 79 | G2 | M0 | NX | T1a | male | stage i |
| KIRC-Tumor-TCGA-B2-5639 | 1003 | 1 | 46 | G3 | M1 | NX | T3 | male | stage iv |
| KIRC-Tumor-TCGA-B4-5377 | 365 | 0 | 68 | G3 | M1 | N0 | T3 | female | stage iv |
| KIRC-Tumor-TCGA-B4-5378 | 175 | 0 | 62 | G2 | M0 | N0 | T1 | male | stage i |
| KIRC-Tumor-TCGA-B4-5832 | 155 | 0 | 65 | G2 | M0 | N0 | T3b | male | stage iii |
| KIRC-Tumor-TCGA-B4-5834 | 26 | 0 | 59 | G1 | M0 | N0 | T1 | male | stage i |
| KIRC-Tumor-TCGA-B4-5835 | 16 | 0 | 64 | G2 | M0 | N0 | T1 | female | stage i |
| KIRC-Tumor-TCGA-B4-5836 | 141 | 0 | 61 | G2 | M0 | N0 | T1b | female | stage i |
| KIRC-Tumor-TCGA-B4-5843 | 11 | 0 | 45 | G2 | M0 | N0 | T1 | male | stage i |
| KIRC-Tumor-TCGA-B4-5844 | 7 | 0 | 61 | G1 | M0 | N0 | T2 | female | stage ii |
| KIRC-Tumor-TCGA-B8-4143 | 709 | 1 | 66 | G3 | M1 | N0 | T3a | female | stage iv |
| KIRC-Tumor-TCGA-B8-4146 | 511 | 0 | 41 | G2 | M0 | NX | T1b | female | stage i |
| KIRC-Tumor-TCGA-B8-4148 | 1520 | 0 | 63 | G3 | M0 | N0 | T1a | female | stage i |
| KIRC-Tumor-TCGA-B8-4151 | 1299 | 0 | 51 | G2 | M0 | N0 | T3a | female | stage iii |
| KIRC-Tumor-TCGA-B8-4153 | 762 | 0 | 74 | G3 | M0 | NX | T3a | male | stage iii |
| KIRC-Tumor-TCGA-B8-4154 | 1380 | 0 | 73 | G2 | M0 | N0 | T1a | female | stage i |
| KIRC-Tumor-TCGA-B8-4619 | 523 | 0 | 58 | G2 | M0 | N0 | T1a | male | stage i |
| KIRC-Tumor-TCGA-B8-4620 | 777 | 0 | 70 | G2 | M0 | N0 | T3a | female | stage iii |
| KIRC-Tumor-TCGA-B8-4621 | 788 | 0 | 63 | G3 | M0 | N0 | T1b | male | stage i |
| KIRC-Tumor-TCGA-B8-4622 | 1525 | 0 | 57 | G3 | M1 | N0 | T3a | male | stage iv |
| KIRC-Tumor-TCGA-B8-5158 | 1218 | 0 | 56 | G4 | M0 | N1 | T3a | male | stage iii |
| KIRC-Tumor-TCGA-B8-5159 | 722 | 0 | 61 | G3 | M0 | N0 | T1a | female | stage i |
| KIRC-Tumor-TCGA-B8-5162 | 36 | 0 | 62 | G2 | M0 | NX | T2a | male | stage ii |
| KIRC-Tumor-TCGA-B8-5163 | 822 | 0 | 63 | G3 | M0 | N0 | T3a | female | stage iii |
| KIRC-Tumor-TCGA-B8-5164 | 26 | 0 | 65 | G3 | M0 | N0 | T3a | male | stage iii |
| KIRC-Tumor-TCGA-B8-5165 | 737 | 0 | 43 | G2 | M0 | N0 | T1a | male | stage i |
| KIRC-Tumor-TCGA-B8-5545 | 1525 | 0 | 42 | G2 | M0 | N0 | T1a | male | stage i |
| KIRC-Tumor-TCGA-B8-5546 | 505 | 0 | 38 | G2 | M0 | N0 | T1b | female | stage i |
| KIRC-Tumor-TCGA-B8-5549 | 194 | 0 | 53 | G3 | M0 | N0 | T1b | male | stage i |
| KIRC-Tumor-TCGA-B8-5551 | 16 | 0 | 65 | G3 | M0 | N0 | T1b | female | stage i |
| KIRC-Tumor-TCGA-B8-5552 | 1046 | 0 | 41 | G2 | M0 | NX | T1b | female | stage i |
| KIRC-Tumor-TCGA-BP-4158 | 3377 | 0 | 69 | G2 | M0 | N0 | T1b | male | stage i |
| KIRC-Tumor-TCGA-BP-4159 | 2601 | 1 | 70 | G2 | M0 | N0 | T1b | male | stage i |
| KIRC-Tumor-TCGA-BP-4160 | 2881 | 0 | 67 | G2 | M0 | N0 | T3a | male | stage iii |
| KIRC-Tumor-TCGA-BP-4161 | 2746 | 0 | 74 | G3 | M0 | NX | T1b | male | stage i |
| KIRC-Tumor-TCGA-BP-4162 | 3074 | 0 | 65 | G2 | M0 | N0 | T1b | female | stage i |
| KIRC-Tumor-TCGA-BP-4163 | 2839 | 0 | 60 | G3 | M0 | N0 | T3a | female | stage iii |
| KIRC-Tumor-TCGA-BP-4164 | 992 | 1 | 51 | G2 | M0 | NX | T3a | female | stage iii |
| KIRC-Tumor-TCGA-BP-4165 | 3037 | 0 | 64 | G1 | M0 | N0 | T1b | female | stage i |
| KIRC-Tumor-TCGA-BP-4166 | 13 | 0 | 69 | G3 | M0 | N0 | T3a | male | stage iii |
| KIRC-Tumor-TCGA-BP-4167 | 2718 | 0 | 59 | G2 | M0 | NX | T3a | male | stage iii |
| KIRC-Tumor-TCGA-BP-4169 | 701 | 1 | 76 | G2 | M0 | N0 | T2 | female | stage ii |
| KIRC-Tumor-TCGA-BP-4170 | 2343 | 1 | 72 | G2 | M0 | N0 | T1b | female | stage i |
| KIRC-Tumor-TCGA-BP-4173 | 1893 | 0 | 47 | G3 | M0 | N0 | T2 | male | stage ii |
| KIRC-Tumor-TCGA-BP-4174 | 1879 | 0 | 49 | G3 | M0 | N0 | T2 | male | stage ii |
| KIRC-Tumor-TCGA-BP-4176 | 1955 | 0 | 64 | G2 | M0 | NX | T1b | male | stage i |
| KIRC-Tumor-TCGA-BP-4177 | 1670 | 0 | 65 | G2 | M0 | NX | T1a | male | stage i |
| KIRC-Tumor-TCGA-BP-4325 | 2964 | 0 | 64 | G2 | M0 | N0 | T1b | female | stage i |
| KIRC-Tumor-TCGA-BP-4326 | 1625 | 1 | 53 | G2 | M0 | N0 | T1b | female | stage i |
| KIRC-Tumor-TCGA-BP-4327 | 109 | 1 | 75 | G2 | M0 | N0 | T2 | female | stage ii |
| KIRC-Tumor-TCGA-BP-4329 | 845 | 1 | 75 | G2 | M0 | N0 | T3a | male | stage iii |
| KIRC-Tumor-TCGA-BP-4330 | 1888 | 0 | 60 | G2 | M0 | N0 | T3a | female | stage iii |
| KIRC-Tumor-TCGA-BP-4332 | 1133 | 0 | 36 | G2 | M0 | N0 | T3a | male | stage iii |
| KIRC-Tumor-TCGA-BP-4334 | 645 | 1 | 56 | G3 | M0 | N0 | T3a | male | stage iii |
| KIRC-Tumor-TCGA-BP-4335 | 475 | 1 | 65 | G3 | M1 | N0 | T3a | female | stage iv |
| KIRC-Tumor-TCGA-BP-4337 | 2 | 1 | 76 | G4 | M0 | N0 | T3b | female | stage iii |
| KIRC-Tumor-TCGA-BP-4338 | 2859 | 0 | 43 | G3 | M0 | N0 | T1b | male | stage i |
| KIRC-Tumor-TCGA-BP-4340 | 562 | 1 | 70 | G2 | M0 | N0 | T1b | female | stage i |
| KIRC-Tumor-TCGA-BP-4341 | 1589 | 1 | 67 | G2 | M0 | NX | T3a | male | stage iii |
| KIRC-Tumor-TCGA-BP-4342 | 2256 | 1 | 79 | G3 | M0 | N0 | T2 | male | stage ii |
| KIRC-Tumor-TCGA-BP-4343 | 1912 | 1 | 64 | G3 | M0 | N0 | T3a | male | stage iii |
| KIRC-Tumor-TCGA-BP-4344 | 1666 | 0 | 75 | G2 | M0 | NX | T1a | female | stage i |
| KIRC-Tumor-TCGA-BP-4345 | 1516 | 0 | 62 | G3 | M0 | N0 | T3b | male | stage iii |
| KIRC-Tumor-TCGA-BP-4346 | 1493 | 1 | 57 | G3 | M0 | N0 | T3b | male | stage iii |
| KIRC-Tumor-TCGA-BP-4347 | 1367 | 0 | 74 | G2 | M0 | NX | T3b | male | stage iii |
| KIRC-Tumor-TCGA-BP-4349 | 372 | 0 | 68 | G2 | M0 | NX | T1a | female | stage i |
| KIRC-Tumor-TCGA-BP-4351 | 970 | 0 | 51 | G2 | M0 | N0 | T3a | female | stage iii |
| KIRC-Tumor-TCGA-BP-4352 | 344 | 1 | 74 | G4 | M1 | N0 | T3b | female | stage iv |
| KIRC-Tumor-TCGA-BP-4353 | 375 | 1 | 61 | G2 | M0 | N0 | T1 | male | stage i |
| KIRC-Tumor-TCGA-BP-4354 | 1034 | 1 | 40 | G4 | M1 | N1 | T4 | male | stage iv |
| KIRC-Tumor-TCGA-BP-4355 | 953 | 1 | 59 | G4 | M0 | NX | T3a | female | stage iii |
| KIRC-Tumor-TCGA-BP-4756 | 374 | 0 | 62 | G2 | M0 | N0 | T1b | female | stage i |
| KIRC-Tumor-TCGA-BP-4758 | 2208 | 0 | 40 | G2 | M0 | NX | T1a | male | stage i |
| KIRC-Tumor-TCGA-BP-4759 | 2372 | 0 | 50 | G2 | M0 | NX | T1a | male | stage i |
| KIRC-Tumor-TCGA-BP-4760 | 2361 | 0 | 69 | G2 | M0 | NX | T1a | male | stage i |
| KIRC-Tumor-TCGA-BP-4761 | 182 | 0 | 57 | G4 | M0 | N1 | T3a | male | stage iii |
| KIRC-Tumor-TCGA-BP-4762 | 1343 | 1 | 42 | G3 | M0 | NX | T1a | male | stage i |
| KIRC-Tumor-TCGA-BP-4763 | 1270 | 1 | 79 | G2 | M0 | NX | T1a | female | stage i |
| KIRC-Tumor-TCGA-BP-4765 | 2184 | 0 | 43 | G2 | M0 | NX | T1a | male | stage i |
| KIRC-Tumor-TCGA-BP-4766 | 1462 | 0 | 43 | G3 | M0 | NX | T1a | female | stage i |
| KIRC-Tumor-TCGA-BP-4768 | 400 | 0 | 72 | G2 | M0 | N0 | T1a | female | stage i |
| KIRC-Tumor-TCGA-BP-4769 | 1876 | 0 | 63 | G2 | M0 | NX | T1a | male | stage i |
| KIRC-Tumor-TCGA-BP-4770 | 329 | 1 | 73 | G4 | M0 | N0 | T4 | female | stage iv |
| KIRC-Tumor-TCGA-BP-4774 | 1885 | 0 | 57 | G2 | M0 | NX | T1a | female | stage i |
| KIRC-Tumor-TCGA-BP-4775 | 1843 | 0 | 55 | G2 | M0 | NX | T1a | female | stage i |
| KIRC-Tumor-TCGA-BP-4777 | 1731 | 0 | 46 | G3 | M0 | NX | T1a | male | stage i |
| KIRC-Tumor-TCGA-BP-4781 | 2080 | 0 | 78 | G3 | M0 | NX | T1a | male | stage i |
| KIRC-Tumor-TCGA-BP-4782 | 354 | 0 | 55 | G2 | M0 | NX | T1a | female | stage i |
| KIRC-Tumor-TCGA-BP-4784 | 1854 | 0 | 67 | G2 | M0 | NX | T1a | female | stage i |
| KIRC-Tumor-TCGA-BP-4787 | 480 | 1 | 59 | G4 | M1 | N0 | T3a | female | stage iv |
| KIRC-Tumor-TCGA-BP-4789 | 1489 | 0 | 48 | G2 | M0 | NX | T1a | male | stage i |
| KIRC-Tumor-TCGA-BP-4790 | 1111 | 1 | 76 | G2 | M0 | NX | T1a | male | stage i |
| KIRC-Tumor-TCGA-BP-4795 | 620 | 0 | 74 | G2 | M0 | N0 | T1a | female | stage i |
| KIRC-Tumor-TCGA-BP-4797 | 1107 | 0 | 34 | G3 | M0 | N0 | T3b | male | stage iii |
| KIRC-Tumor-TCGA-BP-4799 | 1133 | 1 | 70 | G3 | M0 | N0 | T3b | male | stage iii |
| KIRC-Tumor-TCGA-BP-4801 | 1124 | 0 | 57 | G2 | M0 | NX | T1a | male | stage i |
| KIRC-Tumor-TCGA-BP-4803 | 204 | 0 | 79 | G3 | M0 | NX | T3a | male | stage iii |
| KIRC-Tumor-TCGA-BP-4804 | 1459 | 0 | 59 | G2 | M0 | NX | T1b | male | stage i |
| KIRC-Tumor-TCGA-BP-4807 | 211 | 0 | 42 | G3 | M0 | NX | T1a | male | stage i |
| KIRC-Tumor-TCGA-BP-4959 | 2660 | 0 | 49 | G3 | M0 | NX | T1b | male | stage i |
| KIRC-Tumor-TCGA-BP-4960 | 2172 | 0 | 46 | G3 | M0 | N0 | T2 | male | stage ii |
| KIRC-Tumor-TCGA-BP-4961 | 1935 | 0 | 47 | G2 | M0 | NX | T1a | male | stage i |
| KIRC-Tumor-TCGA-BP-4962 | 1785 | 0 | 58 | G2 | M0 | NX | T2 | male | stage ii |
| KIRC-Tumor-TCGA-BP-4963 | 1834 | 0 | 63 | G3 | M0 | NX | T1b | male | stage i |
| KIRC-Tumor-TCGA-BP-4964 | 1862 | 0 | 54 | G2 | M0 | N0 | T1a | female | stage i |
| KIRC-Tumor-TCGA-BP-4967 | 205 | 0 | 76 | G2 | M0 | N0 | T3a | male | stage iii |
| KIRC-Tumor-TCGA-BP-4968 | 1746 | 0 | 40 | G3 | M0 | N0 | T1b | male | stage i |
| KIRC-Tumor-TCGA-BP-4969 | 1794 | 0 | 63 | G2 | M0 | NX | T1a | female | stage i |
| KIRC-Tumor-TCGA-BP-4970 | 433 | 0 | 44 | G3 | M0 | N1 | T1a | male | stage iii |
| KIRC-Tumor-TCGA-BP-4971 | 1487 | 0 | 40 | G3 | M0 | N0 | T3a | male | stage iii |
| KIRC-Tumor-TCGA-BP-4972 | 1502 | 0 | 43 | G3 | M0 | NX | T3a | female | stage iii |
| KIRC-Tumor-TCGA-BP-4973 | 1384 | 0 | 47 | G3 | M0 | NX | T3a | male | stage iii |
| KIRC-Tumor-TCGA-BP-4974 | 211 | 1 | 58 | G4 | M1 | N0 | T3a | male | stage iv |
| KIRC-Tumor-TCGA-BP-4975 | 1433 | 0 | 40 | G3 | M0 | NX | T1b | male | stage i |
| KIRC-Tumor-TCGA-BP-4976 | 1632 | 0 | 77 | G3 | M0 | NX | T1a | male | stage i |
| KIRC-Tumor-TCGA-BP-4981 | 1097 | 1 | 75 | G3 | M0 | NX | T3a | female | stage iii |
| KIRC-Tumor-TCGA-BP-4982 | 1014 | 0 | 42 | G3 | M0 | NX | T1b | male | stage i |
| KIRC-Tumor-TCGA-BP-4983 | 1413 | 0 | 67 | G4 | M0 | NX | T3a | female | stage iii |
| KIRC-Tumor-TCGA-BP-4985 | 952 | 1 | 72 | G4 | M0 | N0 | T3a | male | stage iii |
| KIRC-Tumor-TCGA-BP-4986 | 785 | 0 | 75 | G3 | M0 | N0 | T1a | male | stage i |
| KIRC-Tumor-TCGA-BP-4988 | 828 | 1 | 72 | G2 | M0 | N0 | T1a | male | stage i |
| KIRC-Tumor-TCGA-BP-4989 | 118 | 0 | 58 | G3 | M0 | N0 | T3a | male | stage iii |
| KIRC-Tumor-TCGA-BP-4991 | 1413 | 0 | 54 | G2 | M0 | NX | T1a | male | stage i |
| KIRC-Tumor-TCGA-BP-4992 | 501 | 0 | 66 | G4 | M0 | NX | T1b | male | stage i |
| KIRC-Tumor-TCGA-BP-4993 | 177 | 0 | 58 | G3 | M0 | NX | T1a | male | stage i |
| KIRC-Tumor-TCGA-BP-4994 | 1308 | 0 | 54 | G3 | M0 | NX | T1a | male | stage i |
| KIRC-Tumor-TCGA-BP-4995 | 1371 | 0 | 68 | G3 | M0 | N0 | T1b | male | stage i |
| KIRC-Tumor-TCGA-BP-4998 | 932 | 0 | 49 | G3 | M0 | NX | T1a | male | stage i |
| KIRC-Tumor-TCGA-BP-4999 | 1266 | 0 | 56 | G2 | M0 | NX | T1a | male | stage i |
| KIRC-Tumor-TCGA-BP-5000 | 563 | 0 | 40 | G3 | M0 | NX | T1b | male | stage i |
| KIRC-Tumor-TCGA-BP-5001 | 1177 | 0 | 43 | G2 | M0 | NX | T1b | female | stage i |
| KIRC-Tumor-TCGA-BP-5004 | 1126 | 0 | 53 | G3 | M0 | NX | T1a | male | stage i |
| KIRC-Tumor-TCGA-BP-5006 | 840 | 0 | 61 | G2 | M0 | N0 | T1a | male | stage i |
| KIRC-Tumor-TCGA-BP-5007 | 1140 | 0 | 45 | G2 | M0 | N0 | T2 | male | stage ii |
| KIRC-Tumor-TCGA-BP-5008 | 1071 | 0 | 46 | G2 | M0 | NX | T1a | male | stage i |
| KIRC-Tumor-TCGA-BP-5009 | 1092 | 1 | 52 | G3 | M0 | NX | T1b | male | stage i |
| KIRC-Tumor-TCGA-BP-5010 | 878 | 1 | 63 | G4 | M0 | N0 | T3a | male | stage iii |
| KIRC-Tumor-TCGA-BP-5168 | 1463 | 1 | 75 | G2 | M0 | NX | T1a | male | stage i |
| KIRC-Tumor-TCGA-BP-5169 | 193 | 0 | 70 | G4 | M0 | N0 | T1b | male | stage i |
| KIRC-Tumor-TCGA-BP-5170 | 2412 | 0 | 55 | G2 | M0 | NX | T1a | male | stage i |
| KIRC-Tumor-TCGA-BP-5173 | 62 | 1 | 75 | G2 | M0 | NX | T1a | male | stage i |
| KIRC-Tumor-TCGA-BP-5174 | 2257 | 0 | 45 | G2 | M0 | NX | T1a | female | stage i |
| KIRC-Tumor-TCGA-BP-5175 | 932 | 0 | 60 | G3 | M0 | NX | T1a | male | stage i |
| KIRC-Tumor-TCGA-BP-5176 | 1590 | 1 | 78 | G2 | M0 | NX | T1a | female | stage i |
| KIRC-Tumor-TCGA-BP-5177 | 293 | 0 | 46 | G3 | M0 | NX | T1a | female | stage i |
| KIRC-Tumor-TCGA-BP-5178 | 1912 | 1 | 71 | G4 | M1 | NX | T3a | male | stage iv |
| KIRC-Tumor-TCGA-BP-5180 | 2263 | 0 | 53 | G2 | M0 | NX | T1a | male | stage i |
| KIRC-Tumor-TCGA-BP-5181 | 1495 | 0 | 58 | G2 | M0 | NX | T1b | female | stage i |
| KIRC-Tumor-TCGA-BP-5182 | 1165 | 0 | 56 | G3 | M0 | N0 | T1a | male | stage i |
| KIRC-Tumor-TCGA-BP-5183 | 1291 | 0 | 57 | G3 | M0 | NX | T3a | male | stage iii |
| KIRC-Tumor-TCGA-BP-5184 | 1133 | 0 | 54 | G3 | M0 | NX | T1a | male | stage i |
| KIRC-Tumor-TCGA-BP-5186 | 693 | 0 | 50 | G2 | M0 | N0 | T1a | female | stage i |
| KIRC-Tumor-TCGA-BP-5187 | 406 | 0 | 54 | G2 | M0 | NX | T1a | male | stage i |
| KIRC-Tumor-TCGA-BP-5189 | 822 | 1 | 60 | G4 | M0 | NX | T1b | male | stage i |
| KIRC-Tumor-TCGA-BP-5190 | 1011 | 0 | 61 | G3 | M0 | NX | T1a | male | stage i |
| KIRC-Tumor-TCGA-BP-5191 | 967 | 0 | 79 | G2 | M0 | N0 | T3a | male | stage iii |
| KIRC-Tumor-TCGA-BP-5192 | 714 | 0 | 59 | G2 | M0 | NX | T1a | male | stage i |
| KIRC-Tumor-TCGA-BP-5194 | 408 | 0 | 39 | G2 | M0 | NX | T1a | male | stage i |
| KIRC-Tumor-TCGA-BP-5195 | 749 | 0 | 75 | G2 | M0 | NX | T1a | male | stage i |
| KIRC-Tumor-TCGA-BP-5196 | 1018 | 0 | 53 | G2 | M0 | NX | T1a | male | stage i |
| KIRC-Tumor-TCGA-BP-5198 | 603 | 0 | 72 | G3 | M0 | N0 | T3b | male | stage iii |
| KIRC-Tumor-TCGA-BP-5199 | 1355 | 0 | 58 | G4 | M0 | N0 | T2 | male | stage ii |
| KIRC-Tumor-TCGA-BP-5201 | 951 | 0 | 63 | G4 | M1 | N0 | T3b | male | stage iv |
| KIRC-Tumor-TCGA-BP-5202 | 29 | 0 | 75 | G2 | M0 | NX | T3a | male | stage iii |
| KIRC-Tumor-TCGA-CJ-4634 | 3498 | 0 | 60 | G2 | M0 | NX | T1b | female | stage i |
| KIRC-Tumor-TCGA-CJ-4635 | 1416 | 0 | 48 | G3 | M0 | NX | T1b | male | stage i |
| KIRC-Tumor-TCGA-CJ-4636 | 1924 | 0 | 51 | G3 | M0 | N0 | T3a | male | stage iii |
| KIRC-Tumor-TCGA-CJ-4637 | 2227 | 1 | 52 | G4 | M1 | NX | T2b | female | stage iv |
| KIRC-Tumor-TCGA-CJ-4638 | 431 | 1 | 46 | G4 | M1 | N1 | T3a | female | stage iv |
| KIRC-Tumor-TCGA-CJ-4639 | 3229 | 0 | 49 | G2 | M0 | N0 | T2 | female | stage ii |
| KIRC-Tumor-TCGA-CJ-4640 | 3480 | 0 | 49 | G4 | M0 | N0 | T3a | male | stage iii |
| KIRC-Tumor-TCGA-CJ-4643 | 1793 | 0 | 67 | G3 | M0 | N0 | T2b | female | stage ii |
| KIRC-Tumor-TCGA-CJ-4644 | 336 | 1 | 48 | G3 | M1 | N0 | T3a | female | stage iv |
| KIRC-Tumor-TCGA-CJ-4868 | 646 | 1 | 42 | G3 | M1 | N0 | T3a | male | stage iv |
| KIRC-Tumor-TCGA-CJ-4869 | 2554 | 0 | 49 | G2 | M0 | N1 | T2 | male | stage iii |
| KIRC-Tumor-TCGA-CJ-4871 | 2423 | 0 | 63 | G4 | M1 | NX | T3a | male | stage iv |
| KIRC-Tumor-TCGA-CJ-4872 | 326 | 0 | 51 | G4 | M0 | N0 | T1b | male | stage i |
| KIRC-Tumor-TCGA-CJ-4873 | 2259 | 0 | 85 | G3 | M0 | N0 | T3a | female | stage iii |
| KIRC-Tumor-TCGA-CJ-4874 | 2283 | 0 | 73 | G3 | M0 | N0 | T1b | female | stage i |
| KIRC-Tumor-TCGA-CJ-4876 | 1955 | 0 | 57 | G3 | M0 | N0 | T2b | male | stage ii |
| KIRC-Tumor-TCGA-CJ-4878 | 2186 | 0 | 71 | G2 | M0 | NX | T3a | female | stage iii |
| KIRC-Tumor-TCGA-CJ-4881 | 2014 | 0 | 41 | G3 | M0 | NX | T3a | male | stage iii |
| KIRC-Tumor-TCGA-CJ-4882 | 1883 | 0 | 57 | G3 | M0 | NX | T3a | male | stage iii |
| KIRC-Tumor-TCGA-CJ-4884 | 1759 | 0 | 72 | G3 | M0 | NX | T3a | female | stage iii |
| KIRC-Tumor-TCGA-CJ-4885 | 3451 | 0 | 64 | G3 | M1 | NX | T3a | male | stage iv |
| KIRC-Tumor-TCGA-CJ-4886 | 1952 | 0 | 42 | G3 | M0 | NX | T1a | female | stage i |
| KIRC-Tumor-TCGA-CJ-4887 | 932 | 1 | 48 | G3 | M1 | NX | T3a | male | stage iv |
| KIRC-Tumor-TCGA-CJ-4888 | 1567 | 1 | 59 | G4 | M1 | NX | T3a | male | stage iv |
| KIRC-Tumor-TCGA-CJ-4889 | 1946 | 0 | 63 | G4 | M0 | NX | T1a | female | stage i |
| KIRC-Tumor-TCGA-CJ-4890 | 3519 | 0 | 72 | G4 | M1 | N0 | T3a | male | stage iv |
| KIRC-Tumor-TCGA-CJ-4891 | 819 | 1 | 57 | G4 | M0 | N0 | T3c | female | stage iii |
| KIRC-Tumor-TCGA-CJ-4892 | 1521 | 0 | 65 | G2 | M0 | N0 | T1b | female | stage i |
| KIRC-Tumor-TCGA-CJ-4893 | 750 | 0 | 76 | G3 | M0 | NX | T1b | female | stage i |
| KIRC-Tumor-TCGA-CJ-4894 | 841 | 1 | 58 | G3 | M0 | N0 | T3a | male | stage iii |
| KIRC-Tumor-TCGA-CJ-4895 | 1200 | 1 | 62 | G4 | M1 | NX | T3a | male | stage iv |
| KIRC-Tumor-TCGA-CJ-4897 | 3341 | 0 | 79 | G3 | M0 | NX | T3a | female | stage iii |
| KIRC-Tumor-TCGA-CJ-4899 | 1528 | 0 | 42 | G2 | M0 | NX | T1b | male | stage i |
| KIRC-Tumor-TCGA-CJ-4900 | 1714 | 1 | 69 | G4 | M1 | N1 | T4 | female | stage iv |
| KIRC-Tumor-TCGA-CJ-4902 | 1520 | 0 | 61 | G3 | M0 | NX | T3a | male | stage iii |
| KIRC-Tumor-TCGA-CJ-4903 | 1559 | 0 | 50 | G3 | M0 | NX | T1b | male | stage i |
| KIRC-Tumor-TCGA-CJ-4904 | 3302 | 0 | 60 | G3 | M1 | N0 | T3a | female | stage iv |
| KIRC-Tumor-TCGA-CJ-4905 | 1496 | 0 | 62 | G2 | M0 | NX | T1a | female | stage i |
| KIRC-Tumor-TCGA-CJ-4907 | 1499 | 0 | 58 | G3 | M0 | NX | T3b | male | stage iii |
| KIRC-Tumor-TCGA-CJ-4908 | 1531 | 0 | 38 | G2 | M0 | NX | T1a | male | stage i |
| KIRC-Tumor-TCGA-CJ-4912 | 1657 | 0 | 61 | G3 | M0 | NX | T2 | male | stage ii |
| KIRC-Tumor-TCGA-CJ-4916 | 1373 | 0 | 69 | G3 | M0 | NX | T3a | female | stage iii |
| KIRC-Tumor-TCGA-CJ-4918 | 93 | 1 | 64 | G4 | M1 | N0 | T3a | male | stage iv |
| KIRC-Tumor-TCGA-CJ-4920 | 139 | 1 | 64 | G2 | M0 | NX | T1b | female | stage i |
| KIRC-Tumor-TCGA-CJ-4923 | 572 | 1 | 63 | G4 | M1 | NX | T3a | female | stage iv |
| KIRC-Tumor-TCGA-CJ-5671 | 3987 | 0 | 51 | G3 | M0 | NX | T1a | male | stage i |
| KIRC-Tumor-TCGA-CJ-5676 | 4067 | 0 | 47 | G3 | M0 | NX | T3b | male | stage iii |
| KIRC-Tumor-TCGA-CJ-5677 | 782 | 1 | 54 | G4 | M1 | NX | T3a | female | stage iv |
| KIRC-Tumor-TCGA-CJ-5678 | 574 | 1 | 62 | G3 | M1 | N0 | T2b | male | stage iv |
| KIRC-Tumor-TCGA-CJ-5680 | 768 | 1 | 65 | G4 | M1 | NX | T3a | female | stage iv |
| KIRC-Tumor-TCGA-CJ-5681 | 552 | 1 | 44 | G3 | M1 | NX | T3a | female | stage iv |
| KIRC-Tumor-TCGA-CJ-5684 | 2231 | 0 | 61 | G2 | M0 | NX | T3a | male | stage iii |
| KIRC-Tumor-TCGA-CJ-5686 | 2038 | 0 | 59 | G3 | M0 | NX | T1b | female | stage i |
| KIRC-Tumor-TCGA-CJ-6027 | 3615 | 1 | 77 | G4 | M0 | NX | T1a | male | stage i |
| KIRC-Tumor-TCGA-CJ-6028 | 1625 | 1 | 58 | G4 | M1 | NX | T3a | male | stage iv |
| KIRC-Tumor-TCGA-CJ-6030 | 2299 | 1 | 65 | G3 | M0 | N0 | T1a | male | stage i |
| KIRC-Tumor-TCGA-CJ-6031 | 1906 | 0 | 54 | G3 | M0 | NX | T1b | male | stage i |
| KIRC-Tumor-TCGA-CJ-6032 | 3639 | 0 | 63 | G3 | M0 | NX | T2 | female | stage ii |
| KIRC-Tumor-TCGA-CJ-6033 | 224 | 1 | 54 | G4 | M1 | N0 | T3a | female | stage iv |
| KIRC-Tumor-TCGA-CW-5580 | 1964 | 1 | 73 | G3 | M1 | NX | T3a | female | stage iv |
| KIRC-Tumor-TCGA-CW-5581 | 2799 | 0 | 44 | G3 | M0 | NX | T1b | male | stage i |
| KIRC-Tumor-TCGA-CW-5583 | 2489 | 0 | 51 | G2 | M0 | NX | T1a | female | stage i |
| KIRC-Tumor-TCGA-CW-5584 | 164 | 1 | 74 | G3 | M0 | N1 | T3b | male | stage iii |
| KIRC-Tumor-TCGA-CW-5587 | 2226 | 0 | 62 | G2 | M0 | N0 | T3b | female | stage iii |
| KIRC-Tumor-TCGA-CW-5588 | 2017 | 0 | 78 | G2 | M0 | NX | T1a | female | stage i |
| KIRC-Tumor-TCGA-CW-5590 | 1075 | 1 | 51 | G3 | M1 | NX | T3a | male | stage iv |
| KIRC-Tumor-TCGA-CW-5591 | 2271 | 0 | 56 | G2 | M1 | N0 | T3a | male | stage iv |
| KIRC-Tumor-TCGA-CW-6087 | 41 | 1 | 61 | G4 | M1 | N1 | T3a | male | stage iv |
| KIRC-Tumor-TCGA-CW-6090 | 2552 | 0 | 68 | G3 | M0 | NX | T1b | male | stage i |
| KIRC-Tumor-TCGA-CW-6093 | 3146 | 0 | 73 | G1 | M0 | NX | T1a | male | stage i |
| KIRC-Tumor-TCGA-CW-6097 | 571 | 1 | 32 | G4 | M0 | NX | T3a | male | stage iii |
| KIRC-Tumor-TCGA-CZ-4853 | 774 | 0 | 82 | G2 | M0 | NX | T1a | male | stage i |
| KIRC-Tumor-TCGA-CZ-4854 | 1404 | 1 | 68 | G2 | M0 | N0 | T1b | male | stage i |
| KIRC-Tumor-TCGA-CZ-4856 | 18 | 0 | 62 | G2 | M0 | N0 | T1b | female | stage i |
| KIRC-Tumor-TCGA-CZ-4857 | 1432 | 1 | 56 | G3 | M1 | N0 | T3a | male | stage iv |
| KIRC-Tumor-TCGA-CZ-4858 | 2105 | 1 | 39 | G4 | M0 | NX | T2 | male | stage ii |
| KIRC-Tumor-TCGA-CZ-4859 | 1787 | 0 | 59 | G2 | M0 | N0 | T1 | female | stage i |
| KIRC-Tumor-TCGA-CZ-4860 | 206 | 1 | 60 | G4 | M1 | NX | T4 | male | stage iv |
| KIRC-Tumor-TCGA-CZ-4861 | 446 | 1 | 63 | G2 | M0 | NX | T2 | male | stage ii |
| KIRC-Tumor-TCGA-CZ-4862 | 3271 | 0 | 46 | G2 | M0 | NX | T1b | male | stage i |
| KIRC-Tumor-TCGA-CZ-4863 | 1928 | 0 | 51 | G3 | M0 | N0 | T3b | female | stage iii |
| KIRC-Tumor-TCGA-CZ-4864 | 2830 | 1 | 86 | G3 | M0 | N0 | T2 | male | stage ii |
| KIRC-Tumor-TCGA-CZ-4865 | 166 | 1 | 70 | G2 | M0 | NX | T1a | female | stage i |
| KIRC-Tumor-TCGA-CZ-4866 | 3267 | 0 | 79 | G3 | M0 | NX | T1 | female | stage i |
| KIRC-Tumor-TCGA-CZ-5452 | 1789 | 0 | 69 | G2 | M0 | N0 | T2 | male | stage ii |
| KIRC-Tumor-TCGA-CZ-5453 | 2419 | 1 | 67 | G2 | M0 | NX | T2 | male | stage ii |
| KIRC-Tumor-TCGA-CZ-5454 | 722 | 1 | 63 | G2 | M1 | N0 | T2 | male | stage iv |
| KIRC-Tumor-TCGA-CZ-5455 | 561 | 1 | 63 | G4 | M1 | NX | T3b | male | stage iv |
| KIRC-Tumor-TCGA-CZ-5456 | 2422 | 0 | 57 | G3 | M0 | N0 | T2 | male | stage ii |
| KIRC-Tumor-TCGA-CZ-5457 | 2754 | 0 | 62 | G4 | M0 | NX | T3a | male | stage iii |
| KIRC-Tumor-TCGA-CZ-5458 | 2789 | 0 | 43 | G3 | M0 | NX | T3a | male | stage iii |
| KIRC-Tumor-TCGA-CZ-5460 | 2873 | 0 | 55 | G2 | M1 | NX | T3b | male | stage iv |
| KIRC-Tumor-TCGA-CZ-5461 | 330 | 1 | 52 | G4 | M1 | NX | T1b | male | stage iv |
| KIRC-Tumor-TCGA-CZ-5462 | 311 | 1 | 83 | G3 | M1 | NX | T1b | male | stage iv |
| KIRC-Tumor-TCGA-CZ-5463 | 662 | 0 | 76 | G2 | M0 | NX | T2 | male | stage ii |
| KIRC-Tumor-TCGA-CZ-5464 | 2128 | 0 | 69 | G2 | M1 | NX | T3b | male | stage iv |
| KIRC-Tumor-TCGA-CZ-5465 | 2564 | 1 | 76 | G2 | M0 | NX | T3b | female | stage iii |
| KIRC-Tumor-TCGA-CZ-5467 | 73 | 1 | 86 | G4 | M0 | N0 | T3a | female | stage iii |
| KIRC-Tumor-TCGA-CZ-5468 | 59 | 1 | 84 | G4 | M1 | NX | T3b | male | stage iv |
| KIRC-Tumor-TCGA-CZ-5469 | 946 | 1 | 41 | G2 | M0 | N0 | T2 | male | stage ii |
| KIRC-Tumor-TCGA-CZ-5470 | 386 | 0 | 72 | G3 | M0 | N0 | T2 | female | stage ii |
| KIRC-Tumor-TCGA-CZ-5982 | 2439 | 0 | 59 | G2 | M0 | NX | T1a | female | stage i |
| KIRC-Tumor-TCGA-CZ-5984 | 2067 | 0 | 51 | G3 | M0 | N0 | T1b | male | stage i |
| KIRC-Tumor-TCGA-CZ-5985 | 1997 | 0 | 58 | G2 | M0 | N0 | T2 | male | stage ii |
| KIRC-Tumor-TCGA-CZ-5986 | 373 | 0 | 61 | G3 | M0 | N0 | T1 | male | stage i |
| KIRC-Tumor-TCGA-CZ-5987 | 445 | 1 | 60 | G2 | M1 | NX | T3b | male | stage iv |
| KIRC-Tumor-TCGA-CZ-5988 | 693 | 0 | 38 | G2 | M0 | N0 | T1b | male | stage i |
| KIRC-Tumor-TCGA-CZ-5989 | 1905 | 0 | 60 | G2 | M0 | N0 | T2 | male | stage ii |
| KIRC-Tumor-TCGA-DV-5566 | 1398 | 0 | 67 | G2 | M0 | NX | T1a | female | stage i |
| KIRC-Tumor-TCGA-DV-5567 | 2004 | 0 | 40 | G2 | M0 | NX | T1a | female | stage i |
| KIRC-Tumor-TCGA-DV-5568 | 370 | 0 | 26 | G2 | M0 | NX | T1a | male | stage i |
| KIRC-Tumor-TCGA-DV-5569 | 355 | 0 | 29 | G2 | M0 | NX | T1a | female | stage i |
| KIRC-Tumor-TCGA-DV-5573 | 1130 | 0 | 41 | G2 | M0 | NX | T1a | male | stage i |
| KIRC-Tumor-TCGA-DV-5574 | 2016 | 0 | 37 | G2 | M0 | NX | T1a | male | stage i |
| KIRC-Tumor-TCGA-DV-5575 | 1729 | 0 | 52 | G2 | M0 | NX | T1a | female | stage i |
| KIRC-Tumor-TCGA-DV-5576 | 727 | 1 | 55 | G2 | M0 | NX | T1a | female | stage i |
| KIRC-Tumor-TCGA-EU-5904 | 551 | 0 | 47 | G1 | M0 | NX | T1 | female | stage i |
| KIRC-Tumor-TCGA-EU-5906 | 206 | 0 | 55 | G2 | M0 | NX | T1b | male | stage i |

Note: OS, overall survival rate.
