# Supplementary material for: A cluster of long non-coding RNAs exhibit diagnostic and prognostic values in renal cell carcinoma
Source: Aging (Albany NY). 2019 Nov 14;11(21):9597–615. doi: 10.18632/aging.102407 (PMC6874440; doi:10.18632/aging.102407)
Supplement: Supplementary Tables 5-41 [file aging-11-102407-s005.docx]

Supplementary TABLES

Supplementary Table 5. Univariate and multivariate analysis of IGFL2-AS1 mRNA level and patient survival.

| **Variable** | **Univariate analysis** | | | |  | **Multivariate analysis^###^** | | | | |
| --- | --- | --- | --- | --- | --- | --- | --- | --- | --- | --- |
|  | HR^#^ | **95% CI^##^** | | **P-value** |  | **HR** | **95% CI** | | | **P-value** |
| Disease free survival (n =356) | | |  | | | | | | | |
| IGFL2-AS1 | | | | | | | | | | |
| Low (n = 178) | 2.382 | 1.595-3.558 | | ***0.000*** |  | 2.285 | 1.522- 3.431 | | | ***0.000*** |
| High (n = 178) |  |  |  |  |  |  |  |  |  |  |
| Age (years) | | | | | | | | | | |
| <=60 (n = 193) | 1.326 | 0.912-1.927 | | 0.140 |  |  |  | | |  |
| >60 (n = 163) |  |  |  |  |  |  |  |  |  |  |
| Gender | | | | | | | | | | |
| Female (n = 123) | 1.276 | 0.851-1.914 | | 0.238 |  |  | |  |  | |
| Male (n = 233) |  |  |  |  |  |  |  |  |  |  |
| T | | | | | | | | | | |
| T1+T2 (n = 232) | 4.531 | 3.063-6.703 | | ***0.000*** |  |  | |  |  | |
| T3+T4 (n = 124) |  |  |  |  |  |  |  |  |  |  |
| N | | | | | | | | | | |
| N0 (n = 345) | 5.267 | 2.536-10.939 | | ***0.000*** |  |  |  | | |  |
| N1 (n = 11) |  |  |  |  |  |  |  |  |  |  |
| M | | | | | | | | | | |
| M0 (n = 309) | 8.660 | 5.836-12.850 | | ***0.000*** |  | 4.214 | 2.696- 6.587 | | | ***0.000*** |
| M1 (n = 47) |  |  |  |  |  |  |  |  |  |  |
| Grade | | | | | | | | | | |
| G1+G2 (n = 172) | 2.940 | 1.922-4.497 | | ***0.000*** |  | 1.931 | 1.244- 2.998 | | | ***0.003*** |
| G3+G4 (n = 184) |  |  |  |  |  |  |  |  |  |  |
| Stage | | | | | | | | | | |
| S1+S2 (n = 220) | 6.642 | 4.320-10.212 | | ***0.000*** |  | 3.199 | 1.944- 5.264 | | | ***0.000*** |
| S3+S4 (n = 136) |  |  |  |  |  |  |  |  |  |  |

^#^HR estimated from Cox proportional hazard regression model; ^##^95% CI of the estimated HR; ^###^multivariate models were adjusted for gene, age, gender, T, N, M, grade and stage.

Supplementary Table 6. Univariate and multivariate analysis of IGFL2-AS1 mRNA level and patient survival.

| **Variable** | **Univariate analysis** | | | |  | **Multivariate analysis^###^** | | |
| --- | --- | --- | --- | --- | --- | --- | --- | --- |
|  | **HR^#^** | **95% CI^##^** | | **P-value** |  | **HR** | **95% CI** | **P-value** |
| Overall survival (n =445) | | |  | | | | | |
| IGFL2-AS1 | | | | | | | | |
| Low (n = 223) | 1.911 | 1.384- 2.638 | | ***0.000*** |  | 1.781 | 1.285-2.468 | ***0.001*** |
| High (n = 222) |  |  |  |  |  |  |  |  |
| Age (years) | | | | | | | | |
| <=60 (n = 219) | 1.727 | 1.253-2.380 | | ***0.001*** |  | 1.640 | 1.188-2.264 | ***0.003*** |
| >60 (n = 226) |  |  |  |  |  |  |  |  |
| Gender | | | | | | | | |
| Female (n = 160) | 0.966 | 0.701-1.331 | | 0.832 |  |  | | |
| Male (n = 285) |  |  |  |  |  |  |  |  |
| T | | | | | | | | |
| T1+T2 (n = 275) | 3.210 | 2.332-4.417 | | ***0.000*** |  |  | | |
| T3+T4 (n = 170) |  |  |  |  |  |  |  |  |
| N | | | | | | | | |
| N0 (n = 431) | 3.362 | 1.768-6.392 | | ***0.000*** |  |  |  |  |
| N1 (n = 14) |  |  |  |  |  |  |  |  |
| M | | | | | | | | |
| M0 (n = 374) | 4.334 | 3.132-5.998 | | ***0.000*** |  | 2.413 | 1.647-3.535 | ***0.000*** |
| M1 (n = 71) |  |  |  |  |  |  |  |  |
| Grade | | | | | | | | |
| G1+G2 (n = 160) | 2.423 | 1.711-3.431 | | ***0.000*** |  | 1.556 | 1.073-2.255 | ***0.020*** |
| G3+G4 (n = 285) |  |  |  |  |  |  |  |  |
| Stage | | | | | | | | |
| S1+S2 (n = 259) | 3.800 | 2.722-5.305 | | ***0.000*** |  | 2.074 | 1.382-3.112 | ***0.000*** |
| S3+S4 (n = 186) |  |  |  |  |  |  |  |  |

^#^HR estimated from Cox proportional hazard regression model; ^##^95% CI of the estimated HR; ^###^multivariate models were adjusted for gene, age, gender, T, N, M, grade and stage.

Supplementary Table 7. Univariate and multivariate analysis of AC023043.1 mRNA level and patient survival.

| **Variable** | **Univariate analysis** | | | |  | **Multivariate analysis^###^** | | | | |
| --- | --- | --- | --- | --- | --- | --- | --- | --- | --- | --- |
|  | **HR^#^** | **95% CI^##^** | | **P-value** |  | **HR** | **95% CI** | | | **P-value** |
| Disease free survival (n =356) | | |  | | | | | | | |
| AC023043.1 | | | | | | | | | | |
| Low (n = 178) | 2.799 | 1.853-4.229 | | ***0.000*** |  | 1.569 | 1.019-2.416 | | | ***0.041*** |
| High (n = 178) |  |  |  |  |  |  |  |  |  |  |
| Age (years) | | | | | | | | | | |
| <=60 (n = 193) | 1.326 | 0.912-1.927 | | 0.140 |  |  |  | | |  |
| >60 (n = 163) |  |  |  |  |  |  |  |  |  |  |
| Gender | | | | | | | | | | |
| Female (n = 123) | 1.276 | 0.851-1.914 | | 0.238 |  |  | |  |  | |
| Male (n = 233) |  |  |  |  |  |  |  |  |  |  |
| T | | | | | | | | | | |
| T1+T2 (n = 232) | 4.531 | 3.063-6.703 | | ***0.000*** |  |  | |  |  | |
| T3+T4 (n = 124) |  |  |  |  |  |  |  |  |  |  |
| N | | | | | | | | | | |
| N0 (n = 345) | 5.267 | 2.536-10.939 | | ***0.000*** |  | 2.322 | 1.099-4.907 | | | ***0.027*** |
| N1 (n = 11) |  |  |  |  |  |  |  |  |  |  |
| M | | | | | | | | | | |
| M0 (n = 309) | 8.660 | 5.836-12.850 | | ***0.000*** |  | 3.863 | 2.471-6.040 | | | ***0.000*** |
| M1 (n = 47) |  |  |  |  |  |  |  |  |  |  |
| Grade | | | | | | | | | | |
| G1+G2 (n = 172) | 2.940 | 1.922-4.497 | | ***0.000*** |  | 1.877 | 1.210- 2.911 | | | ***0.005*** |
| G3+G4 (n = 184) |  |  |  |  |  |  |  |  |  |  |
| Stage | | | | | | | | | | |
| S1+S2 (n = 220) | 6.642 | 4.320-10.212 | | ***0.000*** |  | 3.007 | 1.811-4.995 | | | ***0.000*** |
| S3+S4 (n = 136) |  |  |  |  |  |  |  |  |  |  |

^#^HR estimated from Cox proportional hazard regression model; ^##^95% CI of the estimated HR; ^###^multivariate models were adjusted for gene, age, gender, T, N, M, grade and stage.

Supplementary Table 8. Univariate and multivariate analysis of AC023043.1 mRNA level and patient survival.

| **Variable** | **Univariate analysis** | | | |  | **Multivariate analysis^###^** | | |
| --- | --- | --- | --- | --- | --- | --- | --- | --- |
|  | **HR^#^** | **95% CI^##^** | | **P-value** |  | **HR** | **95% CI** | **P-value** |
| Overall survival (n =445) | | |  | | | | | |
| AC023043.1 | | | | | | | | |
| Low (n = 223) | 2.671 | 1.899-3.758 | | ***0.000*** |  | 1.915 | 1.345-2.728 | ***0.000*** |
| High (n = 222) |  |  |  |  |  |  |  |  |
| Age (years) | | | | | | | | |
| <=60 (n = 219) | 1.727 | 1.253-2.380 | | ***0.001*** |  | 1.660 | 1.203-2.291 | ***0.002*** |
| >60 (n = 226) |  |  |  |  |  |  |  |  |
| Gender | | | | | | | | |
| Female (n = 160) | 0.966 | 0.701-1.331 | | 0.832 |  |  | | |
| Male (n = 285) |  |  |  |  |  |  |  |  |
| T | | | | | | | | |
| T1+T2 (n = 275) | 3.210 | 2.332-4.417 | | ***0.000*** |  |  | | |
| T3+T4 (n = 170) |  |  |  |  |  |  |  |  |
| N | | | | | | | | |
| N0 (n = 431) | 3.362 | 1.768-6.392 | | ***0.000*** |  |  |  |  |
| N1 (n = 14) |  |  |  |  |  |  |  |  |
| M | | | | | | | | |
| M0 (n = 374) | 4.334 | 3.132-5.998 | | ***0.000*** |  | 2.269 | 1.550-3.322 | ***0.000*** |
| M1 (n = 71) |  |  |  |  |  |  |  |  |
| Grade | | | | | | | | |
| G1+G2 (n = 160) | 2.423 | 1.711-3.431 | | ***0.000*** |  | 1.477 | 1.023-2.132 | ***0.038*** |
| G3+G4 (n = 285) |  |  |  |  |  |  |  |  |
| Stage | | | | | | | | |
| S1+S2 (n = 259) | 3.800 | 2.722-5.305 | | ***0.000*** |  | 1.974 | 1.314-2.966 | ***0.001*** |
| S3+S4 (n = 186) |  |  |  |  |  |  |  |  |

^#^HR estimated from Cox proportional hazard regression model; ^##^95% CI of the estimated HR; ^###^multivariate models were adjusted for gene, age, gender, T, N, M, grade and stage.

Supplementary Table 9. Univariate and multivariate analysis of AP000439.2 mRNA level and patient survival.

| **Variable** | **Univariate analysis** | | | |  | **Multivariate analysis^###^** | | | | |
| --- | --- | --- | --- | --- | --- | --- | --- | --- | --- | --- |
|  | **HR^#^** | **95% CI^##^** | | **P-value** |  | **HR** | **95% CI** | | | **P-value** |
| Disease free survival (n =356) | | |  | | | | | | | |
| AP000439.2 | | | | | | | | | | |
| Low (n = 178) | 0.320 | 0.212-0.484 | | *0.000* |  | 0.506 | 0.330-0.775 | | | *0.002* |
| High (n = 178) |  |  |  |  |  |  |  |  |  |  |
| Age (years) | | | | | | | | | | |
| <=60 (n = 193) | 1.326 | 0.912-1.927 | | 0.140 |  |  |  | | |  |
| >60 (n = 163) |  |  |  |  |  |  |  |  |  |  |
| Gender | | | | | | | | | | |
| Female (n = 123) | 1.276 | 0.851-1.914 | | 0.238 |  |  | |  |  | |
| Male (n = 233) |  |  |  |  |  |  |  |  |  |  |
| T | | | | | | | | | | |
| T1+T2 (n = 232) | 4.531 | 3.063-6.703 | | *0.000* |  |  | |  |  | |
| T3+T4 (n = 124) |  |  |  |  |  |  |  |  |  |  |
| N | | | | | | | | | | |
| N0 (n = 345) | 5.267 | 2.536-10.939 | | *0.000* |  | 2.418 | 1.151-5.081 | | | *0.020* |
| N1 (n = 11) |  |  |  |  |  |  |  |  |  |  |
| M | | | | | | | | | | |
| M0 (n = 309) | 8.660 | 5.836-12.850 | | *0.000* |  | 3.646 | 2.332-5.699 | | | *0.000* |
| M1 (n = 47) |  |  |  |  |  |  |  |  |  |  |
| Grade | | | | | | | | | | |
| G1+G2 (n = 172) | 2.940 | 1.922-4.497 | | *0.000* |  | 1.830 | 1.176-2.845 | | | *0.007* |
| G3+G4 (n = 184) |  |  |  |  |  |  |  |  |  |  |
| Stage | | | | | | | | | | |
| S1+S2 (n = 220) | 6.642 | 4.320-10.212 | | *0.000* |  | 3.028 | 1.831-5.006 | | | *0.000* |
| S3+S4 (n = 136) |  |  |  |  |  |  |  |  |  |  |

^#^HR estimated from Cox proportional hazard regression model; ^##^95% CI of the estimated HR; ^###^multivariate models were adjusted for gene, age, gender, T, N, M, grade and stage.

Supplementary Table 10. Univariate and multivariate analysis of AP000439.2 mRNA level and patient survival.

| **Variable** | **Univariate analysis** | | | |  | **Multivariate analysis^###^** | | |
| --- | --- | --- | --- | --- | --- | --- | --- | --- |
|  | **HR^#^** | **95% CI^##^** | | **P-value** |  | **HR** | **95% CI** | **P-value** |
| Overall survival (n =445) | | |  | | | | | |
| AP000439.2 | | | | | | | | |
| Low (n = 223) | 0.462 | 0.334-0.639 | | ***0.000*** |  | 0.617 | 0.442-0.861 | ***0.005*** |
| High (n = 222) |  |  |  |  |  |  |  |  |
| Age (years) | | | | | | | | |
| <=60 (n = 219) | 1.727 | 1.253-2.380 | | ***0.001*** |  | 1.622 | 1.174-2.241 | ***0.003*** |
| >60 (n = 226) |  |  |  |  |  |  |  |  |
| Gender | | | | | | | | |
| Female (n = 160) | 0.966 | 0.701-1.331 | | 0.832 |  |  | | |
| Male (n = 285) |  |  |  |  |  |  |  |  |
| T | | | | | | | | |
| T1+T2 (n = 275) | 3.210 | 2.332-4.417 | | ***0.000*** |  |  | | |
| T3+T4 (n = 170) |  |  |  |  |  |  |  |  |
| N | | | | | | | | |
| N0 (n = 431) | 3.362 | 1.768-6.392 | | ***0.000*** |  | 2.093 | 1.086-4.034 | ***0.027*** |
| N1 (n = 14) |  |  |  |  |  |  |  |  |
| M | | | | | | | | |
| M0 (n = 374) | 4.334 | 3.132-5.998 | | ***0.000*** |  | 2.317 | 1.582-3.394 | ***0.000*** |
| M1 (n = 71) |  |  |  |  |  |  |  |  |
| Grade | | | | | | | | |
| G1+G2 (n = 160) | 2.423 | 1.711-3.431 | | ***0.000*** |  | 1.465 | 1.009-2.126 | ***0.045*** |
| G3+G4 (n = 285) |  |  |  |  |  |  |  |  |
| Stage | | | | | | | | |
| S1+S2 (n = 259) | 3.800 | 2.722-5.305 | | ***0.000*** |  | 1.968 | 1.304-2.971 | ***0.001*** |
| S3+S4 (n = 186) |  |  |  |  |  |  |  |  |

^#^HR estimated from Cox proportional hazard regression model; ^##^95% CI of the estimated HR; ^###^multivariate models were adjusted for gene, age, gender, T, N, M, grade and stage.

Supplementary Table 11. Univariate and multivariate analysis of AC124854.1 mRNA level and patient survival.

| **Variable** | **Univariate analysis** | | | |  | **Multivariate analysis^###^** | | | | |
| --- | --- | --- | --- | --- | --- | --- | --- | --- | --- | --- |
|  | **HR^#^** | **95% CI^##^** | | **P-value** |  | **HR** | **95% CI** | | | **P-value** |
| Disease free survival (n =356) | | |  | | | | | | | |
| AC124854.1 | | | | | | | | | | |
| Low (n = 178) | 0.380 | 0.252-0.571 | | ***0.000*** |  | 0.624 | 0.409-0.953 | | | ***0.029*** |
| High (n = 178) |  |  |  |  |  |  |  |  |  |  |
| Age (years) | | | | | | | | | | |
| <=60 (n = 193) | 1.326 | 0.912-1.927 | | 0.140 |  |  |  | | |  |
| >60 (n = 163) |  |  |  |  |  |  |  |  |  |  |
| Gender | | | | | | | | | | |
| Female (n = 123) | 1.276 | 0.851-1.914 | | 0.238 |  |  | |  |  | |
| Male (n = 233) |  |  |  |  |  |  |  |  |  |  |
| T | | | | | | | | | | |
| T1+T2 (n = 232) | 4.531 | 3.063-6.703 | | ***0.000*** |  |  | |  |  | |
| T3+T4 (n = 124) |  |  |  |  |  |  |  |  |  |  |
| N | | | | | | | | | | |
| N0 (n = 345) | 5.267 | 2.536-10.939 | | ***0.000*** |  | 2.360 | 1.120-4.973 | | | ***0.024*** |
| N1 (n = 11) |  |  |  |  |  |  |  |  |  |  |
| M | | | | | | | | | | |
| M0 (n = 309) | 8.660 | 5.836-12.850 | | ***0.000*** |  | 3.843 | 2.462-5.999 | | | ***0.000*** |
| M1 (n = 47) |  |  |  |  |  |  |  |  |  |  |
| Grade | | | | | | | | | | |
| G1+G2 (n = 172) | 2.940 | 1.922-4.497 | | ***0.000*** |  | 1.843 | 1.187-2.862 | | | ***0.006*** |
| G3+G4 (n = 184) |  |  |  |  |  |  |  |  |  |  |
| Stage | | | | | | | | | | |
| S1+S2 (n = 220) | 6.642 | 4.320-10.212 | | ***0.000*** |  | 3.030 | 1.828-5.022 | | | ***0.000*** |
| S3+S4 (n = 136) |  |  |  |  |  |  |  |  |  |  |

^#^HR estimated from Cox proportional hazard regression model; ^##^95% CI of the estimated HR; ^###^multivariate models were adjusted for gene, age, gender, T, N, M, grade and stage.

Supplementary Table 12. Univariate and multivariate analysis of AC124854.1 mRNA level and patient survival.

| **Variable** | **Univariate analysis** | | | |  | **Multivariate analysis^###^** | | |
| --- | --- | --- | --- | --- | --- | --- | --- | --- |
|  | **HR^#^** | **95% CI^##^** | | **P-value** |  | **HR** | **95% CI** | **P-value** |
| Overall survival (n =445) | | |  | | | | | |
| AC124854.1 | | | | | | | | |
| Low (n = 223) | 0.407 | 0.291-0.568 | | ***0.000*** |  | 0.552 | 0.391-0.779 | ***0.001*** |
| High (n = 222) |  |  |  |  |  |  |  |  |
| Age (years) | | | | | | | | |
| <=60 (n = 219) | 1.727 | 1.253-2.380 | | ***0.001*** |  | 1.659 | 1.201-2.292 | ***0.002*** |
| >60 (n = 226) |  |  |  |  |  |  |  |  |
| Gender | | | | | | | | |
| Female (n = 160) | 0.966 | 0.701-1.331 | | 0.832 |  |  | | |
| Male (n = 285) |  |  |  |  |  |  |  |  |
| T | | | | | | | | |
| T1+T2 (n = 275) | 3.210 | 2.332-4.417 | | ***0.000*** |  |  | | |
| T3+T4 (n = 170) |  |  |  |  |  |  |  |  |
| N | | | | | | | | |
| N0 (n = 431) | 3.362 | 1.768-6.392 | | ***0.000*** |  |  |  |  |
| N1 (n = 14) |  |  |  |  |  |  |  |  |
| M | | | | | | | | |
| M0 (n = 374) | 4.334 | 3.132-5.998 | | ***0.000*** |  | 2.407 | 1.643-3.528 | ***0.000*** |
| M1 (n = 71) |  |  |  |  |  |  |  |  |
| Grade | | | | | | | | |
| G1+G2 (n = 160) | 2.423 | 1.711-3.431 | | ***0.000*** |  | 1.452 | 1.000-2.108 | ***0.050*** |
| G3+G4 (n = 285) |  |  |  |  |  |  |  |  |
| Stage | | | | | | | | |
| S1+S2 (n = 259) | 3.800 | 2.722-5.305 | | ***0.000*** |  | 1.935 | 1.284-2.916 | ***0.002*** |
| S3+S4 (n = 186) |  |  |  |  |  |  |  |  |

^#^HR estimated from Cox proportional hazard regression model; ^##^95% CI of the estimated HR; ^###^multivariate models were adjusted for gene, age, gender, T, N, M, grade and stage.

Supplementary Table 13. Univariate and multivariate analysis of AL355102.4 mRNA level and patient survival.

| **Variable** | **Univariate analysis** | | | |  | **Multivariate analysis^###^** | | | | |
| --- | --- | --- | --- | --- | --- | --- | --- | --- | --- | --- |
|  | **HR^#^** | **95% CI^##^** | | **P-value** |  | **HR** | **95% CI** | | | **P-value** |
| Disease free survival (n =356) | | |  | | | | | | | |
| AL355102.4 | | | | | | | | | | |
| Low (n = 178) | 1.970 | 1.335-2.908 | | ***0.001*** |  | 1.755 | 1.182-2.607 | | | ***0.005*** |
| High (n = 178) |  |  |  |  |  |  |  |  |  |  |
| Age (years) | | | | | | | | | | |
| <=60 (n = 193) | 1.326 | 0.912-1.927 | | 0.140 |  |  |  | | |  |
| >60 (n = 163) |  |  |  |  |  |  |  |  |  |  |
| Gender | | | | | | | | | | |
| Female (n = 123) | 1.276 | 0.851-1.914 | | 0.238 |  |  | |  |  | |
| Male (n = 233) |  |  |  |  |  |  |  |  |  |  |
| T | | | | | | | | | | |
| T1+T2 (n = 232) | 4.531 | 3.063-6.703 | | ***0.000*** |  |  | |  |  | |
| T3+T4 (n = 124) |  |  |  |  |  |  |  |  |  |  |
| N | | | | | | | | | | |
| N0 (n = 345) | 5.267 | 2.536-10.939 | | ***0.000*** |  | 2.325 | 1.103-4.898 | | | ***0.027*** |
| N1 (n = 11) |  |  |  |  |  |  |  |  |  |  |
| M | | | | | | | | | | |
| M0 (n = 309) | 8.660 | 5.836-12.850 | | ***0.000*** |  | 3.886 | 2.488-6.071 | | | ***0.000*** |
| M1 (n = 47) |  |  |  |  |  |  |  |  |  |  |
| Grade | | | | | | | | | | |
| G1+G2 (n = 172) | 2.940 | 1.922-4.497 | | ***0.000*** |  | 1.925 | 1.238-2.992 | | | ***0.004*** |
| G3+G4 (n = 184) |  |  |  |  |  |  |  |  |  |  |
| Stage | | | | | | | | | | |
| S1+S2 (n = 220) | 6.642 | 4.320-10.212 | | ***0.000*** |  | 3.235 | 1.957-5.346 | | | ***0.000*** |
| S3+S4 (n = 136) |  |  |  |  |  |  |  |  |  |  |

^#^HR estimated from Cox proportional hazard regression model; ^##^95% CI of the estimated HR; ^###^multivariate models were adjusted for gene, age, gender, T, N, M, grade and stage.

Supplementary Table 14. Univariate and multivariate analysis of AL355102.4 mRNA level and patient survival.

| **Variable** | **Univariate analysis** | | | |  | **Multivariate analysis^###^** | | |
| --- | --- | --- | --- | --- | --- | --- | --- | --- |
|  | **HR^#^** | **95% CI^##^** | | **P-value** |  | **HR** | **95% CI** | **P-value** |
| Overall survival (n =445) | | |  | | | | | |
| AL355102.4 | | | | | | | | |
| Low (n = 223) | 1.816 | 1.319-2.501 | | ***0.000*** |  | 1.601 | 1.158-2.214 | ***0.004*** |
| High (n = 222) |  |  |  |  |  |  |  |  |
| Age (years) | | | | | | | | |
| <=60 (n = 219) | 1.727 | 1.253-2.380 | | ***0.001*** |  | 1.646 | 1.192-2.271 | ***0.002*** |
| >60 (n = 226) |  |  |  |  |  |  |  |  |
| Gender | | | | | | | | |
| Female (n = 160) | 0.966 | 0.701-1.331 | | 0.832 |  |  | | |
| Male (n = 285) |  |  |  |  |  |  |  |  |
| T | | | | | | | | |
| T1+T2 (n = 275) | 3.210 | 2.332-4.417 | | ***0.000*** |  |  | | |
| T3+T4 (n = 170) |  |  |  |  |  |  |  |  |
| N | | | | | | | | |
| N0 (n = 431) | 3.362 | 1.768-6.392 | | ***0.000*** |  |  |  |  |
| N1 (n = 14) |  |  |  |  |  |  |  |  |
| M | | | | | | | | |
| M0 (n = 374) | 4.334 | 3.132-5.998 | | ***0.000*** |  | 2.220 | 1.513-3.257 | ***0.000*** |
| M1 (n = 71) |  |  |  |  |  |  |  |  |
| Grade | | | | | | | | |
| G1+G2 (n = 160) | 2.423 | 1.711-3.431 | | ***0.000*** |  | 1.558 | 1.075-2.256 | ***0.019*** |
| G3+G4 (n = 285) |  |  |  |  |  |  |  |  |
| Stage | | | | | | | | |
| S1+S2 (n = 259) | 3.800 | 2.722-5.305 | | ***0.000*** |  | 2.185 | 1.456-3.279 | ***0.000*** |
| S3+S4 (n = 186) |  |  |  |  |  |  |  |  |

^#^HR estimated from Cox proportional hazard regression model; ^##^95% CI of the estimated HR; ^###^multivariate models were adjusted for gene, age, gender, T, N, M, grade and stage.

Supplementary Table 15. Univariate and multivariate analysis of AL355075.4 mRNA level and patient survival.

| **Variable** | **Univariate analysis** | | | |  | **Multivariate analysis^###^** | | | | |
| --- | --- | --- | --- | --- | --- | --- | --- | --- | --- | --- |
|  | **HR^#^** | **95% CI^##^** | | **P-value** |  | **HR** | **95% CI** | | | **P-value** |
| Disease free survival (n =356) | | |  | | | | | | | |
| AL355075.4 | | | | | | | | | | |
| Low (n = 178) | 1.589 | 1.085-2.329 | | ***0.017*** |  | 1.600 | 1.082-2.367 | | | ***0.019*** |
| High (n = 178) |  |  |  |  |  |  |  |  |  |  |
| Age (years) | | | | | | | | | | |
| <=60 (n = 193) | 1.326 | 0.912-1.927 | | 0.140 |  |  |  | | |  |
| >60 (n = 163) |  |  |  |  |  |  |  |  |  |  |
| Gender | | | | | | | | | | |
| Female (n = 123) | 1.276 | 0.851-1.914 | | 0.238 |  |  | |  |  | |
| Male (n = 233) |  |  |  |  |  |  |  |  |  |  |
| T | | | | | | | | | | |
| T1+T2 (n = 232) | 4.531 | 3.063-6.703 | | ***0.000*** |  |  | |  |  | |
| T3+T4 (n = 124) |  |  |  |  |  |  |  |  |  |  |
| N | | | | | | | | | | |
| N0 (n = 345) | 5.267 | 2.536-10.939 | | ***0.000*** |  | 2.998 | 1.412-6.367 | | | ***0.004*** |
| N1 (n = 11) |  |  |  |  |  |  |  |  |  |  |
| M | | | | | | | | | | |
| M0 (n = 309) | 8.660 | 5.836-12.850 | | ***0.000*** |  | 4.013 | 2.574-6.259 | | | ***0.000*** |
| M1 (n = 47) |  |  |  |  |  |  |  |  |  |  |
| Grade | | | | | | | | | | |
| G1+G2 (n = 172) | 2.940 | 1.922-4.497 | | ***0.000*** |  | 2.044 | 1.314-3.179 | | | ***0.002*** |
| G3+G4 (n = 184) |  |  |  |  |  |  |  |  |  |  |
| Stage | | | | | | | | | | |
| S1+S2 (n = 220) | 6.642 | 4.320-10.212 | | ***0.000*** |  | 3.150 | 1.909-5.199 | | | ***0.000*** |
| S3+S4 (n = 136) |  |  |  |  |  |  |  |  |  |  |

^#^HR estimated from Cox proportional hazard regression model; ^##^95% CI of the estimated HR; ^###^multivariate models were adjusted for gene, age, gender, T, N, M, grade and stage.

Supplementary Table 16. Univariate and multivariate analysis of AL355075.4 mRNA level and patient survival.

| **Variable** | **Univariate analysis** | | | |  | **Multivariate analysis^###^** | | |
| --- | --- | --- | --- | --- | --- | --- | --- | --- |
|  | **HR^#^** | **95% CI^##^** | | **P-value** |  | **HR** | **95% CI** | **P-value** |
| Overall survival (n =445) | | |  | | | | | |
| AL355075.4 | | | | | | | | |
| Low (n = 223) | 1.775 | 1.288-2.447 | | ***0.000*** |  | 1.781 | 1.289-2.462 | ***0.000*** |
| High (n = 222) |  |  |  |  |  |  |  |  |
| Age (years) | | | | | | | | |
| <=60 (n = 219) | 1.727 | 1.253-2.380 | | ***0.001*** |  | 1.527 | 1.104-2.111 | ***0.010*** |
| >60 (n = 226) |  |  |  |  |  |  |  |  |
| Gender | | | | | | | | |
| Female (n = 160) | 0.966 | 0.701-1.331 | | 0.832 |  |  | | |
| Male (n = 285) |  |  |  |  |  |  |  |  |
| T | | | | | | | | |
| T1+T2 (n = 275) | 3.210 | 2.332-4.417 | | ***0.000*** |  |  | | |
| T3+T4 (n = 170) |  |  |  |  |  |  |  |  |
| N | | | | | | | | |
| N0 (n = 431) | 3.362 | 1.768-6.392 | | ***0.000*** |  |  |  |  |
| N1 (n = 14) |  |  |  |  |  |  |  |  |
| M | | | | | | | | |
| M0 (n = 374) | 4.334 | 3.132-5.998 | | ***0.000*** |  | 2.442 | 1.669-3.574 | ***0.000*** |
| M1 (n = 71) |  |  |  |  |  |  |  |  |
| Grade | | | | | | | | |
| G1+G2 (n = 160) | 2.423 | 1.711-3.431 | | ***0.000*** |  | 1.581 | 1.096-2.279 | ***0.014*** |
| G3+G4 (n = 285) |  |  |  |  |  |  |  |  |
| Stage | | | | | | | | |
| S1+S2 (n = 259) | 3.800 | 2.722-5.305 | | ***0.000*** |  | 2.201 | 1.470-3.296 | ***0.000*** |
| S3+S4 (n = 186) |  |  |  |  |  |  |  |  |

^#^HR estimated from Cox proportional hazard regression model; ^##^95% CI of the estimated HR; ^###^multivariate models were adjusted for gene, age, gender, T, N, M, grade and stage.

Supplementary Table 17. Univariate and multivariate analysis of TMEM246-AS1 mRNA level and patient survival.

| **Variable** | **Univariate analysis** | | | |  | **Multivariate analysis^###^** | | | | |
| --- | --- | --- | --- | --- | --- | --- | --- | --- | --- | --- |
|  | **HR^#^** | **95% CI^##^** | | **P-value** |  | **HR** | **95% CI** | | | **P-value** |
| Disease free survival (n =356) | | |  | | | | | | | |
| TMEM246-AS1 | | | | | | | | | | |
| Low (n = 178) | 0.477 | 0.321-0.709 | | ***0.000*** |  | 0.586 | 0.389-0.883 | | | ***0.011*** |
| High (n = 178) |  |  |  |  |  |  |  |  |  |  |
| Age (years) | | | | | | | | | | |
| <=60 (n = 193) | 1.326 | 0.912-1.927 | | 0.140 |  |  |  | | |  |
| >60 (n = 163) |  |  |  |  |  |  |  |  |  |  |
| Gender | | | | | | | | | | |
| Female (n = 123) | 1.276 | 0.851-1.914 | | 0.238 |  |  | |  |  | |
| Male (n = 233) |  |  |  |  |  |  |  |  |  |  |
| T | | | | | | | | | | |
| T1+T2 (n = 232) | 4.531 | 3.063-6.703 | | ***0.000*** |  |  | |  |  | |
| T3+T4 (n = 124) |  |  |  |  |  |  |  |  |  |  |
| N | | | | | | | | | | |
| N0 (n = 345) | 5.267 | 2.536-10.939 | | ***0.000*** |  | 2.330 | 1.104-4.916 | | | ***0.026*** |
| N1 (n = 11) |  |  |  |  |  |  |  |  |  |  |
| M | | | | | | | | | | |
| M0 (n = 309) | 8.660 | 5.836-12.850 | | ***0.000*** |  | 3.628 | 2.317-5.680 | | | ***0.000*** |
| M1 (n = 47) |  |  |  |  |  |  |  |  |  |  |
| Grade | | | | | | | | | | |
| G1+G2 (n = 172) | 2.940 | 1.922-4.497 | | ***0.000*** |  | 1.842 | 1.185-2.862 | | | ***0.007*** |
| G3+G4 (n = 184) |  |  |  |  |  |  |  |  |  |  |
| Stage | | | | | | | | | | |
| S1+S2 (n = 220) | 6.642 | 4.320-10.212 | | ***0.000*** |  | 3.352 | 2.028-5.541 | | | ***0.000*** |
| S3+S4 (n = 136) |  |  |  |  |  |  |  |  |  |  |

^#^HR estimated from Cox proportional hazard regression model; ^##^95% CI of the estimated HR; ^###^multivariate models were adjusted for gene, age, gender, T, N, M, grade and stage.

Supplementary Table 18. Univariate and multivariate analysis of TMEM246-AS1 mRNA level and patient survival

| **Variable** | **Univariate analysis** | | | |  | **Multivariate analysis^###^** | | |
| --- | --- | --- | --- | --- | --- | --- | --- | --- |
|  | **HR^#^** | **95% CI^##^** | | **P-value** |  | **HR** | **95% CI** | **P-value** |
| Overall survival (n =445) | | |  | | | | | |
| TMEM246-AS1 | | | | | | | | |
| Low (n = 223) | 0.543 | 0.394-0.750 | | ***0.000*** |  | 0.624 | 0.449-0.867 | ***0.005*** |
| High (n = 222) |  |  |  |  |  |  |  |  |
| Age (years) | | | | | | | | |
| <=60 (n = 219) | 1.727 | 1.253-2.380 | | ***0.001*** |  | 1.678 | 1.215-2.318 | ***0.002*** |
| >60 (n = 226) |  |  |  |  |  |  |  |  |
| Gender | | | | | | | | |
| Female (n = 160) | 0.966 | 0.701-1.331 | | 0.832 |  |  | | |
| Male (n = 285) |  |  |  |  |  |  |  |  |
| T | | | | | | | | |
| T1+T2 (n = 275) | 3.210 | 2.332-4.417 | | ***0.000*** |  |  | | |
| T3+T4 (n = 170) |  |  |  |  |  |  |  |  |
| N | | | | | | | | |
| N0 (n = 431) | 3.362 | 1.768-6.392 | | ***0.000*** |  | 2.058 | 1.068-3.965 | ***0.031*** |
| N1 (n = 14) |  |  |  |  |  |  |  |  |
| M | | | | | | | | |
| M0 (n = 374) | 4.334 | 3.132-5.998 | | ***0.000*** |  | 2.150 | 1.461-3.166 | ***0.000*** |
| M1 (n = 71) |  |  |  |  |  |  |  |  |
| Grade | | | | | | | | |
| G1+G2 (n = 160) | 2.423 | 1.711-3.431 | | ***0.000*** |  | 1.527 | 1.055-2.211 | ***0.025*** |
| G3+G4 (n = 285) |  |  |  |  |  |  |  |  |
| Stage | | | | | | | | |
| S1+S2 (n = 259) | 3.800 | 2.722-5.305 | | ***0.000*** |  | 2.141 | 1.422-3.224 | ***0.000*** |
| S3+S4 (n = 186) |  |  |  |  |  |  |  |  |

^#^HR estimated from Cox proportional hazard regression model; ^##^95% CI of the estimated HR; ^###^multivariate models were adjusted for gene, age, gender, T, N, M, grade and stage.

Supplementary Table 19. Univariate and multivariate analysis of AL133467.3 mRNA level and patient survival.

| **Variable** | **Univariate analysis** | | | |  | **Multivariate analysis^###^** | | | | |
| --- | --- | --- | --- | --- | --- | --- | --- | --- | --- | --- |
|  | **HR^#^** | **95% CI^##^** | | **P-value** |  | **HR** | **95% CI** | | | **P-value** |
| Disease free survival (n =356) | | |  | | | | | | | |
| AL133467.3 | | | | | | | | | | |
| Low (n = 178) | 0.391 | 0.261-0.584 | | ***0.000*** |  | 0.637 | 0.419-0.966 | | | ***0.034*** |
| High (n = 178) |  |  |  |  |  |  |  |  |  |  |
| Age (years) | | | | | | | | | | |
| <=60 (n = 193) | 1.326 | 0.912-1.927 | | 0.140 |  |  |  | | |  |
| >60 (n = 163) |  |  |  |  |  |  |  |  |  |  |
| Gender | | | | | | | | | | |
| Female (n = 123) | 1.276 | 0.851-1.914 | | 0.238 |  |  | |  |  | |
| Male (n = 233) |  |  |  |  |  |  |  |  |  |  |
| T | | | | | | | | | | |
| T1+T2 (n = 232) | 4.531 | 3.063-6.703 | | ***0.000*** |  |  | |  |  | |
| T3+T4 (n = 124) |  |  |  |  |  |  |  |  |  |  |
| N | | | | | | | | | | |
| N0 (n = 345) | 5.267 | 2.536-10.939 | | ***0.000*** |  | 2.299 | 1.087-4.860 | | | ***0.029*** |
| N1 (n = 11) |  |  |  |  |  |  |  |  |  |  |
| M | | | | | | | | | | |
| M0 (n = 309) | 8.660 | 5.836-12.850 | | ***0.000*** |  | 4.070 | 2.605-6.361 | | | ***0.000*** |
| M1 (n = 47) |  |  |  |  |  |  |  |  |  |  |
| Grade | | | | | | | | | | |
| G1+G2 (n = 172) | 2.940 | 1.922-4.497 | | ***0.000*** |  | 1.854 | 1.196-2.874 | | | ***0.006*** |
| G3+G4 (n = 184) |  |  |  |  |  |  |  |  |  |  |
| Stage | | | | | | | | | | |
| S1+S2 (n = 220) | 6.642 | 4.320-10.212 | | ***0.000*** |  | 2.973 | 1.788-4.942 | | | ***0.000*** |
| S3+S4 (n = 136) |  |  |  |  |  |  |  |  |  |  |

^#^HR estimated from Cox proportional hazard regression model; ^##^95% CI of the estimated HR; ^###^multivariate models were adjusted for gene, age, gender, T, N, M, grade and stage.

Supplementary Table 20. Univariate and multivariate analysis of AL133467.3 mRNA level and patient survival.

| **Variable** | **Univariate analysis** | | | |  | **Multivariate analysis^###^** | | |
| --- | --- | --- | --- | --- | --- | --- | --- | --- |
|  | **HR^#^** | **95% CI^##^** | | **P-value** |  | **HR** | **95% CI** | **P-value** |
| Overall survival (n =445) | | |  | | | | | |
| AL133467.3 | | | | | | | | |
| Low (n = 223) | 0.478 | 0.345-0.662 | | ***0.000*** |  | 0.665 | 0.475-0.930 | ***0.017*** |
| High (n = 222) |  |  |  |  |  |  |  |  |
| Age (years) | | | | | | | | |
| <=60 (n = 219) | 1.727 | 1.253-2.380 | | ***0.001*** |  | 1.598 | 1.158-2.205 | ***0.004*** |
| >60 (n = 226) |  |  |  |  |  |  |  |  |
| Gender | | | | | | | | |
| Female (n = 160) | 0.966 | 0.701-1.331 | | 0.832 |  |  | | |
| Male (n = 285) |  |  |  |  |  |  |  |  |
| T | | | | | | | | |
| T1+T2 (n = 275) | 3.177 | 2.311-4.368 | | ***0.000*** |  |  | | |
| T3+T4 (n = 170) |  |  |  |  |  |  |  |  |
| N | | | | | | | | |
| N0 (n = 431) | 3.362 | 1.768-6.392 | | ***0.000*** |  |  |  |  |
| N1 (n = 14) |  |  |  |  |  |  |  |  |
| M | | | | | | | | |
| M0 (n = 374) | 4.334 | 3.132-5.998 | | ***0.000*** |  | 2.353 | 1.608-3.444 | ***0.000*** |
| M1 (n = 71) |  |  |  |  |  |  |  |  |
| Grade | | | | | | | | |
| G1+G2 (n = 160) | 2.423 | 1.711-3.431 | | ***0.000*** |  | 1.467 | 1.013-2.124 | ***0.043*** |
| G3+G4 (n = 285) |  |  |  |  |  |  |  |  |
| Stage | | | | | | | | |
| S1+S2 (n = 259) | 3.800 | 2.722-5.305 | | ***0.000*** |  | 2.081 | 1.386-3.125 | ***0.000*** |
| S3+S4 (n = 186) |  |  |  |  |  |  |  |  |

^#^HR estimated from Cox proportional hazard regression model; ^##^95% CI of the estimated HR; ^###^multivariate models were adjusted for gene, age, gender, T, N, M, grade and stage.

Supplementary Table 21. Univariate and multivariate analysis of ZNF582-AS1 mRNA level and patient survival.

| **Variable** | **Univariate analysis** | | | |  | **Multivariate analysis^###^** | | | | |
| --- | --- | --- | --- | --- | --- | --- | --- | --- | --- | --- |
|  | **HR^#^** | **95% CI^##^** | | **P-value** |  | **HR** | **95% CI** | | | **P-value** |
| Disease free survival (n =356) | | |  | | | | | | | |
| ZNF582-AS1 | | | | | | | | | | |
| Low (n = 178) | 0.507 | 0.344-0.747 | | ***0.001*** |  | 0.670 | 0.449-1.000 | | | ***0.050*** |
| High (n = 178) |  |  |  |  |  |  |  |  |  |  |
| Age (years) | | | | | | | | | | |
| <=60 (n = 193) | 1.326 | 0.912-1.927 | | 0.140 |  |  |  | | |  |
| >60 (n = 163) |  |  |  |  |  |  |  |  |  |  |
| Gender | | | | | | | | | | |
| Female (n = 123) | 1.276 | 0.851-1.914 | | 0.238 |  |  | |  |  | |
| Male (n = 233) |  |  |  |  |  |  |  |  |  |  |
| T | | | | | | | | | | |
| T1+T2 (n = 232) | 4.531 | 3.063-6.703 | | ***0.000*** |  |  | |  |  | |
| T3+T4 (n = 124) |  |  |  |  |  |  |  |  |  |  |
| N | | | | | | | | | | |
| N0 (n = 345) | 5.267 | 2.536-10.939 | | ***0.000*** |  | 2.340 | 1.107-4.945 | | | ***0.026*** |
| N1 (n = 11) |  |  |  |  |  |  |  |  |  |  |
| M | | | | | | | | | | |
| M0 (n = 309) | 8.660 | 5.836-12.850 | | ***0.000*** |  | 3.670 | 2.336-5.766 | | | ***0.000*** |
| M1 (n = 47) |  |  |  |  |  |  |  |  |  |  |
| Grade | | | | | | | | | | |
| G1+G2 (n = 172) | 2.940 | 1.922-4.497 | | ***0.000*** |  | 1.939 | 1.253-3.001 | | | ***0.003*** |
| G3+G4 (n = 184) |  |  |  |  |  |  |  |  |  |  |
| Stage | | | | | | | | | | |
| S1+S2 (n = 220) | 6.642 | 4.320-10.212 | | ***0.000*** |  | 3.342 | 2.030-5.502 | | | ***0.000*** |
| S3+S4 (n = 136) |  |  |  |  |  |  |  |  |  |  |

^#^HR estimated from Cox proportional hazard regression model; ^##^95% CI of the estimated HR; ^###^multivariate models were adjusted for gene, age, gender, T, N, M, grade and stage.

Supplementary Table 22. Univariate and multivariate analysis of ZNF582-AS1 mRNA level and patient survival.

| **Variable** | **Univariate analysis** | | | |  | **Multivariate analysis^###^** | | |
| --- | --- | --- | --- | --- | --- | --- | --- | --- |
|  | **HR^#^** | **95% CI^##^** | | **P-value** |  | **HR** | **95% CI** | **P-value** |
| Overall survival (n =445) | | |  | | | | | |
| ZNF582-AS1 | | | | | | | | |
| Low (n = 223) | 0.540 | 0.392-0.744 | | ***0.000*** |  | 0.695 | 0.502-0.963 | ***0.029*** |
| High (n = 222) |  |  |  |  |  |  |  |  |
| Age (years) | | | | | | | | |
| <=60 (n = 219) | 1.727 | 1.253-2.380 | | ***0.001*** |  | 1.620 | 1.173-2.236 | ***0.003*** |
| >60 (n = 226) |  |  |  |  |  |  |  |  |
| Gender | | | | | | | | |
| Female (n = 160) | 0.966 | 0.701-1.331 | | 0.832 |  |  | | |
| Male (n = 285) |  |  |  |  |  |  |  |  |
| T | | | | | | | | |
| T1+T2 (n = 275) | 3.210 | 2.332-4.417 | | ***0.000*** |  |  | | |
| T3+T4 (n = 170) |  |  |  |  |  |  |  |  |
| N | | | | | | | | |
| N0 (n = 431) | 3.362 | 1.768-6.392 | | ***0.000*** |  |  |  |  |
| N1 (n = 14) |  |  |  |  |  |  |  |  |
| M | | | | | | | | |
| M0 (n = 374) | 4.334 | 3.132-5.998 | | ***0.000*** |  | 2.261 | 1.539-3.322 | ***0.000*** |
| M1 (n = 71) |  |  |  |  |  |  |  |  |
| Grade | | | | | | | | |
| G1+G2 (n = 160) | 2.423 | 1.711-3.431 | | ***0.000*** |  | 1.528 | 1.059-2.204 | ***0.024*** |
| G3+G4 (n = 285) |  |  |  |  |  |  |  |  |
| Stage | | | | | | | | |
| S1+S2 (n = 259) | 3.800 | 2.722-5.305 | | ***0.000*** |  | 2.192 | 1.467-3.274 | ***0.000*** |
| S3+S4 (n = 186) |  |  |  |  |  |  |  |  |

^#^HR estimated from Cox proportional hazard regression model; ^##^95% CI of the estimated HR; ^###^multivariate models were adjusted for gene, age, gender, T, N, M, grade and stage.

Supplementary Table 23. Univariate and multivariate analysis of LINC01510 mRNA level and patient survival.

| **Variable** | **Univariate analysis** | | | |  | **Multivariate analysis^###^** | | | | |
| --- | --- | --- | --- | --- | --- | --- | --- | --- | --- | --- |
|  | **HR^#^** | **95% CI^##^** | | **P-value** |  | **HR** | **95% CI** | | | **P-value** |
| Disease free survival (n =356) | | |  | | | | | | | |
| LINC01510 | | | | | | | | | | |
| Low (n = 178) | 0.439 | 0.296-0.652 | | ***0.000*** |  | 0.597 | 0.399-0.896 | | | ***0.013*** |
| High (n = 178) |  |  |  |  |  |  |  |  |  |  |
| Age (years) | | | | | | | | | | |
| <=60 (n = 193) | 1.326 | 0.912-1.927 | | 0.140 |  |  |  | | |  |
| >60 (n = 163) |  |  |  |  |  |  |  |  |  |  |
| Gender | | | | | | | | | | |
| Female (n = 123) | 1.276 | 0.851-1.914 | | 0.238 |  |  | |  |  | |
| Male (n = 233) |  |  |  |  |  |  |  |  |  |  |
| T | | | | | | | | | | |
| T1+T2 (n = 232) | 4.531 | 3.063-6.703 | | ***0.000*** |  |  | |  |  | |
| T3+T4 (n = 124) |  |  |  |  |  |  |  |  |  |  |
| N | | | | | | | | | | |
| N0 (n = 345) | 5.267 | 2.536-10.939 | | ***0.000*** |  | 2.391 | 1.136-5.035 | | | ***0.022*** |
| N1 (n = 11) |  |  |  |  |  |  |  |  |  |  |
| M | | | | | | | | | | |
| M0 (n = 309) | 8.660 | 5.836-12.850 | | ***0.000*** |  | 4.136 | 2.650-6.453 | | | ***0.000*** |
| M1 (n = 47) |  |  |  |  |  |  |  |  |  |  |
| Grade | | | | | | | | | | |
| G1+G2 (n = 172) | 2.940 | 1.922-4.497 | | ***0.000*** |  | 1.835 | 1.181-2.851 | | | ***0.007*** |
| G3+G4 (n = 184) |  |  |  |  |  |  |  |  |  |  |
| Stage | | | | | | | | | | |
| S1+S2 (n = 220) | 6.642 | 4.320-10.212 | | ***0.000*** |  | 2.960 | 1.782-4.917 | | | ***0.000*** |
| S3+S4 (n = 136) |  |  |  |  |  |  |  |  |  |  |

^#^HR estimated from Cox proportional hazard regression model; ^##^95% CI of the estimated HR; ^###^multivariate models were adjusted for gene, age, gender, T, N, M, grade and stage.

Supplementary Table 24. Univariate and multivariate analysis of LINC01510 mRNA level and patient survival.

| **Variable** | **Univariate analysis** | | | |  | **Multivariate analysis^###^** | | |
| --- | --- | --- | --- | --- | --- | --- | --- | --- |
|  | **HR^#^** | **95% CI^##^** | | **P-value** |  | **HR** | **95% CI** | **P-value** |
| Overall survival (n =445) | | |  | | | | | |
| LINC01510 | | | | | | | | |
| Low (n = 223) | 0.470 | 0.340-0.651 | | ***0.000*** |  | 0.665 | 0.475-0.930 | ***0.017*** |
| High (n = 222) |  |  |  |  |  |  |  |  |
| Age (years) | | | | | | | | |
| <=60 (n = 219) | 1.727 | 1.253-2.380 | | ***0.001*** |  | 1.621 | 1.175-2.237 | ***0.003*** |
| >60 (n = 226) |  |  |  |  |  |  |  |  |
| Gender | | | | | | | | |
| Female (n = 160) | 0.966 | 0.701-1.331 | | 0.832 |  |  | | |
| Male (n = 285) |  |  |  |  |  |  |  |  |
| T | | | | | | | | |
| T1+T2 (n = 275) | 3.210 | 2.332-4.417 | | ***0.000*** |  |  | | |
| T3+T4 (n = 170) |  |  |  |  |  |  |  |  |
| N | | | | | | | | |
| N0 (n = 431) | 3.362 | 1.768-6.392 | | ***0.000*** |  |  |  |  |
| N1 (n = 14) |  |  |  |  |  |  |  |  |
| M | | | | | | | | |
| M0 (n = 374) | 4.334 | 3.132-5.998 | | ***0.000*** |  | 2.287 | 1.560-3.351 | ***0.000*** |
| M1 (n = 71) |  |  |  |  |  |  |  |  |
| Grade | | | | | | | | |
| G1+G2 (n = 160) | 2.423 | 1.711-3.431 | | ***0.000*** |  | 1.511 | 1.044-2.188 | ***0.029*** |
| G3+G4 (n = 285) |  |  |  |  |  |  |  |  |
| Stage | | | | | | | | |
| S1+S2 (n = 259) | 3.800 | 2.722-5.305 | | ***0.000*** |  | 2.050 | 1.362-3.084 | ***0.001*** |
| S3+S4 (n = 186) |  |  |  |  |  |  |  |  |

^#^HR estimated from Cox proportional hazard regression model; ^##^95% CI of the estimated HR; ^###^multivariate models were adjusted for gene, age, gender, T, N, M, grade and stage.

Supplementary Table 25. Univariate and multivariate analysis of PSMG3-AS1 mRNA level and patient survival.

| **Variable** | **Univariate analysis** | | | |  | **Multivariate analysis^###^** | | | | |
| --- | --- | --- | --- | --- | --- | --- | --- | --- | --- | --- |
|  | **HR^#^** | **95% CI^##^** | | **P-value** |  | **HR** | **95% CI** | | | **P-value** |
| Disease free survival (n =356) | | |  | | | | | | | |
| PSMG3-AS1 | | | | | | | | | | |
| Low (n = 178) | 0.539 | 0.366-0.794 | | ***0.002*** |  | 0.613 | 0.414-0.909 | | | ***0.015*** |
| High (n = 178) |  |  |  |  |  |  |  |  |  |  |
| Age (years) | | | | | | | | | | |
| <=60 (n = 193) | 1.326 | 0.912-1.927 | | 0.140 |  |  |  | | |  |
| >60 (n = 163) |  |  |  |  |  |  |  |  |  |  |
| Gender | | | | | | | | | | |
| Female (n = 123) | 1.276 | 0.851-1.914 | | 0.238 |  |  | |  |  | |
| Male (n = 233) |  |  |  |  |  |  |  |  |  |  |
| T | | | | | | | | | | |
| T1+T2 (n = 232) | 4.531 | 3.063-6.703 | | ***0.000*** |  |  | |  |  | |
| T3+T4 (n = 124) |  |  |  |  |  |  |  |  |  |  |
| N | | | | | | | | | | |
| N0 (n = 345) | 5.267 | 2.536-10.939 | | ***0.000*** |  | 2.779 | 1.320-5.848 | | | ***0.007*** |
| N1 (n = 11) |  |  |  |  |  |  |  |  |  |  |
| M | | | | | | | | | | |
| M0 (n = 309) | 8.660 | 5.836-12.850 | | ***0.000*** |  | 4.250 | 2.713-6.659 | | | ***0.000*** |
| M1 (n = 47) |  |  |  |  |  |  |  |  |  |  |
| Grade | | | | | | | | | | |
| G1+G2 (n = 172) | 2.940 | 1.922-4.497 | | ***0.000*** |  | 1.849 | 1.195-2.862 | | | ***0.006*** |
| G3+G4 (n = 184) |  |  |  |  |  |  |  |  |  |  |
| Stage | | | | | | | | | | |
| S1+S2 (n = 220) | 6.642 | 4.320-10.212 | | ***0.000*** |  | 3.131 | 1.898-5.164 | | | ***0.000*** |
| S3+S4 (n = 136) |  |  |  |  |  |  |  |  |  |  |

^#^HR estimated from Cox proportional hazard regression model; ^##^95% CI of the estimated HR; ^###^multivariate models were adjusted for gene, age, gender, T, N, M, grade and stage.

Supplementary Table 26. Univariate and multivariate analysis of PSMG3-AS1 mRNA level and patient survival.

| **Variable** | **Univariate analysis** | | | |  | **Multivariate analysis^###^** | | |
| --- | --- | --- | --- | --- | --- | --- | --- | --- |
|  | **HR^#^** | **95% CI^##^** | | **P-value** |  | **HR** | **95% CI** | **P-value** |
| Overall survival (n =445) | | |  | | | | | |
| PSMG3-AS1 | | | | | | | | |
| Low (n = 223) | 0.571 | 0.415-0.786 | | ***0.001*** |  | 0.665 | 0.482-0.918 | ***0.013*** |
| High (n = 222) |  |  |  |  |  |  |  |  |
| Age (years) | | | | | | | | |
| <=60 (n = 219) | 1.727 | 1.253-2.380 | | ***0.001*** |  | 1.679 | 1.215-2.320 | ***0.002*** |
| >60 (n = 226) |  |  |  |  |  |  |  |  |
| Gender | | | | | | | | |
| Female (n = 160) | 0.966 | 0.701-1.331 | | 0.832 |  |  | | |
| Male (n = 285) |  |  |  |  |  |  |  |  |
| T | | | | | | | | |
| T1+T2 (n = 275) | 3.210 | 2.332-4.417 | | ***0.000*** |  |  | | |
| T3+T4 (n = 170) |  |  |  |  |  |  |  |  |
| N | | | | | | | | |
| N0 (n = 431) | 3.362 | 1.768-6.392 | | ***0.000*** |  |  |  |  |
| N1 (n = 14) |  |  |  |  |  |  |  |  |
| M | | | | | | | | |
| M0 (n = 374) | 4.334 | 3.132-5.998 | | ***0.000*** |  | 2.369 | 1.616-3.472 | ***0.000*** |
| M1 (n = 71) |  |  |  |  |  |  |  |  |
| Grade | | | | | | | | |
| G1+G2 (n = 160) | 2.423 | 1.711-3.431 | | ***0.000*** |  | 1.508 | 1.044-2.179 | ***0.029*** |
| G3+G4 (n = 285) |  |  |  |  |  |  |  |  |
| Stage | | | | | | | | |
| S1+S2 (n = 259) | 3.800 | 2.722-5.305 | | ***0.000*** |  | 2.130 | 1.425-3.185 | ***0.000*** |
| S3+S4 (n = 186) |  |  |  |  |  |  |  |  |

^#^HR estimated from Cox proportional hazard regression model; ^##^95% CI of the estimated HR; ^###^multivariate models were adjusted for gene, age, gender, T, N, M, grade and stage.

Supplementary Table 27. The characteristics of IGFL2-AS1 in renal cell carcinoma.

| **Characteristic** | | **lncRNA IGFL2-AS1** | | **P-value** |
| --- | --- | --- | --- | --- |
|  |  | Low(n=223) | High(n=222) |  |
| Age (years) | <=60 | 108 | 111 | 0.7405 |
|  | >60 | 115 | 111 |  |
| Gender | Female | 90 | 70 | 0.0524 |
|  | Male | 133 | 152 |  |
| T | T1+T2 | 154 | 121 | ***0.0016*** |
|  | T3+T4 | 69 | 101 |  |
| N | N0 | 219 | 212 | 0.1014 |
|  | N1 | 4 | 10 |  |
| M | M0 | 194 | 180 | 0.0885 |
|  | M1 | 29 | 42 |  |
| Grade | G1+G2 | 108 | 95 | 0.2325 |
|  | G3+G4 | 115 | 127 |  |
| Stage | S1+S2 | 145 | 114 | ***0.0035*** |
|  | S3+S4 | 78 | 108 |  |

Supplementary Table 28. The characteristics of AC023043.1 in renal cell carcinoma.

| **Characteristic** | | **lncRNA-AC023043.1** | | **P-value** |
| --- | --- | --- | --- | --- |
|  |  | **Low(n=223)** | **High(n=222)** |  |
| Age (years) | <=60 | 110 | 109 | 0.9616 |
|  | >60 | 113 | 113 |  |
| Gender | Female | 91 | 69 | ***0.0325*** |
|  | Male | 132 | 153 |  |
| T | T1+T2 | 169 | 106 | ***<0.0001*** |
|  | T3+T4 | 54 | 116 |  |
| N | N0 | 222 | 209 | ***0.0011*** |
|  | N1 | 1 | 13 |  |
| M | M0 | 208 | 166 | ***<0.0001*** |
|  | M1 | 15 | 56 |  |
| Grade | G1+G2 | 127 | 76 | ***<0.0001*** |
|  | G3+G4 | 96 | 146 |  |
| Stage | S1+S2 | 163 | 96 | ***<0.0001*** |
|  | S3+S4 | 60 | 126 |  |

Supplementary Table 29. The characteristics of AP000439.2 in renal cell carcinoma.

| **Characteristic** | | **lncRNA-AP000439.2** | | **P-value** |
| --- | --- | --- | --- | --- |
|  |  | **Low(n=223)** | **High(n=222)** |  |
| Age (years) | <=60 | 108 | 111 | 0.7405 |
|  | >60 | 115 | 111 |  |
| Gender | Female | 66 | 94 | ***0.0051*** |
|  | Male | 157 | 128 |  |
| T | T1+T2 | 114 | 161 | ***<0.0001*** |
|  | T3+T4 | 109 | 61 |  |
| N | N0 | 213 | 218 | 0.1050 |
|  | N1 | 10 | 4 |  |
| M | M0 | 174 | 200 | ***0.0005*** |
|  | M1 | 49 | 22 |  |
| Grade | G1+G2 | 82 | 121 | ***0.0002*** |
|  | G3+G4 | 141 | 101 |  |
| Stage | S1+S2 | 108 | 151 | ***<0.0001*** |
|  | S3+S4 | 115 | 71 |  |

Supplementary Table 30. The characteristics of AC124854.1 in renal cell carcinoma.

| **Characteristic** | | **lncRNA-AC124854.1** | | **P-value** |
| --- | --- | --- | --- | --- |
|  |  | **Low(n=223)** | **High(n=222)** |  |
| Age (years) | <=60 | 103 | 116 | 0.2008 |
|  | >60 | 120 | 106 |  |
| Gender | Female | 79 | 81 | 0.8157 |
|  | Male | 144 | 141 |  |
| T | T1+T2 | 112 | 163 | ***<0.0001*** |
|  | T3+T4 | 111 | 59 |  |
| N | N0 | 211 | 220 | ***0.0068*** |
|  | N1 | 12 | 2 |  |
| M | M0 | 175 | 199 | ***0.0013*** |
|  | M1 | 48 | 23 |  |
| Grade | G1+G2 | 82 | 121 | ***0.0002*** |
|  | G3+G4 | 141 | 101 |  |
| Stage | S1+S2 | 102 | 157 | ***<0.0001*** |
|  | S3+S4 | 121 | 65 |  |

Supplementary Table 31. The characteristics of AL355102.4 in renal cell carcinoma.

| **Characteristic** | | **lncRNA-AL355102.4** | | **P-value** |
| --- | --- | --- | --- | --- |
|  |  | **Low(n=223)** | **High(n=222)** |  |
| Age (years) | <=60 | 107 | 112 | 0.6025 |
|  | >60 | 116 | 110 |  |
| Gender | Female | 93 | 67 | ***0.0113*** |
|  | Male | 130 | 155 |  |
| T | T1+T2 | 148 | 127 | ***0.0468*** |
|  | T3+T4 | 75 | 95 |  |
| N | N0 | 218 | 213 | 0.2736 |
|  | N1 | 5 | 9 |  |
| M | M0 | 197 | 177 | ***0.0131*** |
|  | M1 | 26 | 45 |  |
| Grade | G1+G2 | 103 | 100 | 0.8087 |
|  | G3+G4 | 120 | 122 |  |
| Stage | S1+S2 | 138 | 121 | 0.1146 |
|  | S3+S4 | 85 | 101 |  |

**Supplementary Table 32. The characteristics of AL355075.4 in renal cell carcinoma.**

| **Characteristic** | | **lncRNA-AL355075.4** | | **P-value** |
| --- | --- | --- | --- | --- |
|  |  | **Low(n=223)** | **High(n=222)** |  |
| Age (years) | <=60 | 115 | 104 | 0.3191 |
|  | >60 | 108 | 118 |  |
| Gender | Female | 87 | 73 | 0.1778 |
|  | Male | 136 | 149 |  |
| T | T1+T2 | 148 | 127 | ***0.0468*** |
|  | T3+T4 | 75 | 95 |  |
| N | N0 | 216 | 215 | 0.9932 |
|  | N1 | 7 | 7 |  |
| M | M0 | 187 | 187 | 0.9134 |
|  | M1 | 36 | 35 |  |
| Grade | G1+G2 | 113 | 90 | ***0.0319*** |
|  | G3+G4 | 110 | 132 |  |
| Stage | S1+S2 | 140 | 119 | ***0.0497*** |
|  | S3+S4 | 83 | 103 |  |

Supplementary Table 33. The characteristics of TMEM246-AS1 in renal cell carcinoma.

| **Characteristic** | | **lncRNA TMEM246-AS1** | | **P-value** |
| --- | --- | --- | --- | --- |
|  |  | **Low(n=223)** | **High(n=222)** |  |
| Age (years) | <=60 | 111 | 108 | 0.8120 |
|  | >60 | 112 | 114 |  |
| Gender | Female | 62 | 98 | ***0.0003*** |
|  | Male | 161 | 124 |  |
| T | T1+T2 | 125 | 150 | ***0.0124*** |
|  | T3+T4 | 98 | 72 |  |
| N | N0 | 213 | 218 | 0.1050 |
|  | N1 | 10 | 4 |  |
| M | M0 | 177 | 197 | ***0.0070*** |
|  | M1 | 46 | 25 |  |
| Grade | G1+G2 | 92 | 111 | 0.0641 |
|  | G3+G4 | 131 | 111 |  |
| Stage | S1+S2 | 116 | 143 | ***0.0080*** |
|  | S3+S4 | 107 | 79 |  |

**Supplementary Table 34. The characteristics of AL133467.3 in renal cell carcinoma.**

| **Characteristic** | | **lncRNA-AL133467.3** | | **P-value** |
| --- | --- | --- | --- | --- |
|  |  | **Low(n=223)** | **High(n=222)** |  |
| Age (years) | <=60 | 102 | 117 | 0.1418 |
|  | >60 | 121 | 105 |  |
| Gender | Female | 63 | 97 | ***0.0007*** |
|  | Male | 160 | 125 |  |
| T | T1+T2 | 115 | 163 | ***<0.0001*** |
|  | T3+T4 | 108 | 59 |  |
| N | N0 | 210 | 221 | ***0.0012*** |
|  | N1 | 13 | 1 |  |
| M | M0 | 175 | 199 | ***0.0013*** |
|  | M1 | 48 | 23 |  |
| Grade | G1+G2 | 80 | 123 | ***<0.0001*** |
|  | G3+G4 | 143 | 99 |  |
| Stage | S1+S2 | 103 | 156 | ***<0.0001*** |
|  | S3+S4 | 120 | 66 |  |

Supplementary Table 35. The characteristics of ZNF582-AS1 in renal cell carcinoma.

| **Characteristic** | | **lncRNA ZNF582-AS1** | | **P-value** |
| --- | --- | --- | --- | --- |
|  |  | **Low(n=223)** | **High(n=222)** |  |
| Age (years) | <=60 | 97 | 122 | ***0.0156*** |
|  | >60 | 126 | 100 |  |
| Gender | Female | 73 | 87 | 0.1560 |
|  | Male | 150 | 135 |  |
| T | T1+T2 | 123 | 152 | ***0.0039*** |
|  | T3+T4 | 100 | 70 |  |
| N | N0 | 212 | 219 | ***0.0305*** |
|  | N1 | 11 | 3 |  |
| M | M0 | 178 | 196 | ***0.0147*** |
|  | M1 | 45 | 26 |  |
| Grade | G1+G2 | 88 | 115 | ***0.0090*** |
|  | G3+G4 | 135 | 107 |  |
| Stage | S1+S2 | 114 | 145 | ***0.0024*** |
|  | S3+S4 | 109 | 77 |  |

Supplementary Table 36. The characteristics of LINC01510 in renal cell crcinoma

| **Characteristic** | | **lncRNA-LINC01510** | | **P-value** |
| --- | --- | --- | --- | --- |
|  |  | **Low(n=223)** | **High(n=222)** |  |
| Age (years) | <=60 | 103 | 116 | 0.2008 |
|  | >60 | 120 | 106 |  |
| Gender | Female | 71 | 89 | 0.0697 |
|  | Male | 152 | 133 |  |
| T | T1+T2 | 114 | 161 | ***<0.0001*** |
|  | T3+T4 | 109 | 61 |  |
| N | N0 | 213 | 218 | 0.1050 |
|  | N1 | 10 | 4 |  |
| M | M0 | 178 | 196 | ***0.0147*** |
|  | M1 | 45 | 26 |  |
| Grade | G1+G2 | 89 | 114 | ***0.0154*** |
|  | G3+G4 | 134 | 108 |  |
| Stage | S1+S2 | 104 | 155 | ***<0.0001*** |
|  | S3+S4 | 119 | 67 |  |

Supplementary Table 37. The characteristics of PSMG3-AS1 in renal cell carcinoma.

| **Characteristic** | | **lncRNA-PSMG3-AS1** | | **P-value** |
| --- | --- | --- | --- | --- |
|  |  | **Low(n=223)** | **High(n=222)** |  |
| Age (years) | <=60 | 110 | 109 | 0.9616 |
|  | >60 | 113 | 113 |  |
| Gender | Female | 81 | 79 | 0.8713 |
|  | Male | 142 | 143 |  |
| T | T1+T2 | 118 | 157 | ***0.0001*** |
|  | T3+T4 | 105 | 65 |  |
| N | N0 | 214 | 217 | 0.2812 |
|  | N1 | 9 | 5 |  |
| M | M0 | 182 | 192 | 0.1605 |
|  | M1 | 41 | 30 |  |
| Grade | G1+G2 | 86 | 117 | ***0.0028*** |
|  | G3+G4 | 137 | 105 |  |
| Stage | S1+S2 | 111 | 148 | ***0.0003*** |
|  | S3+S4 | 112 | 74 |  |

Supplementary Table 38. Enrichments of biological process (GO) functional pathways.

| **LncRNAs** | **Total number** | **Term description** |
| --- | --- | --- |
| AP000439.2  AC124854.1  TMEM246-AS1  LINC01510 | 18 | carboxylic acid catabolic process, small molecule metabolic process,  urate metabolic process, organic substance transport,  fatty acid catabolic process, anion transmembrane transport,  cellular amino acid metabolic process, organic acid metabolic process,  fatty acid metabolic process, alpha-amino acid metabolic process,  cellular lipid metabolic process, monocarboxylic acid catabolic process,  fatty acid oxidation, carboxylic acid metabolic process,  organic anion transport, monocarboxylic acid metabolic process  fatty acid beta-oxidation, small molecule catabolic process |
|  |  |  |
| AC124854.1  AP000439.2  TMEM246-AS1 | 20 | neurotransmitter metabolic process, drug transport,  sodium ion transmembrane transport, localization,  carboxylic acid transmembrane transport, ion transport,  organic hydroxy compound metabolic process, transport,  ion transmembrane transport, cation transmembrane transport,  drug transmembrane transport, carboxylic acid transport,  response to drug, carboxylic acid biosynthetic process,  cation transport, transmembrane transport,  neurotransmitter biosynthetic process, xenobiotic metabolic process,  modified amino acid transport, anion transport |
|  |  |  |
| AC124854.1  AP000439.2  LINC01510 | 1 | lipid modification |
|  |  |  |
| AP000439.2  LINC01510  TMEM246-AS1 | 8 | oxidation-reduction process, small molecule biosynthetic process,  carbohydrate metabolic process, cofactor metabolic process,  monosaccharide metabolic process, monosaccharide catabolic process,  organic substance catabolic process, carbohydrate catabolic process |

Supplementary Table 39. Enrichments of molecular function (GO) functional pathways.

| **LncRNAs** | **Total number** | **Term description** |
| --- | --- | --- |
| AP000439.2  AC124854.1  TMEM246-AS1  LINC01510 | 6 | anion binding, cofactor binding,  secondary active transmembrane transporter activity,  transmembrane transporter activity, transporter activity,  organic anion transmembrane transporter activity |
|  |  |  |
| AC124854.1  AP000439.2  TMEM246-AS1 | 9 | cation transmembrane transporter activity,  monocarboxylic acid transmembrane transporter activity,  carboxylic acid transmembrane transporter activity,  ion transmembrane transporter activity,  solute:sodium symporter activity, symporter activity,  inorganic molecular entity transmembrane transporter activity,  anion transmembrane transporter activity,  drug transmembrane transporter activity |
|  |  |  |
| AP000439.2  LINC01510  TMEM246-AS1 | 7 | catalytic activity, oxidoreductase activity,  urate transmembrane transporter activity,  oxidoreductase activity, acting on CH-OH group of donors,  coenzyme binding, iron ion binding,  sodium-independent organic anion transmembrane transporter activity |

Supplementary Table 40. Enrichments of KEGG functional pathways.

| **LncRNAs** | **Total number** | **Term description** |
| --- | --- | --- |
| AP000439.2  AC124854.1  TMEM246-AS1  LINC01510 | 4 | Fatty acid degradation,  Valine, leucine and isoleucine degradation,  beta-Alanine metabolism,  Metabolic pathways |

Supplementary Table 41. Enrichments of Reactome functional pathways.

| **LncRNAs** | **Total number** | **Term description** |
| --- | --- | --- |
| AP000439.2  AC124854.1  TMEM246-AS1  LINC01510 | 4 | Metabolism,  Biological oxidations,  SLC-mediated transmembrane transport,  Fatty acid metabolism |
| AC124854.1  AP000439.2  TMEM246-AS1 | 4 | Phase I - Functionalization of compounds,  Transport of small molecules,  SLC transporter disorders,  Transport of bile salts and organic acids, metal ions and amine compounds |
| AP000439.2  LINC01510  TMEM246-AS1 | 4 | Synthesis of Leukotrienes (LT) and Eoxins (EX),  Metabolism of amino acids and derivatives,  Metabolism of lipids,  Fatty acids |
